# Gut microbes shape microglia and cognitive function during malnutrition

**DOI:** 10.1002/glia.24139

**Published:** 2022-01-12

**Authors:** Kylynda C. Bauer, Elisa M. York, Mihai S. Cirstea, Nina Radisavljevic, Charisse Petersen, Kelsey E. Huus, Eric M. Brown, Tahereh Bozorgmehr, Rebeca Berdún, Louis‐Philippe Bernier, Amy H. Y. Lee, Sarah E. Woodward, Zakhar Krekhno, Jun Han, Robert E. W. Hancock, Victoria Ayala, Brian A. MacVicar, Barton Brett Finlay

**Affiliations:** ^1^ Michael Smith Laboratories University of British Columbia Vancouver Canada; ^2^ Microbiology and Immunology Department University of British Columbia Vancouver Canada; ^3^ Psychiatry Department, Djavad Mowafaghian Centre for Brain Health University of British Columbia Vancouver Canada; ^4^ Biochemistry and Molecular Biology Department University of British Columbia Vancouver Canada; ^5^ Institut de Recerca Biomèdica de Lleida (IRB‐Lleida) Lleida Spain; ^6^ Department of Experimental Medicine Universitat de Lleida (UdL) Lleida Spain; ^7^ The Metabolomics Innovation Centre University of Victoria Victoria Canada

**Keywords:** behavior, gut‐brain axis, malnutrition, microbiome, microglia, neurometabolism

## Abstract

Fecal‐oral contamination promotes malnutrition pathology. Lasting consequences of early life malnutrition include cognitive impairment, but the underlying pathology and influence of gut microbes remain largely unknown. Here, we utilize an established murine model combining malnutrition and iterative exposure to fecal commensals (MAL‐BG). The MAL‐BG model was analyzed in comparison to malnourished (MAL mice) and healthy (CON mice) controls. Malnourished mice display poor spatial memory and learning plasticity, as well as altered microglia, non‐neuronal CNS cells that regulate neuroimmune responses and brain plasticity. Chronic fecal‐oral exposures shaped microglial morphology and transcriptional profile, promoting phagocytic features in MAL‐BG mice. Unexpectedly, these changes occurred independently from significant cytokine‐induced inflammation or blood–brain barrier (BBB) disruption, key gut‐brain pathways. Metabolomic profiling of the MAL‐BG cortex revealed altered polyunsaturated fatty acid (PUFA) profiles and systemic lipoxidative stress. In contrast, supplementation with an ω3 PUFA/antioxidant‐associated diet (PAO) mitigated cognitive deficits within the MAL‐BG model. These findings provide valued insight into the malnourished gut microbiota‐brain axis, highlighting PUFA metabolism as a potential therapeutic target.

## Main Points

- Early‐life malnutrition and specific gut microbes shape cognition and microglia in mice.
- Altered microglial features occur independently from significant neuroinflammation or BBB deficits, but were linked to impaired lipid metabolism.
- PAO diet (ω3 PUFA/antioxidant‐associated supplement) improves cognitive features of malnutrition.

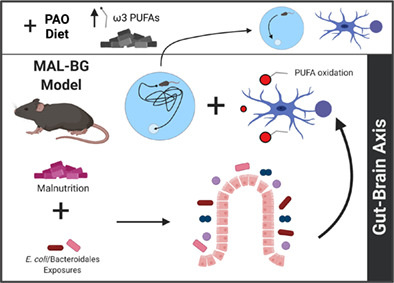

## 1 INTRODUCTION

Undernutrition affects one‐tenth of the global population (Roser & Ritchie, 2019). Nearly 150 million children under the age of five exhibit stunting (low height‐for‐age) and over 49 million children exhibit wasting (low weight‐for‐age) (UNICEF/WHO/World Bank Group, 2019). The ongoing COVID‐19 pandemic is anticipated to significantly exacerbate malnutrition, disrupting societal and environmental factors contributing to poverty (Akseer et al., 2020; Littlejohn & Finlay, 2021; Osendarp et al., 2021).

Interdependent societal (e.g., political instability, socioeconomic status) and environmental factors not only promote poverty, but also contribute to the biological consequences of malnutrition (Black et al., 2013; Blanton, Barratt, et al., 2016; Smith & Haddad, 2015). Environmental factors range from climate change to poor sanitation, water quality, and/or hygiene practices (Blanton, Barratt, et al., 2016; Phalkey et al., 2015; Tofail et al., 2018). These latter conditions promote microbial fecal‐oral contamination and subsequent gastrointestinal (GI) insult. Chronic GI dysbiosis and nutrient deficiency form a deadly cycle of deteriorating nutritional status and health, linked to childhood stunting and lasting immune, metabolic, and cognitive impairment within malnourished communities (Black et al., 2013; Blanton, Barratt, et al., 2016; Di Giovanni et al., 2016; Guerrant et al., 2013). Despite continued progress in the pathology and treatment of malnutrition, the precise role of fecal‐oral contamination remains largely unexplored.

Reported models of undernutrition include protein‐energy malnutrition (Muzi‐Filho et al., 2013), maternal malnutrition (Holemans et al., 2003), micronutrient deficiencies (Keen et al., 2003), and designer diets replicating the nutritional sources of specific, undernourished communities (Blanton, Barratt, et al., 2016). More recently, researchers have utilized gnotobiotic rodent models, demonstrating a causal role for the malnourished microbiota in physical stunting and metabolic shifts. Fecal microbiota transplantation of the early‐life malnourished microbiota promoted stunting and metabolic shifts in gnotobiotic mice (Blanton, Charbonneau, et al., 2016), while microbiome‐targeted therapies improved growth and neurodevelopmental markers, notably neurotrophin proteins, in both murine models and a randomized, double‐blind clinical trial (Gehrig et al., 2019). Indeed, gut microbes likely influence neurological pathology of malnutrition. Recent studies have linked the dysbiotic (altered) gut microbiota to blood–brain barrier (BBB) deficits (Braniste et al., 2014), aberrant neuroinflammation (Fung et al., 2017; Sampson et al., 2016), and disrupted neurodevelopment (Borre et al., 2014; Erny et al., 2015).

We specifically assessed the impact of fecal‐oral contamination and malnutrition on microglia, non‐neuronal cells whose functionality and maturation are shaped by commensal microbes (Erny et al., 2015). As resident macrophages of the CNS, microglia extend motile processes into the neural environment (brain parenchyma) dynamically responding to environmental cues (Nimmerjahn et al., 2005; Wu et al., 2015). Microglia regulate neuroinflammatory responses, counter pathogens, and scavenge debris through dynamic phagocytic processes (Brown & Neher, 2014; Prinz & Priller, 2014; Wu et al., 2015; York, Bernier, & MacVicar, 2018). Microglia phagocytosis also shapes neurodevelopment and brain plasticity. During development, microglia actively prune neuronal synapses to promote appropriate communication along neural networks. Microglia continue to sculpt cognitive capacity throughout adulthood, shaping synaptic strength and plasticity (Wu et al., 2015; York, Bernier, & MacVicar, 2018). Microglial dysfunction, consequently, contributes to the etiology and/or pathology of many neural disorders (Prinz & Priller, 2014; Sampson et al., 2016).

Here, we utilized well‐established malnutrition models, MAL and MAL‐BG, fed a protein/fat‐deficient and carbohydrate‐rich diet, deficits frequently reported among undernourished communities (Bauer et al., 2020; Brown et al., 2015; Huus et al., 2020; Nr et al., 1990). In addition to poor diet, MAL‐BG mice received iterative fecal microbial exposures (**mal**nutrition + **b**acterial **g**avage of *Escherichia coli*/Bacteroidales). These commensals were associated with growth deficits within independent murine models and malnourished, pediatric cohorts (Brown et al., 2015; Kau et al., 2015; Li et al., 2019; Robertson et al., 2019).

We report altered cognitive, microglial, and microbiome features in malnourished mice exposed to fecal‐oral contamination. MAL‐BG microglia exhibit impaired morphology, transcriptional profile, and phagocytic features. These microglial alterations occur independently from significant BBB deficits or elevated proinflammatory cytokines (TNF‐α, IL‐6) within the CNS. Metabolomic profiling, however, revealed shifts in polyunsaturated fatty acid (PUFA) metabolism and lipoxidative stress within the MAL‐BG CNS. Moreover, supplementation with ω3 PUFAs and vitamin antioxidants (PAO diet) improved cognitive deficits in the MAL‐BG model. Collectively, our results highlight dynamic microglial responses to commensal microbes and diet, identifying fatty acid metabolism as a potential gut‐brain pathway informing neurocognitive consequences of childhood malnutrition.

## 2 METHODS

### 2.1 Mouse studies

Three‐week‐old female C57BL/6J mice (Jackson Laboratory, Bar Harbor, ME) were housed in the Modified Barrier Facility at the University of British Columbia on a 12‐h light–dark cycle. On arrival, mice were randomized and housed in separate groups (3–5 per group, ventilated cages with wood chip bedding and enrichment). Mice were fed either a standard mouse chow (D09051102: Research Diets, New Brunswick, NJ; Supplemental File 1) or an isocaloric low‐protein/fat diet (D14071001). While malnutrition refers to any deviation from healthy nutritional status, undernutrition specifies nutritional deficiency. Despite protein and fat deficiencies, the D14071001 diet has standard caloric content. To maintain consistent naming with earlier publications, mice fed this diet are referenced as malnourished or MAL (Bauer et al., 2020; Brown et al., 2015; Huus et al., 2020). Mice received ad libitum chow and water.

To assess microglia morphology and motility (Figure 2 and Figure S3), we utilized weaned male and female CX3CR1^+/EGFP^ mice ranging 3–6 weeks old at initiation of trial on C57BL/6 background (Jung et al., 2000), which were bred and housed at the Animal Resource Unit facility at the University of British Columbia. Apparent sex differences were not observed in the study. The microglia analyses for the PAO intervention trial utilized newly‐weaned female C57BL/6 mice housed at the Modified Barrier Facility. All animal work was done in accordance with the Animal Care Committee at the University of British Columbia and the Canadian Council on Animal Care guidelines.

### 2.2 MAL‐BG model

To elicit MAL‐BG features, a subgroup of mice on the low‐protein diet was exposed to a cocktail of seven commensal bacteria as previously reported (Brown et al., 2015). Original bacterial strains were provided by Emma Allen‐Vercoe (University of Guelph). Briefly, frozen stocks of bacterial cultures were plated on FAA in anaerobic conditions. Bacteria were mixed in a 1:1 ratio in sterile, reduced PBS for oral gavage (100 μl, 10^9^cells/ml). Three gavages were administered over a period of 5 days 2 weeks following initiation of the experimental diets. To control for gavage stress, non‐MAL‐BG mice received PBS gavages over the same period.

### 2.3 PAO intervention

To assess whether MAL‐BG features could be reversed, ω3 PUFA‐enriched, antioxidant‐associated (PAO) diets with were developed. The CON‐PAO and MAL‐PAO diets matched CON (D09051102) and MAL (D14071001) diets, respectively, in caloric value and carbohydrate/protein content. PAO diets were supplemented with vitamin antioxidants (vitamin E, ascorbic acid). Menhaden (fish) oil (ω3 high) replaced soybean oil (ω6 high), as the dietary fat source. Four groups of mice were analyzed for antioxidant experiments: CON, MAL‐BG, CON‐PAO, and MBG‐PAO (MAL‐BG model on the MAL‐PAO diet). Full dietary breakdown provided in Supplemental File 1. Mice were placed on the diets at 3 weeks of age and maintained on PAO intervention for 33 days. Mice received ad libitum chow and water.

### 2.4 Mouse behavioral tests

Testing order (CON, MAL, MAL‐BG, CON‐AO, or MBG‐PAO) was randomized prior to behavioral tests. Mouse movements were recorded via Go‐PRO (HERO 4, HERO Black 6). Tracking and scoring videos were analyzed by blinded analysis with ANY‐Maze software or a blinded observer.

#### 2.4.1 Open field test

The open field test (OFT) measures rodent locomotion and anxiety‐like behavior. Mice were placed in an OFT box (49 Liter Tote, Home Depot: 39.4 × 56.4 × 31.8 cm^3^). The base of the box was divided into a grid with a defined open field (22 × 11 cm^2^). Individual mice were placed in the OFT box for 5 min and allowed to explore freely. The OFT box was cleaned with 70% ethanol between use.

#### 2.4.2 Novel object recognition test (NORT)

To assess exploratory behaviors, two identical objects were placed in the OFT box at opposing sides. Individual mice were allowed to freely explore objects for a 3 min habituation phase. Following a 3‐h delay period, one object was replaced by a distinct, yet similarly sized object (novel object). Individual mice were returned to the OFT for a 3 min test phase. A blinded observer recorded interaction times. For this test, we defined mouse interaction as sniffing and/or placing the snout on the object.

#### 2.4.3 Light–dark test

Mice were placed in a 10.5 × 34.5 cm^2^ light–dark box (one third light zone, two third dark zone). Animals were allowed to freely explore for 3 min, the light–dark box was cleaned with 70% ethanol between use.

#### 2.4.4 Morris water maze

**FIGURE 1:**
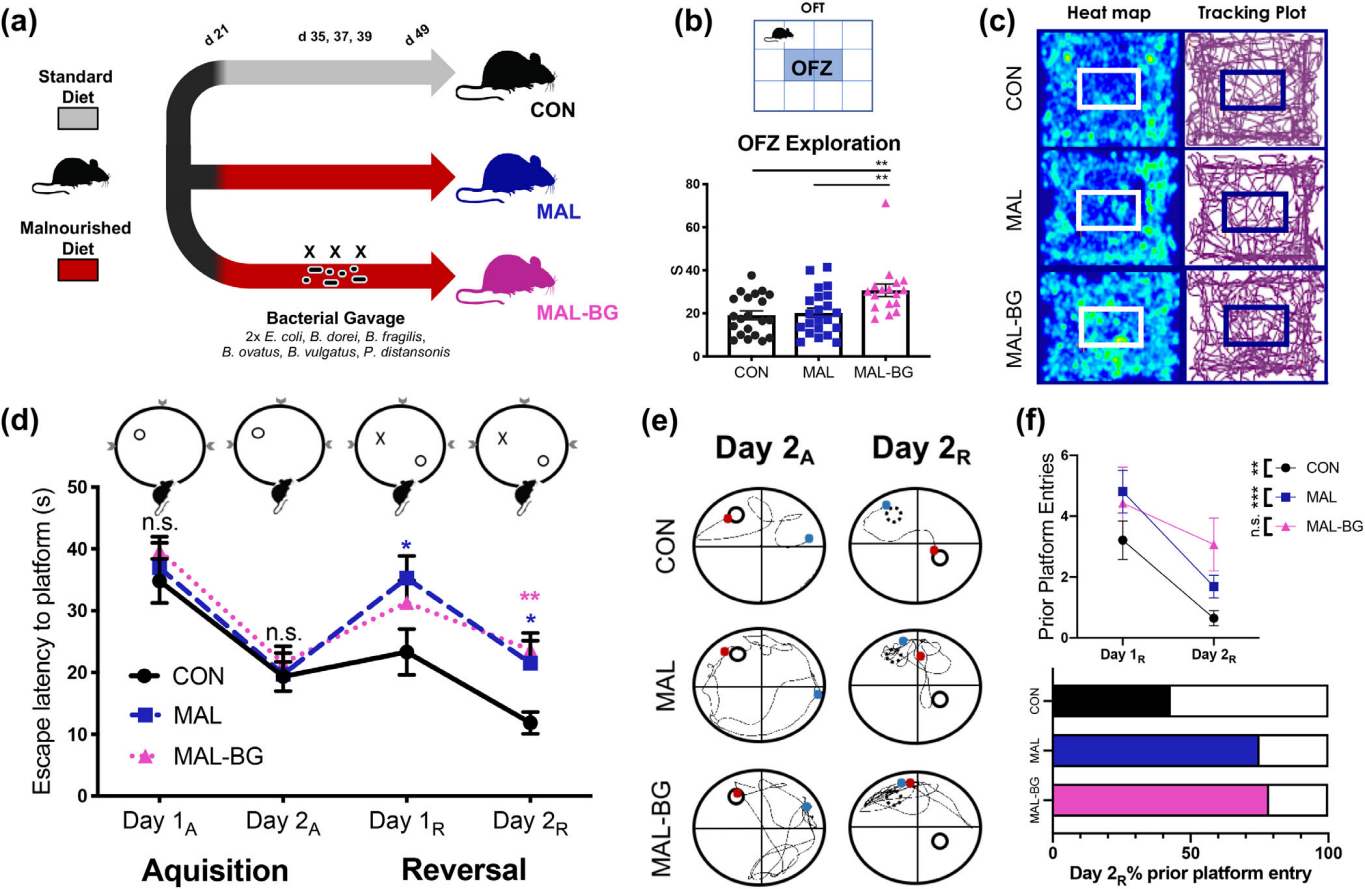
Fecal‐oral contamination alters behavior and cognition during malnutrition. (a) MAL‐BG timeline (adapted from Brown et al., 2015). Weaned (21‐day old) mice are placed on a standard mouse chow (20% protein, 15% fat) or malnourished diet (7% protein, 5% fat). To model fecal‐oral contamination, malnourished mice received three bacterial gavages across 5 days (MAL‐BG model). (b) OFT set‐up with OFZ highlighted in blue (top), pooled data from three independent experiments. Time spent within the OFZ (bottom); *n* = 21 CON, 22 MAL, 17 MAL‐BG. (c) Representative heatmap and tracking plots displaying OFT exploration patterns (5 min). (d–f) MWMT, pooled results from two independent experiments, *n* = 13–16 per group. Detailed MWMT procedures provided in Section 2 and Figure S2f. (d) We report averaged escape latencies of individual mice (four trials/d) in acquisition = _A_ and reversal = _R_ sessions. (e) Representative swim paths from the final acquisition and reversal trial: solid circle = platform location, dotted circle = prior platform location, blue dot = start position, and red dot = final position. (f) Total prior platform entries of mice on Day 1_R_ and Day 2_R_, statistical significance determined by a two‐tailed, paired t‐test. Percent of mice in each group that returned to the prior platform location on Day 2_R_ (bottom). OFT and MWMT assessments were conducted with blinded ANY‐maze software tracking. Graphs indicate mean and SEM and statistical significance for Figure 1a–e determined by one‐way ANOVA with post hoc Tukey's test (OFT) or post hoc Dunnett's test (MWMT); OFT, open field test; OFZ, open field zone; MWM/T, Morris water maze/test; n.s., non‐significant

The Morris water maze (MWM) was utilized to assess learning and spatial memory in mice. Testing occurred at the University of British Columbia Modified Barrier Facility. Mice were tested in a pool ~116 cm diameter (water temperature, 21–23°C). The testing arena was supplied with indirect light with the MWM pool surrounded by distal visual cues. A circular platform (11 cm diameter) was used as the goal platform (see Figure S2f). Fecal droppings were removed from the platform between trials. After testing, mice were gently dried and placed in a warming cage prior to returning to their home cage. Platform and animal start positions were randomly determined for each of the training and testing days. Certain mice in MWM tests were removed due to video recorder malfunction and/or prolonged swim refusal (≤3 mice/trial of 48 total mice [Figure 1] or 32 total mice [Figure 5]). All mouse removals were selected during blinded analyses.

#### 2.4.5 Visible platform training (1 day, four trials)

Fixed mouse start position/variable platform location—in this habituation day, individual mice were given 60 s to locate the visible goal platform (opaque top, 1–1.5 cm above water). Mice that failed to climb the platform within 60 s were gently guided onto the goal platform. To promote spatial memory, mice were given a 30 s rest period on the platform between successive trials. All mice were able to recognize the platform by the end of training.

#### 2.4.6 Acquisition training (2 days, 12 trials)

Variable mouse start position/fixed platform location—the goal platform was not visible (clear top, 0.5 cm submerged) during acquisition periods. Individual mice were given 60 s to locate the goal platform. Mice that failed to locate the platform were gently guided onto the platform following the trial period. Mice were allowed to briefly rest on the platform for the first four consecutive trials of each day. In remaining trials, mice were immediately removed after locating the platform. Only the first four trials each day were recorded. PAO intervention mice underwent a maximum of four trials.

#### 2.4.7 Free swim 1 (1 day)

Variable mouse start position/platform removed—mice were allowed to freely explore the pool during a 30 s probe run. PAO mice did not undergo any free swim trials.

#### 2.4.8 Acquisition training reversal (2 day, 12 trials)

Variable mouse start position/fixed platform location—before training, the goal platform was moved to a different quadrant. Protocol follows initial acquisition training.

#### 2.4.9 Free swim 2 (1 day, 1 probe)

Same protocol as initial free swim. Free swims occurred 24 h following the final acquisition trial.

### 2.5 Ex vivo cytokine profiling

Following euthanasia, whole brain tissues were collected within individual Eppendorf tubes containing 1 ml dPBS and cOmplete™ EDTA‐free Protease Inhibitor, prior to storage at −70/80°C or immediately homogenized using a Retsch MM 301 Mixer Mill or FastPrep®‐24 (MP Biomedicals) 2x for 1 min using tungsten beads. Collected blood was spun at 6000 *g* for 8 min to obtain sera. Cytokine measurements from tissue supernatant and sera were obtained with the BD Biosciences Cytometric Bead Array Mouse Inflammation Kit. Cytokine measurements from whole brain samples were normalized to tissue weight.

### 2.6 Acute hippocampal slice preparation

CX3CR1^+/EGFP^ C57BL/6 mice were decapitated and brains were dissected and sliced horizontally with a vibratome (Leica VT1200S) to 300 μm thick in ice‐cold NMDG slicing solution containing (in mM): 120 N‐methyl‐d‐glucamine, 2.5 KCl, 25 NaHCO_3_, 1 CaCl_2_, 7 MgCl_2_, 1.2 NaH_2_PO_4_, 20 d‐glucose, 2.4 sodium pyruvate, and 1.3 sodium L‐ascorbate, which was constantly oxygenated with 95% O_2_ and 5% CO_2_. Hippocampal slices were immediately transferred to artificial cerebral spinal fluid (aCSF), which was continuously oxygenated with 95% O_2_ and 5% CO_2_. The aCSF contained (in mM): 126 NaCl, 2.5 KCl, 26 NaHCO_3_, 2 CaCl_2_, 2 MgCl_2_, 1.25 NaH_2_PO_4_, and 10 d‐glucose, pH 7.3–7.4, osmolarity 300 mOsm. Slices were recovered in aCSF at 32°C for a minimum of 30 min before imaging for time‐lapse and lesion experiments or before fixation by the SNAPSHOT protocol (Dissing‐Olesen & MacVicar, 2015). Briefly, this involved a 2‐min fixation in 4% paraformaldehyde at 80°C, followed by a PBS wash, and storage in clearing solution (20% DMSO and 2% Triton X‐100 in PBS) at 4°C.

### 2.7 Two‐photon microscopy, time‐lapse imaging, and lesion analysis

Acute slices from CX3CR1^+/EGFP^ C57BL/6 mice were imaged immediately after recovery using a Coherent Chameleon Ultra II laser (mode‐locked pulse train at 80 MHz at 920 nm) with a Zeiss LSM 7 MP microscope and Zeiss 20x‐W/1.0 NA objective. Green fluorescence was detected by a 520/60 nm filter (Chroma tech) and GaAsP photo‐multiplier tube (PMT; Zeiss LSM BiG). Images were acquired as a z‐stack (zoom factor 2.8; 151.82 × 151.82 μm xy scale, 8‐line averaging) 18 μm thick, centered approximately 150 μm below the slice surface (2 μm slice interval) in the stratum radiatum region of the CA1 hippocampus. Following a 10‐min baseline imaging period, a lesion was created by focusing the laser to the region of interest and scanning at 800 nm at 100% power for approximately 30 s. Microglial response to this lesion was then imaged for an additional 15 min using the same imaging parameters as baseline.

For motility analysis, baseline movies were maximum projected and loaded into a custom MATLAB program. This program quantifies the number of new pixels (additions) and number of removed pixels (retractions) across time as the Motility Index. To quantify the microglial response to lesion, a circular region of interest with a diameter of 30 μm was centered on the lesion response region, and the mean intensity was measured at each frame.

### 2.8 3D‐morph and phagocytic cup quantification

EGFP is well preserved by the SNAPSHOT protocol, and these slices were ready to image immediately after 1‐week incubation in clearing solution at 4°C. By two‐photon microscopy, a z‐stack at 1024 × 1024 (zoom factor 1.5; 283.12 × 283.12 μm xy scale, 16‐line averaging) from 125–175 μm deep (2 μm slice interval) was acquired. Using these images, 3D‐Morph MATLAB analysis was completed as previously reported (York, LeDue, et al., 2018) to quantify microglial morphologies. Before analysis, all images were processed by background subtraction in Fiji, and all treatment rounds were batch processed using the same analysis parameters. From these morphological images, the number of phagocytic cups was manually counted.

### 2.9 Blood–brain barrier integrity: IgG and biocytin

To investigate BBB permeability, 100 μl TMR Biocytin (AnaSpec AS‐60658; reconstituted with sterile PBS; MW = 869 Da) was delivered by tail‐vein injection to mice 20 min prior to cardiac perfusion. Following brain dissection and coronal slicing (300 μm thick by vibratome), tissues were imaged using a Zeiss Axio Zoom microscope with TMR emission filter settings. Fluorescence intensity was measured from slices spanning the entire rostral‐caudal area of the brain. Mean intensity was compared across treatments.

As an additional permeability measure, slices were stained for anti‐mouse IgG, which should not be present in the brain parenchyma. For staining, thick slices were cleared (20% DMSO and 2% Triton X‐100 in PBS) for 1 week, blocked in 4% normal goat serum overnight at room temperature, and incubated with Alexa Fluor 488 goat anti‐mouse IgG for 6 days at 4°C. After four, 1‐h washes in PBS at room temperature, the tissue was imaged by two‐photon microscopy using a 20x‐W/1.0 NA objective and 5× zoom factor. The mean fluorescence intensity was averaged across three separate images per slice, and compared between mice.

### 2.10 RNA‐SEQ analysis

Whole mouse cerebra were stored on ice in RPMI growth media prior to tissue dissociation. Tissues were dissociated via the Adult Brain Dissociation kit with the gentleMACS™ Octo Dissociator with Heaters (program: 37C_ABDK_01) from Miltenyi Biotec. Following dissociation, microglia was enriched through magnetic separation with CD11b MicroBeads and MidiMACS™, according to Miltenyi Biotec protocols. Microglia made up ~90% of CD11b+ samples as determined by flow cytometry (Figure S4a). Enriched microglia samples were stored in RNA later prior to RNA isolation with RNEasy Micro Kit (QIAGEN).

Samples were sent to the BRC Sequencing Core at UBC. Prior to RNA‐Seq, sample quality control was performed via the Agilent 2100 Bioanalyzer. Qualifying samples were prepared according to established protocols for the NEBnext Ultra II Stranded mRNA (New England Biolabs). Sequencing was performed on the Illumina NextSeq 500 with Paired End 42 × 42 bp reads and demultiplexed with Illumina's bcl2fastq2. Demultiplexed read sequences were aligned to the *Mus musculus* reference sequence GRCm38.p6 (Zerbino et al., 2018) using STAR v. 2.6.1d, followed by read‐count generation using HTSeq v. 0.11.2 (Anders et al., 2015). Differential gene expression was estimated with DeSEQ2 v. 3.9 with further pathway analyses conducted using the ReactomePA pipeline, as described (Love et al., 2014; Yu & He, 2016). Analyses were conducted with R (v. 3.5.1). Code provided in attached Glia_RMarkdown (Supplementary information). Raw and processed data files were deposited to the NCBI gene expression omnibus (GEO): GEO accession GSE138182.

### 2.11 qPCR

RT‐qPCR analysis was performed using QuantiTect SYBR Green PCR Master Mix (Qiagen) from ileum or whole brain (cortical) tissue using the following primers, *Ctsd* (F: GACATCTCTTCTGGTGGGGC, R: GGCTGGACACCTTCTCACAA), *Gapdh* (F: ATTGTCAGCAATGCATCCTG, R: ATGGACTGTGGTCATGAGCC), *Hprt* (F: GATTAGCGATGATGAACCAGGTT, R: CCTCCCATCTCCTTCATGACA), *Sirpa* (F: TCCGCGTCCTGTTTCTGTAC, R: TTCAGAACGGTCGAATCCCC), and *Tjp1* (F: CCCTGAAAGAAGCGATTCAG, R: CCCGCCTTCTGTATCTGTGT) based on established PCR protocols (Brown et al., 2015). *Gapdh* and *Hprt* provided an endogenous control for gut (*Tjp1*) and microglial genes of interest, respectively, and were used for normalization. ddCT calculations provided relative expression to control samples.

### 2.12 Flow cytometry

Microglia cells were isolated through Percoll gradient or Miltenyi Adult Brain Dissociation kit, as described in RNA‐Seq Analysis. Microglia staining occurred in 1X dPBS^−/−^ (Thermo Fischer) supplemented with 0.5% FBS, 0.4% 0.5 M EDTA, and 1% HEPES at 4°C for 20 min. Cells were stained with the following antibodies: anti‐CD11b (clone:M1/70, eBioscience), anti‐CD45 (clone:30‐F11, eBioscience), anti‐F480 (clone:BM8, eBiosience), anti‐CX3CR1 (clone:SA011F11, Biolegend), anti‐CD31 (clone:MEC13.3, Biolegend), anti‐CCR3 (clone:J073E5, Biolegend), anti‐ I‐A/I‐E (clone:M5/114.15.2, Biolegend), anti‐CD80 (clone:16‐10A1, eBioscience), anti‐CD86 (clone:GL1, eBioscience), and anti‐TLR4 (clone:SA15‐21, Biolegend). Following staining, cells were washed twice and fixed in a 1:1 solution of supplemented dPBS^−/−^: 4% paraformaldehyde overnight at 4°C. After fixation, cells were resuspended in supplemented dPBS^−/−^and enumerated via flow cytometry (BD LSR II with 561 laser). Microglia populations were identified as CD11b^high^/CD45^low^. Subsequent data was analyzed using FlowJo software (v. 10.5.3).

### 2.13 Metabolomics

Mouse hippocampal tissues were collected for untargeted reversed‐phased ultrahigh performance liquid chromatography–Fourier transform mass spectrometry (RP‐UPLC‐FTMS) metabolomics analysis. Tissue samples were kept in dry ice prior to storage at −70/80°C. Metabolomics were completed by TMIC (The Metabolomics Innovation Centre).

#### 2.13.1 Metabolite extraction

Each mouse hippocampal sample in an Eppendorf tube was mixed with water; 5 μl per mg of the tissue, and two 4‐mm metal balls were added. The tissue was homogenized on a MM 400 mill mixer at a vibrating frequency of 30 Hz for 1 min twice. After 5‐s spin‐down, a mixture of methanol‐chloroform (4:1) was added, at 25 μl per mg tissue, to each tube. The sample was homogenized again for metabolite extraction using the same setup for 1 min twice, followed by sonication in an ice‐water bath for 5 min. The tube was centrifuged at 15,000 rpm at 10°C for 20 min. The clear supernatant was transferred to a 1.5‐ml Eppendorf tube. A 60‐μl aliquot from each sample was dried down inside the same nitrogen evaporator and the residue was reconstituted in 40 μl of 80% methanol. 10 μl was injected for RP‐UPLC‐FTMS. Two rounds of sample injections were made, with positive‐ and negative‐ion detection, respectively.

#### 2.13.2 RP‐UPLC‐FTMS analysis

A Dionex Ultimate 3000 UHPLC system coupled to a Thermo LTQ‐Orbitrap Velos Pro mass spectrometer, equipped with electrospray ionization (ESI) source, was used. RP‐UPLC‐FTMS runs were carried out with a Waters BEH C8 column (2.1 × 50 mm^2^, 1.7 μm) for chromatographic separations. The mobile phase was (A) 0.01% formic acid in water and (B) 0.01% formic acid in acetonitrile‐isopropanol (1:1). The elution gradient was 5%–50% B in 5 min; 50%–100% B in 15 min; and 100% B for 2 min before column equilibration for 4 min between injections. The column flow was 400 μl/min while the column temperature was 60°C. For relative quantitation, the MS instrument was run in the survey scan mode with FTMS detection at a mass resolution of 60,000 full width at half maximum at *m/z* 400. The mass scan range was *m/z* 80–1800, with a reference lock‐mass for real‐time calibration. Two UPLC‐FTMS datasets were acquired for each sample, one with positive‐ion detection and the other with negative‐ion detection. LC–MS/MS data was also acquired from each sample set with collision‐induced dissociation at different levels of normalized collision energy.

#### 2.13.3 Data processing

Each LC‐FTMS dataset was respectively processed with XCMS (https://xcmsonline.scripps.edu/) in R for peak detection and two rounds of retention time (RT) shift correction, peak grouping, and peak alignment. Mass de‐isotoping and removal of chemical and electronic background peaks were performed with manual interventions. The output of data processing is the pairs of *m/z* (mass‐to‐charge ration), RT (min), and LC–MS peak areas of the detected metabolites or metabolite features across the samples for each set.

#### 2.13.4 Metabolomics analyses

To assign the metabolite candidates of any potential biomarkers, the measured *m/z*'s were searched against metabolome databases, including: LIPID MAPS (http://www.lipidmaps.org/tools/ms/lm_mass_form.php), METLIN (https://metlin.scripps.edu/metabo_batch.php), and/or HMDB (http://www.hmdb.ca/spectra/ms/search). During database searches, allowable mass errors were set at ≤3 ppm. For the (+) ion detection data, ion forms of (M + H)+, (M + Na)+, (M‐H_2_O + H)+, and (M‐NH_3_ + H) + were considered and ion forms of (M‐H)‐, (M + Na‐2H)‐, (M‐H_2_O‐H)‐, and (M‐NH_3_‐H)‐ were considered for (−) ion detection. Random forest, Principal component analysis (PCA)/Partial least squares discriminant analysis (PLSDA), and pathway analyses were carried out using Metaboanalyst v. 3.0/4.0 software (Chong & Xia, 2018) with the following parameters: mass tolerance = 0.0003, RT tolerance = 30, data was normalized against pooled CON, data transformation = log‐transformation, and data scaling = auto data scaling. Over‐representation analysis determined over‐represented pathways in each group. Analyses were conducted based on previous analyses (Brown et al., 2015). A one‐way ANOVA was used to determine significant changes across groups (*Padj* < 0.05; fold change >2).

### 2.14 Brain inflammatory and fatty acid profile

Whole brains were collected and rapidly stored at −70/80°C prior to analyses. Tissues were homogenized with Ultra‐Turrax (3420000 IKA, Germany) in homogenization buffer (180 mM KCl, 5 mM MOPS, 2 mM EDTA, 1 mM DTPA, and 1 uM 2,6‐di‐tert‐butil‐4‐metilfenol; pH 7,4). Homogenates were normalized by protein content as determined by Bradford assay.

#### 2.14.1 Fatty acid preparation

Total lipids from homogenates (50–150 mg) tissue were extracted with chloroform/methanol (2:1 vol/vol; 3×) in the presence of 0.01% butylated hydroxytoluene. The resulting chloroform phase was evaporated under nitrogen. After lipid extraction, fatty acyl groups were analyzed as methyl esters derivatives by gas chromatography (GC). Briefly, fatty acids were transesterified at 75°C for 90 min through incubation in 2 ml of 5% methanolic. The resulting fatty acid methyl esters (FAMEs) were extracted by adding 2 ml of n‐pentane and 1 ml of saturated NaCl solution. The separated n‐pentane phase was evaporated under nitrogen and dissolved in 80 μl of carbon disulfide. Two μl of sample were used in GC analysis.

#### 2.14.2 GC method

Analyses were conducted using the GC System 7890A/Series Injector 7683B (Agilent, Barcelona, Spain) and a flame ionization detector/DBWAX capillary column (30 m length × 0.25 mm [inner diameter] × 0.20 μm [film thickness]). The injections were performed with the splitless mode at 220°C. The flow rate of carrier gas (helium 99.99%) was maintained at a constant rate of 1.8 ml/min. The column temperature was held at 145°C for 5 min, increased by 2°C/min to 245°C for 50 min, and held at 245°C for 10 min with a post‐run of 250°C for 10 min.

#### 2.14.3 Data analysis

Identification of the 25 FAMEs was made with authenticated standards (Larodan Fine Chemicals, Malmö, Sweden). Results were expressed as %mol and then normalized to CON. The fatty acid profile detected, identified, and quantified represents more than 95% of the total chromatogram. The following fatty acid indexes were calculated: PUFAs from ω3 and ω6 series (PUFA ω3 and PUFA ω6) and a pro‐inflammatory index (ω6/ω3):(PUFA ω6/PUFA ω3).

#### 2.14.4 GC/MS measurement of oxidative stress markers

Glutamic semialdehyde (GSA/HAVA), Nε ‐(carboxyethyl)‐lysine (CEL), Nε ‐(carboxymethyl)‐lysine (CML), S‐(carboxymethyl)‐cysteine (CMC), and Nε‐(malondialdehyde)‐lysine (MDAL) concentrations in total proteins from whole brain and liver homogenates were measured by gas chromatography/mass spectrometry (GC/MS) as described (Naudí et al., 2013; Pamplona et al., 2005).

Samples containing 0.5 mg of protein were delipidated as described above and proteins were precipitated by adding 10% trichloroacetic acid (final concentration) and subsequently centrifuged at 4400 rpm, 4°C, 15 min. Protein samples were reduced overnight with 500 mM NaBH4 (final concentration) in 0.2 M borate buffer, pH 9.2, containing 1 drop of hexanol as an anti‐foam reagent. To eliminate crystals, proteins were then reprecipitated by adding 1 ml of 10% trichloroacetic acid and subsequent centrifugation. The following isotopically labeled internal standards were then added: (2H8)lysine (12 nmols), (2H5)HAVA (72 pmols) (for GSA quantization), (2H4)CEL (144.1 pmols), (2H8)MDA‐lysine (20.6 pmols), (2H2)CML (162.2 pmols), and (13H2)CMC (112.4 pmols).

The samples were hydrolyzed at 155°C for 30 min in 1 ml of 6N HCl, and then concentrated by speed‐vac. The N,O‐trifluoroacetyl methyl ester derivatives (TFAMEs) of the protein hydrolysate were prepared as previously described (Knecht et al., 1991). Briefly, hydrolyzed samples were incubated in 1 ml of 5% acetyl chloride‐methanol solution. Methyl esters from hydrolyzed samples were incubated with 1 ml of trifluoroacetic anhydride acid for 1 h and then evaporated with nitrogen gas (Nevap Model 113 Organomation Association, Berlin, MA, EUA) to obtain TFAMEs from amino acids in the hydrolyzed solution. Finally, the samples were redissolved with 80 μl of dichloromethane as a vehicle for subsequent analysis by GC/MS. GC/MS analyses were carried out on a Hewlett‐Packard model 6890 gas chromatograph equipped with a 30 m HP‐5MS capillary column (30 m × 0.25 mm × 0.25 μm) coupled to a Hewlett‐Packard model 5973A mass selective detector (Agilent, Barcelona, Catalonia). The injection port was maintained at 275°C. Two μl of sample were injected for each run. The column temperature was held at 110°C for 5 min, then 2°C/min to 150°C, then 5°C/min to 240°C, then 25°C/min to 300°C, and finally held at 300°C for 5 min.

Quantification was performed by external standardization using standard curves constructed from mixtures of deuterated and non‐deuterated standards: lysine; (2H8)lysine; HAVA; (2H5)HAVA; CEL; (2H4)CEL; MDA‐lysine; (2H8)MDA‐lysine; CML; (2H2)CML; CMC; (13C2)CMC (PolyPeptide Group, Strasbourg, France; Sigma‐Aldrich, Madrid, Spain or donated by Dr. Requena). Analytes were detected by selected ion‐monitoring GC/MS. The following ions were utilized: lysine and d8‐lysine, *m/z* 180 and 187, respectively; 5‐hydroxy‐2‐aminovaleric acid and d5‐5‐hydroxy‐2‐aminovaleric acid (stable derivatives of GSA), *m/z* 280 and 285, respectively; CML and d4‐CML, *m/z* 392 and 396, respectively; CEL and d4‐CEL, *m/z* 379 and 383, respectively; CMC and d13‐2C‐CMC, *m/z* 271 and 273, respectively; and MDAL and d8‐MDAL, *m/z* 474 and 482, respectively. The amounts of products were expressed as the ratio μmol HAVA, CML, CEL, CMC, or MDAL per mol lysine.

### 2.15 Gut isolation and CellROX® assay

Intestinal epithelial cells (IECs) were harvested from CON, MAL, and MAL‐BG small intestinal tissue (ileum: 5 cm). After removal of luminal content, tissues were washed multiple times in PBS +/+ with 0.1% BSA. Individual tissues were then added to IEC buffer (PBS −/− with 5% FBS, 1 mM ethylenediaminetetraacetic acid, and 1 mM dithiothreitol). After 10 min at 37°C with shaking, individual samples were strained through a 70 μm strainer and then centrifuged at 1500 rpm. Cell pellets were resuspended in RPMI 1640 and IEC digestion was repeated again. Following the second digestion step isolated IECs were plated at 37°C (~15,000 cells/well) and stained using CellROX® Deep Red Reagent (final concentration 15 μM) for 30 min in the dark with shaking. Upon oxidation from reactive oxygen species (ROS), the CellROX® reagent becomes fluorescent (emission maxima ~665 nm), fluorescent intensity was measured via plate reader with RPMI blanks serving as a control.

### 2.16 Microbiome analyses

Fecal samples were collected from mice and kept in −70°C prior to isolation. Fecal DNA was released by boiling sample suspensions for 15 min at 100°C. Library preparation for 16S rRNA sequencing was then performed by Microbiome Insights according to a standardized pipeline (https://microbiomeinsights.com/itag-microbiome-analysis/). Briefly, PCR amplification of the 16S rRNA gene was performed using barcoded primers against the V4 region (Kozich, Schloss et al, 2013), with 2 μl of lysate as template. PCR amplicons were cleaned using a SequalPrep 96‐well plate kit (ThermoFisher A1051001) and were sequenced on a Miseq platform to obtain 2x250 bp reads. Microbiota analyses were conducted using the QIIME2 pipeline (v. 2018.2) with Deblur feature table construction. Visualization was created with RStudio (Version 1.1.463). Functional analyses conducted using PICRUSt (v2.1.3b) and annotated with MetaCyc (Caspi et al., 2014; Douglas et al., 2020). Raw sequencing data was deposited to the NCBI sequence read archive (SRA): SRA BioProject PRJNA574479 with PICRUSt output in Supplemental File 3.

To specifically quantify *E. coli* abundance, qPCR of extracted fecal DNA was performed using Enterobacteriaceae‐specific primers (F: CATTGACGTTACCCGCAGAAGAAGC, R: CTCTACGAGACTCAAGCTTGC), as described in (Brown et al., 2015). Universal bacterial 16S primers were used for normalization (F: ACTCCTACGGGAGGCAGCAGT, R: ATTACCGCGGCTGCTGGC). The relative abundance of Enterobacteriaceae was calculated using the formula 2^‐(X‐Y) where X = the mean Ct for the universal 16S reaction and Y = the mean Ct for the Enterobacteriaceae reaction. qPCR was performed on an Applied Biosystems 7500 machine, with QuantiTect SYBR Green PCR Kits (QIAGEN 204143), in 10 ul reaction volumes using 2 ul template DNA.

### 2.17 Statistical analysis

Datasets generated/analyzed in this project were deposited in appropriate online repositories. Any additional data that support the findings of this study are available from the corresponding author upon reasonable request. Statistical analyses were performed using GraphPad PRISM. Statistical significance was given as ****p < .0001, ***p < .001, **p < .01, *p < .05, and *Padj* = FDR correction. Analyses are expressed as the mean with SEM unless otherwise stated.

## 3 RESULTS

### 3.1 Behavior and cognitive plasticity altered in MAL‐BG mice

Upon weaning, C57BL/6J mice were randomized onto a standard diet (CON mice) or an isocaloric malnourished diet (MAL and MAL‐BG mice; Figure 1a). MAL‐BG mice received three gavages containing *E. coli*/Bacteroidales, a bacterial cocktail designed to model fecal‐oral contamination, a prevalent driver of gut dysbiosis and long‐term cognitive deficits among undernourished communities (Brown et al., 2015; Guerrant et al., 2013; Kau et al., 2015; Vonaesch et al., 2018). After 4 weeks on the malnourished diet, MAL and MAL‐BG mice exhibit modest growth faltering and reduced tail length, a proxy for murine stunting (Figure S1a).

Our lab previously demonstrated that *E. coli* and Bacteroidales exposures fail to trigger growth deficits and gut dysbiosis in the absence of malnutrition (CON‐BG model), likely due to lack of robust *E. coli*/Bacteroidales colonization in mice fed a healthy diet (Brown et al., 2015); growth and fecal Enterobacteriaceae relative abundance measurements repeated in Figure S1b,c. In contrast, MAL‐BG mice exhibit striking gut dysbiosis, impaired gastrointestinal host–microbe interactions, intestinal barrier deficits, and altered gut metabolomes (Brown et al., 2015; Huus et al., 2020). As *E. coli*/Bacteroidales bacterial gavage fails to robustly colonize healthy control mice, we were not able to appropriately assess whether these fecal exposures influenced brain and behavior alterations in the absence of dietary malnutrition. Consequently, we focused further analyses on malnutrition models.

We next assessed whether the MAL and MAL‐BG models exhibit behavioral and/or cognitive impairments, neurologic consequences of early‐life malnutrition and co‐occurring fecal‐oral contamination (Black et al., 2013; Investigators, 2018). CON mice provided a healthy control for murine tests.

The OFT measures locomotion and exploration (Gould et al., 2009). Increased aversion to the central open field zone (OFZ) connotes anxiety‐like behavior in rodents (Gould et al., 2009). MAL‐BG mice spent more time within the OFZ (Figure 1b,c), displaying increased exploration compared to either CON or MAL groups (F_2,57_ = 6. 878, *p* = .0021). Immobility (resting) within the OFZ, but not total OFT immobility, increased among MAL‐BG mice, supporting an absence of OFZ‐induced anxiety (Figure  S2a). These results were not shaped by gross locomotion deficits as total distance traveled in the OFT was comparable across all groups (Figure S2b). In addition, CON, MAL, and MAL‐BG mice displayed comparable behavior within the light–dark box, an established murine anxiety test (Bourin & Hascoët, 2003), further supporting altered exploration, rather than anxiety‐like activity (Figure S2c).

MAL‐BG mice also exhibited distinct exploratory patterns during the NORT. NORT not only measures novelty exploration, but also assessed short‐term memory performance (Leger et al., 2013). During a brief familiarization period, individual mice freely explored an arena with two identical objects. After familiarization, one object was replaced with a similarly sized, but distinctly “novel” object (Figure S2d). Individual mice were returned to the disinfected arena after several hours for the recall period. As rodents typically exhibit novelty preference, decreased exploration of the novel item indicates impaired novel object recognition. All groups exhibited novelty preference (novel:old exploration ratio > 1; *t*_47_ = 6.779, *p* < .0001) and comparable total exploration time (Figure S2d,e). Compared to CON and MAL counterparts, MAL‐BG mice exhibited a modest, but not significant, increase in novel object interaction (Figure S2d). Results from both the OFT and NORT suggest altered exploratory behavior in MAL‐BG mice.

We further assessed spatial memory and cognition via the Morris water maze test (MWMT), which measures spatial learning, reference memory, and cognitive flexibility (Vorhees & Williams, 2006). Mice underwent two training periods (acquisition, reversal) to learn the location of a hidden platform (Figure 1d and Figure S2f). Average swim speeds were recorded during a 30 s free swim (no platform) 24 h after each training period. As MAL and MAL‐BG mice displayed similar swimming capability to healthy controls (Figure S2g), MWMT results were not influenced by altered physicality.

We observed comparable reference memory and spatial learning during acquisition training (Figure 1d). We next probed learning within the context of cognitive flexibility, placing the hidden platform within the opposite pool quadrant (reversal learning). Upon reversal, MAL and MAL‐BG escape latencies (time to platform) increased, indicative of impaired learning flexibility (Vorhees & Williams, 2006). Learning deficits persisted, even broadened, across the reversal period (Day 1_R_: F_2,45_ = 2. 836, *p* = .0692; Day 2_R_: F_2,42_ = 5.205, *p* = .0096). Accidental platform discovery did not drive these findings, as reversal escape latencies during the initial trial were comparable across groups (Figure S2h). CON mice rapidly learned the new location of the hidden platform, while malnourished mice persistently honed to the prior platform location, indicative of impaired memory extinction. By the final training day (Day 2_R_), both CON and MAL mice significantly eliminated prior platform entries (CON: paired t‐test = 3.753, *p* = .0024; MAL: paired t‐test = 4.250, *p* = .0007). Indeed, over half of CON mice never entered the prior platform area and these mice rapidly located the hidden platform (Figure 1f and Figure S1i). In contrast, MAL‐BG mice did not exhibit significant memory extinction (Figure 1e,f and Figure S1i), as measured by prior platform entry from Day 1_R_ to Day 2_R_ (MAL‐BG: paired t‐test = 1.237, *p* = 0 .2381), exhibiting marked cognitive inflexibility (Mills et al., 2014).

Cognitive processes and learning flexibility require hippocampal synaptic plasticity and appropriate microglial function (Galloway et al., 2019; Mills et al., 2014; Vorhees & Williams, 2006; Wu et al., 2015). Recently, Wang et al., 2020 demonstrated that hippocampal microglia shape synaptic plasticity via phagocytic‐dependent synaptic pruning (Wang et al., 2020). As fecal microbes and/or diet also influence microglia (Erny et al., 2015; Madore et al., 2020), we next characterized hippocampal microglial morphology and transcriptional profile.

### 3.2 Diet and gut microbes influence microglial morphology and function

Microglia modulate brain plasticity and maintain CNS homeostasis. Indeed, alterations in microglial morphology and phagocytic capacity inform memory plasticity (Wang et al., 2020). Like peripheral macrophages, microglia are highly responsive to environmental changes within the brain, regulating and responding to inflammatory and metabolic shifts, as well as gut microbial alterations (Erny et al., 2015; Wu et al., 2015; York, Bernier, & MacVicar, 2018). Mature microglia exhibit a range of phenotypes from quiescent “resting” (ramified morphology with extended processes) to “activated” (amoeboid morphology with retracted processes). Consequently, morphology provides a valuable indicator for microglial activation and broad functionality (Karperien et al., 2013). While aberrant microglia contribute to various neuropathologies, undernourished microglia remain largely unstudied.

**FIGURE 2:**
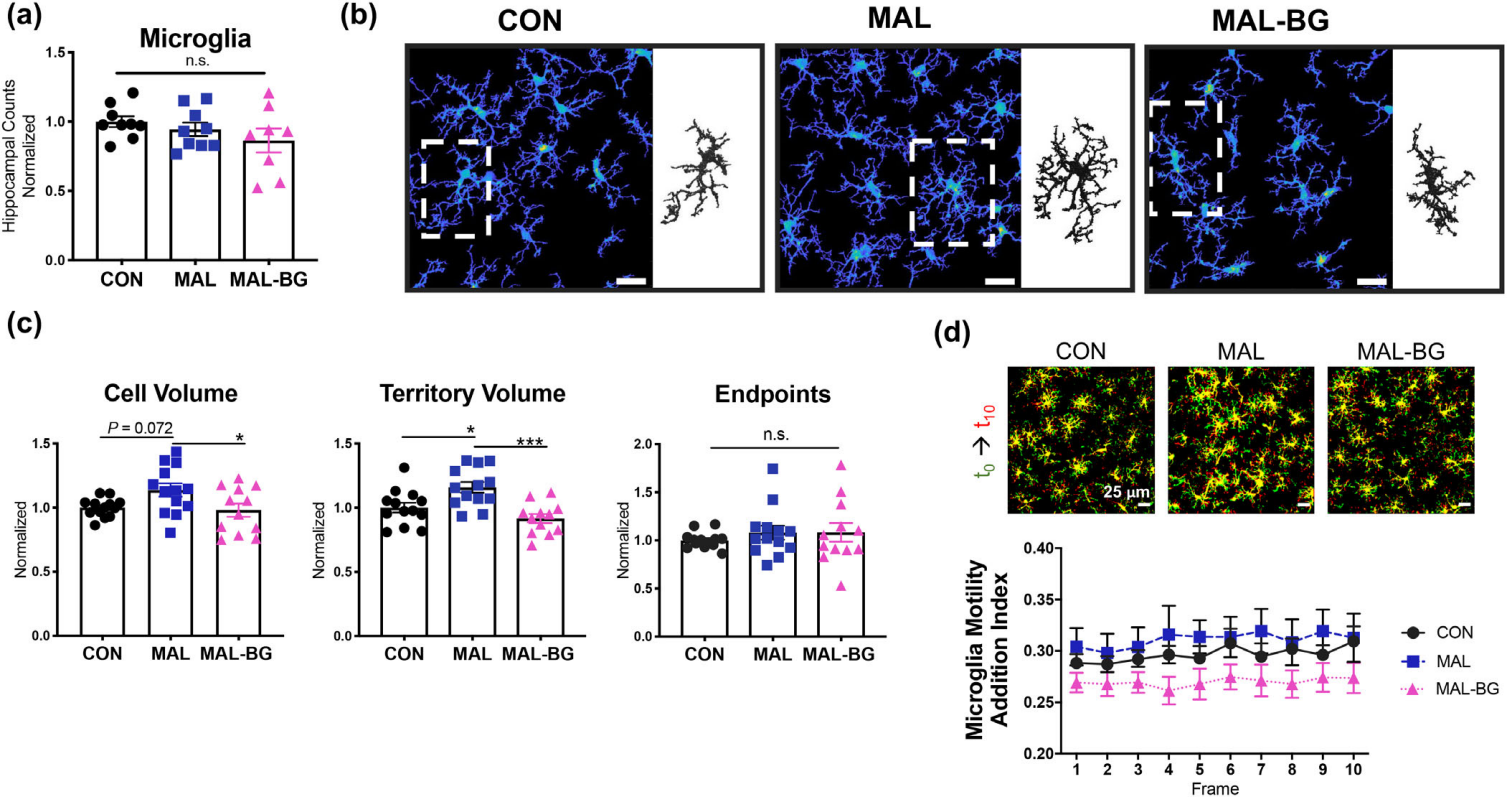
Gut microbes modulate microglial morphology, but not motility, during malnutrition. (a) Microglia cells counts within the CA1 hippocampal region of CX3CR1^+/EGFP^, data pooled from three experiments with data normalized to the CON group of each experiment, *n* = 9 CON, 9 MAL, and 9 MAL‐BG. (b) CA1 hippocampal images with representative CON, MAL, and MAL‐BG microglia (inset). (c) Microglial morphology was quantified from four separate experiments using 3DMorph software and normalized to the CON group of each experiment, *n* = 13 CON, 13 MAL, and 12 MAL‐BG. (d) To assess whether morphological alterations affect motility, we examined microglial process additions and retractions over 10 min. Representative images from motility assays: yellow = static, red = process addition, green = process retraction. We observed no striking differences in CON, MAL, and MAL‐BG microglial motility, as quantified by process addition or retraction motility indices, *n* = 9/group (see also Figure S3b). Results from a/d are a subset of mice from data described in c. Graphs indicate mean and SEM with statistical significance determined by one‐way ANOVA with post hoc Tukey's test; n.s., non‐significant

Using two‐photon microscopy, we assessed microglial morphology and motility within the hippocampus (CA1 region) of CX3CR1^+/EGFP^ mice on a C57BL/6J background (Jung et al., 2000). To assess microglial morphology, we utilized 3DMorph (York, LeDue, et al., 2018), which provided semi‐automatic, multidimensional measurements across four independent experiments. We observed comparable numbers of hippocampal microglia independent of diet and microbial exposure (Figure 2a). Microglia from CON mice display typical ramified (“resting”) morphology. In contrast, MAL and MAL‐BG microglia exhibit divergent phenotypes compared to healthy controls with larger and smaller cell volumes, respectively (Figure 2b,c). Compared to MAL mice, we observed decreased microglial cell volume and territory (total surveillance area) volume (F_2,35_ = 3.942, *p* = .0286 for cell volume; F_2,35_ = 10.06, *p* = .0004 for territorial volume) within the MAL‐BG model. These alterations did not significantly influence process number (endpoints) or process complexity (branch points), see Figure 2c and Figure S3a. Collectively, these results suggest that microglial morphology is shaped by both dietary malnutrition and by the combination of malnutrition plus fecal microbial exposures. Altered volume/territorial volume, as observed in malnourished microglia, affects the area of brain continually surveyed by microglia. These morphological alterations were of sufficient interest to further characterize microglia, specifically microglial motility and transcriptional profile.

CON, MAL, and MAL‐BG mice exhibit comparable motility as measured by process additions and retractions across time (Figure 2d and Figure S3b). We then assessed microglial surveillance in the context of acute hippocampal insult. Damaged and apoptotic cells trigger rapid microglial responses, with microglial processes cordoning off injured tissue (Davalos et al., 2005). Focused two‐photon laser scanning induced precise lesions in ex vivo hippocampal slices. Microglial response strength (percent of responding processes to the lesion) and response speed were comparable across CON, MAL, and MAL‐BG mice. Indeed, ~90% of processes from neighboring microglia surrounded injured tissue within 10 min of lesion induction (Figure S3c,d; representative videos available within Supplemental File 2). After examining microglial morphology and motility, we explored putative functional shifts through transcriptional profiling.

**FIGURE 3:**
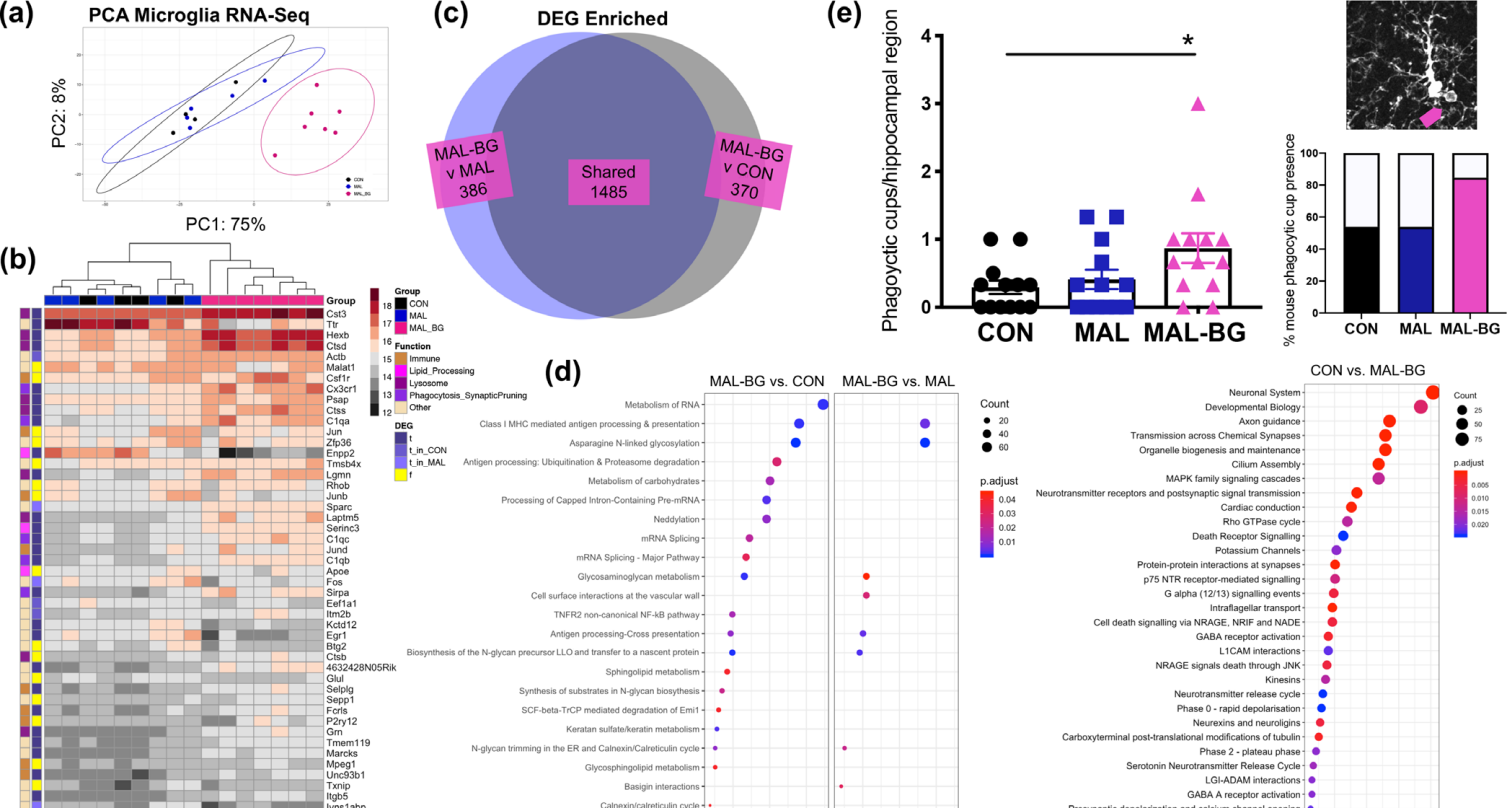
MAL‐BG microglia exhibit altered gene profile and increased phagocytic structures. (a) PCA of transformed gene counts from RNA‐Seq data of microglial‐enriched samples (CD11b+), *n* = 4 CON, 5 MAL, 7 MAL‐BG. (b) Heatmap of transformed gene counts with DEG (*Padj* < 0.05, FC > |1.5|) and gene function data provided. DESeq2 identified the top 50 most abundant microglial genes; MAL‐BG samples exhibit low intrasample variability (Euclidean distance). DEGs in blue and non‐DEGs in yellow (t = true, f = false). Genes were searched against the NCBI and GeneCards® databases to determine function. (c) Venn diagram reporting overexpressed MAL‐BG DEGs compared to CON (dark gray) and MAL (blue) samples. (d) Pathway enrichment analyses conducted with ReactomePA (DEGs: *Padj* < 0.05, FC > 1.5); MAL‐BG versus CON (left) or MAL (right). Far right panel: CON versus MAL‐BG pathway enrichment analysis (top 30 pathways); network visualizations reported in Figure S4d. (e) Data from four independent experiments, each symbol represents average microglial cup number per hippocampal region, *n* = 13/group. Representative microglial phagocytic cup (pink arrow, top right). Percent of mice that exhibited microglial phagocytic feature/s, phagocytic cups counted from hippocampal slices by a blinded observer (bottom right). Results in a–d are from the same experiment. Bar graph indicates mean and SEM with statistical significance determined by one‐way ANOVA with post hoc Tukey's test

To examine transcriptional alterations, we conducted RNA‐Seq following microglial enrichment (CD11b+ population) from CON, MAL, and MAL‐BG whole brains (~90% microglia, flow gating provided in Figure S4a). Count transformation and identification of differentially expressed genes (DEGs) from RNA‐Seq data were determined by DESeq2 (Love et al., 2014). PCA of transformed mRNA gene counts revealed a striking shift in the transcriptional profile of MAL‐BG microglia (Figure 3a). After filtering low‐gene counts, 4685 genes were differentially expressed between MAL‐BG and CON samples, while 4454 genes were differentially expressed between MAL‐BG and MAL samples (*Padj* < 0.05, FC > |1.5|). Remarkably, no DEGs were observed between CON and MAL samples. These results suggest that a combination of malnutrition and specific microbial exposure leads to altered microglial gene expression as malnutrition alone was not sufficient to induce significant transcriptomic changes in MAL microglia.

To further explore transcriptional alterations, we identified the most abundant genes present across samples. MAL‐BG mice exhibit a distinct microglial transcriptional profile (Euclidean clustering). NIH (NCBI) and Weizmann Institute (GeneCards®) gene databases provided broad gene function (Figure 3b). As anticipated, highly expressed non‐DEGs serve essential cellular activities, including key transcriptional regulators (*Malat1* and *Btg2*). The function of many highly expressed DEGs were broadly categorized into (1) lipid metabolism (e.g., *Enpp2*) and degradation pathways, including (2) lysosomal processing, notably cathepsin proteases (e.g., *Ctsd* and *Ctss*) and (3) phagocytosis regulation and/or synaptic pruning (e.g., *Sirpa* and complement pathway genes including *C1qa*, *C1qc*, *C1qb*).

Similar transcriptional shifts were supported by flow cytometry, qPCR, and biological pathway analyses (Figure S4b–d). Over 1800 DEGs were overexpressed in MAL‐BG samples (*Padj* < 0.05, FC > 1.5), compared to CON or MAL mice (Figure 3c). ReactomePA (hypergeometric model) (Yu & He, 2016) identified biological pathways enriched in healthy and malnourished microglia (Figure 3d and Figure S4d). Enrichment profiles were comparable between MAL‐BG versus CON and MAL‐BG versus MAL conditions, with more pathways identified in the MAL‐BG versus CON pathway analysis (Figure 3d, left). Enriched CON versus MAL‐BG pathways identified multiple homeostatic processes; notably neurotransmitter signaling, MAPK signaling cascades, and axon/synapse regulation. MAL‐BG microglia display altered lipid and carbohydrate metabolic pathways. Notably, major histocompatibility (MHC) class I and antigen‐processing pathways were highly enriched in MAL‐BG samples (Figure 3d and Figure S4d), processes linked to phagocytosis and increased degradation events (Fu et al., 2014; Münz, 2010). To validate a phagocytic MAL‐BG profile, we counted the number of large phagocytic cups—actin rich, lasso‐like structures formed during microglial envelopment/engulfment (Persaud‐Sawin et al., 2009). A moderate increase of phagocytic structures was present within an independent cohort. MAL‐BG mice exhibited nearly 3× more phagocytic cups compared to healthy controls (Figure 3e, left). Approximately half of hippocampal slices from CON or MAL featured phagocytic cups compared to >80% within the MAL‐BG model (Figure 3e, right).

Complement‐dependent microglial phagocytosis shapes diverse brain functions, from continued modulation of neural plasticity and memory via synaptic pruning, to engulfment of noxious stimuli during neuroimmune responses (Wang et al., 2020; Wu et al., 2015; York, Bernier, & MacVicar, 2018). To identify the scope of MAL‐BG alterations and examine how fecal‐oral contamination contributes to microglial alterations and putative phagocytic function, we assessed key gut microbiota‐brain pathways, namely inflammation, barrier integrity, and neurometabolism (Bauer et al., 2016, 2019).

### 3.3 Inflammation and BBB integrity unaltered in MAL‐BG model

Co‐occurring malnutrition and fecal‐oral contamination often present with systemic comorbidities including immune dysregulation and low‐grade inflammation, processes linked to gut dysbiosis (Brown et al., 2015; Crane et al., 2015; Di Giovanni et al., 2016). To assess whether MAL and MAL‐BG microglia respond to neuroinflammation, we examined CNS and peripheral (sera) inflammation. MAL and MAL‐BG mice exhibited low levels of TNF‐α or IL‐6 proinflammatory cytokines, comparable to CON counterparts (Figure S5a,b).

To specifically address microglial‐mediated inflammatory responses, we measured expression of key immune receptors by flow cytometry (CD11b^high^/F480^high^/CD45^low^ population; Figure S5c). CD86, MHC II and toll‐like receptor (TLR) 4 contribute to immunoregulation and pathogen responses and microglial upregulation of these receptors has been established during neuroinflammatory conditions (Schetters et al., 2018; Wang et al., 2019). The frequency and geometric mean fluorescence intensity (gMFI) of CD86, MHC II, and TLR 4 were comparable across dietary conditions (Figure S5d). Collectively, these findings suggest that the morphological and transcriptional profile observed in MAL‐BG microglia is distinct from classical features of inflammatory microglial activation.

We previously reported that fecal‐oral contamination influences small intestinal permeability in malnourished mice (Brown et al., 2015), (see also Figure S5e). In addition to regulating the enteric barrier, gut microbes have been linked to the development and maintenance of the CNS analog—the BBB (Braniste et al., 2014). BBB permeability was measured by IgG immunostaining and tetramethylrhodamine biocytin (biocytin‐TMR) permeability across the neural vasculature. All groups exhibited low levels of interstitial IgG, indicative of BBB integrity (Readnower et al., 2010) (Figure S5f). As further validation, we measured biocytin intensity within the brain following biocytin‐TMR tail vein injection. CNS endothelial cells lack vitamin transporter *Slc5a6* required for expected biocytin transport, though significant BBB deficits enable biocytin‐TMR CNS distribution (Knowland et al., 2014). We observed no difference in biocytin‐TMR intensity throughout cortical tissue in CON, MAL, and MAL‐BG mice (Figure S5g–i). We note that BBB integrity was measured at our standard endpoint (14 day following bacterial gavage). These findings do not exclude the possibility of transitory BBB deficits at earlier timepoints. Moreover, these methods may not capture more subtle BBB deficits. Markers of transient BBB disruption and/or pathogen‐induced proinflammatory cytokine elevation, notably peripheral immune cell infiltration, were not observed in CON, MAL, and MAL‐BG brains (see macrophage population, Figure S4a) (Schetters et al., 2018). Collectively, these results support broad BBB maintenance in the malnourished models.

We then assessed neurometabolism, specifically targeting the hippocampus, a critical region for cognitive function and spatial memory and the site of microglial morphology analyses (Mills et al., 2014). Untargeted metabolomic analyses were conducted on single hippocampi from CON, MAL, and MAL‐BG mice (*n* = 5) by RP‐UPLC‐FTMS. This method identified and relatively quantified >6300 unique metabolite features with 25 differentially abundant hits (one‐way ANOVA Fischer's LSD, *Padj* < 0.05), far fewer compared to the previously reported small intestine metabolome (Brown et al., 2015), likely highlighting CNS resilience against malnutrition. Indeed, the BBB and extensive energy requirements contribute to a distinct, and energetically resilient, metabolomic brain profile (Camandola & Mattson, 2017; Qi et al., 2020).

**FIGURE 4:**
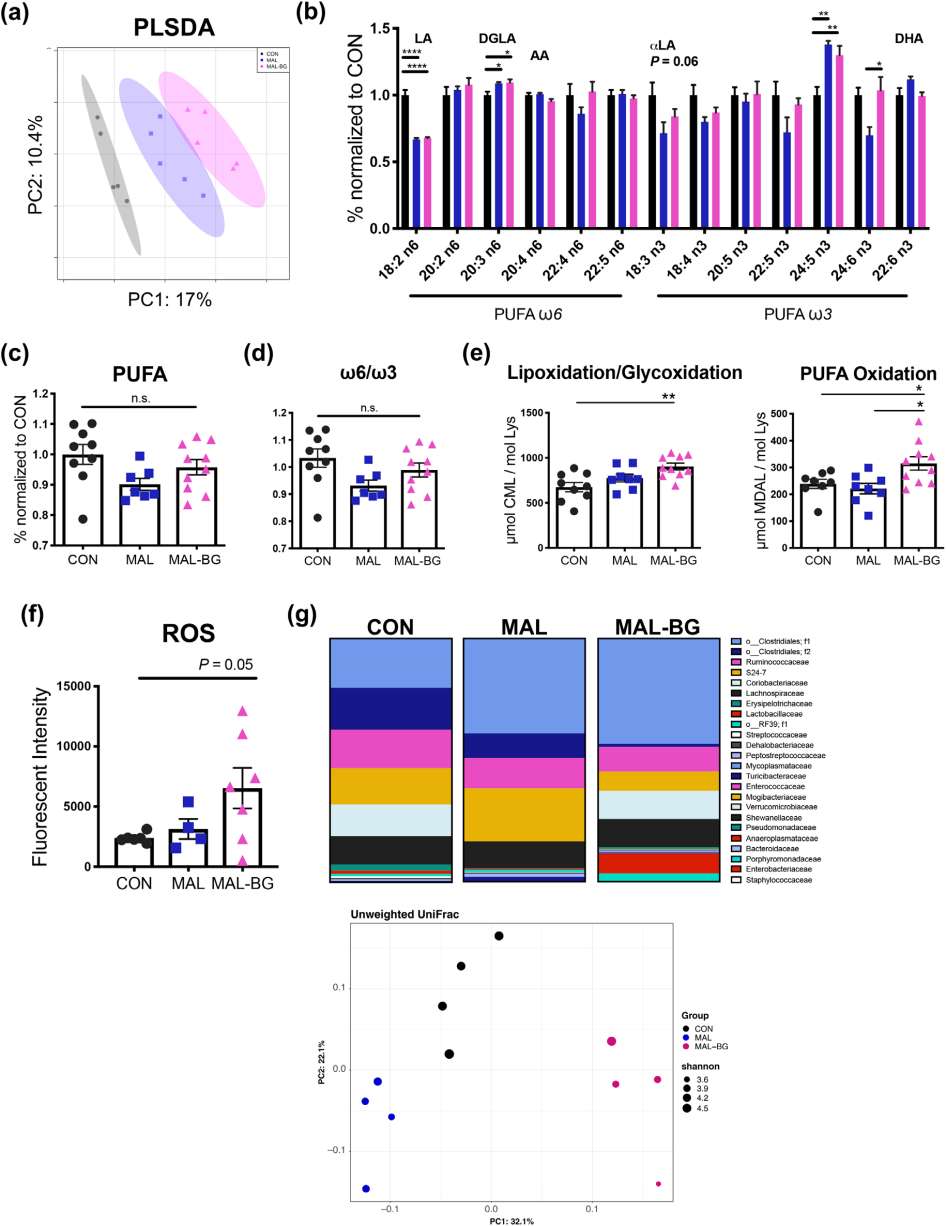
Specific gut microbes promote systemic oxidative stress in MAL‐BG model. (a) PLSDA of untargeted hippocampal metabolomics, data from the negative ion channel, *n* = 5/group (see also Figure S6a). (b) The relative abundance of PUFAs within the murine cortex normalized to controls. Data revealed alterations in ω6 and ω3 metabolism, major PUFAs labeled: LA, linoleic acid, DGLA, dihomo‐γ‐linolenic acid, AA, arachidonic acid, αLA, α‐linoleic acid, and DHA, docosahexaenoic acid (*n* = 8–9 CON, 7 MAL, 10 MAL‐BG). (c) Relative abundance of cortical PUFA levels normalized to controls. (d) ω6/ω3 ratio from cortical CON, MAL, and MAL‐BG tissue, *n* = 7–10/group. (e) CML and MDAL levels from murine cortical tissue. Data normalized to tissue mol lysine. (f) Cellular ROS levels within ex vivo intestinal epithelial cells from the CON, MAL, and MAL‐BG small intestine, fluorescent intensity was measured via plate reader following 30 min treatment with CellROX® (15 μM final concentration). (g) The average relative abundance of the fecal microbiota (*n* = 4) by family classification determined by the 16S rRNA gene (above). Unweighted UniFrac PCA and α‐diversity (Shannon index) of the CON, MAL, and MAL‐BG microbiota (below), see also Figure S7a. Microbiome analyses conducted using QIIME2 (v. 2018.2). Fatty acid profiling and CNS oxidative stress measures are from the same experiments. Bar graphs indicate mean and SEM with statistical significance determined by one‐way ANOVA with post hoc Tukey's test (fatty acid profiling, oxidative stress) or Dunnett's test (ROS measurement). PLSDA, partial least squares discriminant analysis; PUFA, polyunsaturated fatty acid; n.s., non‐significant [Correction added on January 29, 2022, after first online publication: Figure 4 legend has been updated.]

PLSDA and unsupervised PCA revealed moderate shifts in the hippocampal metabolome (Figure 4a and Figure S6a). Differentially abundant *m/z* features were annotated against KEGG and METLIN databases. Ion mode, *m/z*, and putative annotations are reported in Figure S6b. These metabolites contributed to lipid signaling pathways, recalling both the microglial transcriptome (Figure 3b–d) and previously reported metabolic alterations within the malnourished small intestine (Brown et al., 2015). More specifically, MAL‐BG mice exhibited altered PUFA metabolism, validated by GC analyses of an independent cohort.

Compared to standard chow, the malnourished diet contains ~one third of essential PUFAs: linoleic acid (LA, 18:2 ω6) (Brown et al., 2015). Despite a modest increase in chow consumption (Figure S6c), malnourished mice maintain reduced dietary PUFA intake compared to healthy controls. While dietary‐dependent LA levels are reduced in malnourished brain tissues, total PUFA levels are comparable across healthy and malnourished models (Figure 4b,c). Levels of ω6 PUFAs derived from LA were unaltered (e.g., arachidonic acid [AA]) or even elevated (e.g., dihomo‐γ‐linolenic acid [DGLA]) in MAL and MAL‐BG brains (Figure 4b), supporting RP‐UPLC‐FTMS analyses (Figure S6b). Elevated ω6/ω3 PUFA ratio, an established marker of metabolic‐induced inflammation and oxidative stress (Augusto et al., 2017; Katrenčíková et al., 2021; Maes et al., 2008), was not observed in MAL and MAL‐BG brains (Figure 4d).

### 3.4 Specific gut microbes exacerbate malnutrition‐induced oxidative stress

Comprised of multiple unsaturated double bonds, PUFAs are highly susceptible to oxidation (Pamplona et al., 2005). Notably, lipoxidation of ω6 PUFAs produces inflammatory and oxidative mediators linked to neurocognitive pathologies (McNamara & Almeida, 2019; Melo et al., 2019; Pamplona et al., 2021). As oxidative stress has been linked with microglial dysfunction (Brown & Neher, 2014; Padurariu et al., 2010; Pamplona et al., 2005), we assessed amino acid (protein) oxidation, glycoxidation, and lipoxidation (Figure 4e and Figure S6d). Malnutrition alone was sufficient to promote CNS oxidative stress (Figure S6d), increasing HAVA ([2H5] 5‐hydroxy‐2‐aminovaleric acid) and CEL (Nε‐[carboxyethyl]‐lysine) levels, biomarkers of protein oxidation and glycoxidative stress, respectively (F_2,24_ = 9.494, *p* = .0009; F_2,24_ = 9.576, *p* = .0009). The combination of malnutrition plus fecal microbial exposures significantly exacerbated lipoxidative markers. MAL‐BG brains displayed elevated CML (Nε‐[carboxymethyl]‐lysine) (lipoxidation/glycoxidation marker) and MDAL (Nε‐[malondialdehyde]‐lysine) (PUFA‐dependent lipid peroxidation) levels (F_2,24_ = 6.954, *p* = .0042 for CML; F_2,23_ = 5.618, *p* = .0103 for MDAL; Figure 4e (Pamplona et al., 2005). We then searched the microglial transcriptome for genes within lipoxidative pathways, including NADPH oxidase, a major source of microglial‐generated ROS (Bouzidi & Doussiere, 2004; Wang et al., 2018; York, Bernier, & MacVicar, 2018). Microglial genes linked to oxidation and NADPH oxidase activation (e.g., *S100a8* and *S100a9*) were elevated within the MAL‐BG model (Supplemental File 3a). While elevated NADPH oxidase genes may reflect inflammatory responses (Wang et al., 2018), transcriptional alterations occurred in the absence of elevated TLR4/MHCII microglial expression and increased proinflammatory cytokine levels (TNF‐α and IL‐6; Figure S5a,d). These findings suggest that MAL‐BG microglia both respond and contribute to CNS oxidative pathways.

**FIGURE 5:**
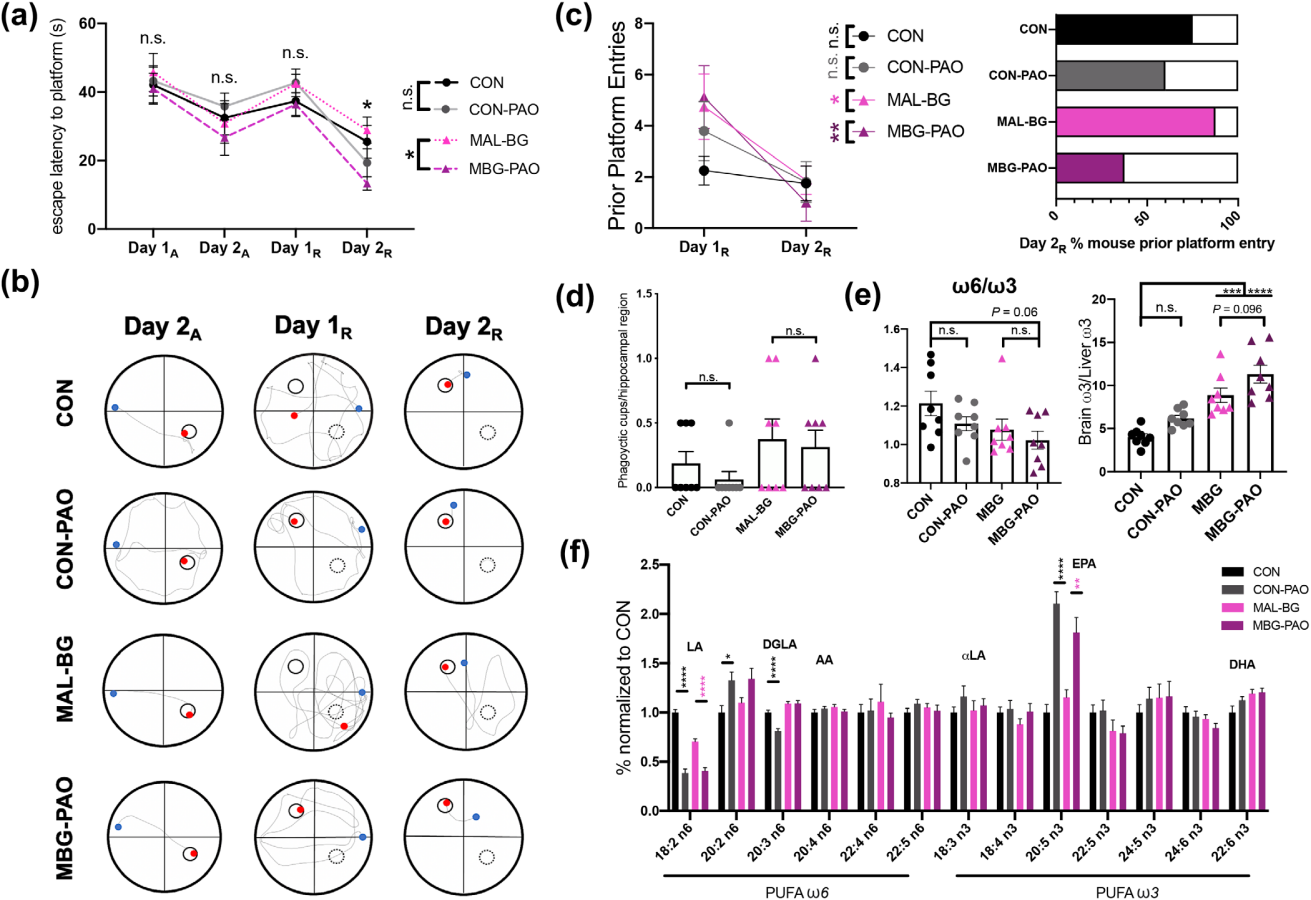
PAO dietary intervention largely mitigates cognitive MAL‐BG deficits. (a) Averaged escape latencies (time to hidden platform) of individual mice throughout acquisition (Day 1_A_, Day 2_A_) and reversal (Day 1_R_, Day 2_R_) learning. (b) Swim paths from the final Day 2_A_, Day 1_R_, and Day 2_R_ swim; same representative CON, CON‐PAO, MAL‐BG, or MBG‐PAO mouse across trials: solid circle = platform location, dotted circle = prior platform location, blue dot = start position, and red dot = final position. (c) Total prior platform entries for mice (mean = group average) from Day 1_R_ and Day2_R_, statistics determined by a two‐tailed, paired t‐test (left). Percent of mice in each group that entered prior platform on Day 2_R_ (right), *n* = 5–8/group. (d) Each symbol indicates average microglial phagocytic cup features within hippocampal images for each individual mouse. (e) ω6/ω3 ratio in murine cortical tissue (left) and measure of systemic ω3 shunting: ratio of brain/liver mol% ω3 (right). (f) The relative abundance of ω6 and ω3 PUFAs within the murine cortex normalized to controls: LA, linoleic acid, DGLA, dihomo‐γ‐linolenic acid, AA, arachidonic acid, αLA, α‐linoleic acid, EPA, eicosapentaenoic acid, and DHA, docosahexaenoic acid, *n* = 8/group for fatty acid analyses. Data in Figure 5 from same biological dataset. MWMT results were blindly conducted on ANY‐maze software. Graphs present mean with SEM with statistical significance determined by one‐way ANOVA with post hoc Tukey's test Dunnett's test (MWMT graph a) or one‐way ANOVA with post hoc Tukey's test (microglia, fatty acid analyses): MWMT, Morris water maze test; n.s., non‐significant

Aberrant oxidation has long been considered both a driver and consequence of early‐life malnutrition (Khaled, 1994; Manary et al., 2000; Preidis et al., 2014). As gut microbiota dysbiosis, including altered *E. coli* abundance, have been linked to systemic oxidative stress (Cani et al., 2008; Kostic et al., 2014; Qiao et al., 2013), we hypothesized that ROS may originate at the interface of the gut microbiota and host. Volatile ROS trigger production of reactive aldehydes, such as MDAL, initiating a vicious cycle of continued lipid peroxidation (Barrera et al., 2016; Kwiecien et al., 2014; Pamplona et al., 2005). To assess intestinal oxidative stress, we measured ROS in ex vivo epithelial cells harvested from the CON, MAL, or MAL‐BG small intestine. Following *E. coli*/Bacteroidales exposure, the average ROS levels roughly doubled within the MAL‐BG gut (Figure 4f).

As fecal‐oral contamination promoted intestinal dysbiosis, we also assessed the malnourished gut microbiota. PCA of unweighted UniFrac distances generated from 16S rRNA sequencing revealed marked shifts in the MAL and MAL‐BG fecal microbiota, with samples clustering by bacterial exposure, then diet (Figure 4g and Figure S7a). Notably, the MAL‐BG microbiota exhibited a significant increase in the relative abundance of select bacterial gavage members, verifying robust microbial exposure (Figure S7b). To further explore how fecal commensals modulate the malnourished microbiome, we assessed predictive functionality of the MAL and MAL‐BG microbiota with PICRUSt. The majority of top differentially abundant pathways, following FDR correction, included amino acid metabolism and nucleic acid biosynthesis/scavenging pathways (Figure S7c and Supplemental File 3b pathways responsive to diet and oxidative strain (Huang et al., 2017; Ishii et al., 2018; Mar et al., 2016; Ni et al., 2019).

Long‐term consequences of malnutrition include brain and behavioral deficits, linked to impaired gut‐brain interactions (Black et al., 2013; Guerrant et al., 2013; Tofail et al., 2018). MAL‐BG results suggest that fecal‐oral contamination exacerbates impaired PUFA metabolism and lipoxidative stress during malnutrition. Indeed, acute undernutrition promotes systemic fatty acid oxidation within pediatric, malnourished cohorts at risk for intestinal infection (Bartz et al., 2014; Semba et al., 2017). We hypothesize that abnormal PUFA processing and subsequent lipoxidative stress likely contribute to aberrant microglial activity, ultimately affecting behavior and cognition.

### 3.5 Dietary intervention shapes MAL‐BG brain and PUFA profiles

To assess the impact of dietary intervention, we developed PAO (ω3 PUFA‐rich, antioxidant vitamin) fortification. PAO composition was based on published, antioxidant‐associated murine diets (McPherson et al., 2016; Vargas‐Robles et al., 2015), as well as recent dietary recommendations for pediatric undernutrition (Jones et al., 2015; Mehta et al., 2021; van der Merwe et al., 2013). The CON‐PAO diet and MAL‐PAO diet complement macronutrient and caloric content of the standard and malnourished diet, respectively.

PAO diets were enriched with ω3 PUFAs and antioxidant vitamin C/E (ascorbic acid phosphate/vitamin E acetate). Diets with high ω6/ω3 PUFA content have been repeatedly linked to aberrant inflammation and oxidative stress (Augusto et al., 2017; McNamara & Almeida, 2019; Padurariu et al., 2010; Pamplona et al., 2005). While CON, MAL, and MAL‐BG mice exhibit comparable ω6/ω3 ratios (Figure 4d), malnutrition altered ω3 and ω6 PUFA profiles (Figure 4b). An ω3‐enriched diet likely reduces ω6 lipoxidation events (Heshmati et al., 2019), while low ω6/ω3 intake mitigated CNS oxidative stress in malnourished rat models (Augusto et al., 2017). Intriguingly, recent work linked dietary ω3 PUFAs and microglial phagocytic function. Maternal ω3 PUFA deficits activated aberrant microglial phagocytosis in offspring, contributing to poor cognitive outcomes (Madore et al., 2020).

To reduce ω6/ω3 intake, PAO diets swapped fatty acid sources from ω6‐rich soybean oil (standard diet: 630 kcal%; malnourished diet: 210 kcal%) to ω3‐rich menhaden (fish) oil with equivalent kcal%. In addition to PUFA‐dependent shifts, the PAO diet was supplemented with ~4× higher concentration of vitamin C/E, potent antioxidants frequently incorporated within antioxidant murine diets (McPherson et al., 2016; Rebrin et al., 2005; Vargas‐Robles et al., 2015). Prior metabolite analyses informed levels of PAO vitamin supplementation (Brown et al., 2015).While non‐PAO diets (standard, malnourished) contain equivalent micronutrient content, our lab previously demonstrated significant reduction of intestinal vitamins within malnourished mice. Full dietary composition reported in Supplemental File 1.

We chose to assess the impact of PAO fortification in the MAL‐BG model in C57BL/6 mice, as these mice exhibit significant cognitive deficits, a tragic consequence of chronic undernutrition (Black et al., 2013). CON mice provided a healthy control. For concise naming, malnourished mice fed a PAO diet are subsequently labeled MBG‐PAO (**M**AL‐**BG** + PAO diet). Here, we report analyses conducted on CON, CON‐PAO, MAL‐BG, and MBG‐PAO models, model set‐up reported in Figure S8a.

Unexpectedly, PAO intervention failed to improve malnutrition‐induced growth deficits and even promoted stunting features. MBG‐PAO mice exhibited modest, but not significant, reduction in weight and tail length compared to MAL‐BG counterparts. In contrast, CON‐PAO mice displayed significant growth faltering (Figure S8b), likely driven by decreased chow intake (Figure S8c). Although dietary intervention failed to improve anthropometric features, PAO fortification mitigated cognitive deficits of malnutrition as assessed via MWMT.

PAO cognitive testing revealed similar trends as previous MWMT analyses. CON and MAL‐BG mice displayed comparable learning capacity during MWMT acquisition training (Figure 5a). As before, we observed impaired escape latency (time to platform) following relocation of the hidden pool platform (reversal training), Day 2_R_: F_3_,_25_ = 3.546, *p* = 0.0289. PAO fortification benefited MWMT cognitive readouts, notably escape latency and prior platform entry (Figure 5a‐c). CON‐PAO subjects exhibited modest improvement in escape latency compared to CON counterparts during reversal training. MBG‐PAO mice, however, located the hidden platform significantly faster than MAL‐BG subjects (Day 2_R_ average escape latency [s] and SD: CON = 25.53 ± 13.39, CON‐PAO = 19.37 ± 9.18, MBG = 28.87 ± 10.79, and MBG‐PAO = 13.32 ± 5.50); Figure 5a. These changes were not linked to growth‐dependent swimming impairment as average swim speeds remained consistent throughout MWM testing (Figure S8d). Once again, we observed that healthy mice largely eliminate prior platform entry, a measure of leaning plasticity (Figure 5c and Figure S8e). Unlike prior MWM testing, healthy controls did not significantly reduce prior platform entry from Day 1_R_ to Day 2_R_, due to near elimination of platform entries on Day 1_R_ within this experimental round. The malnourished PAO diet however, led to striking reduction of prior platform entry (MAL‐BG: paired t‐test = 2.631, *p* = 0.034; MBG‐PAO: paired t‐test = 3.667, *p* = 0.008). Moreover, only 37.5% (3/8 mice) of MBG‐PAO subjects entered the prior platform throughout four trials on Day 2_R_, compared to 87.5% (7/8 mice) MAL‐BG subjects (Figure 5c and S8e), highlighting improved spatial memory and learning plasticity linked to malnourished PAO intervention.

As appropriate learning involves microglia phagocytic processes (Wang et al., 2020; Wu et al., 2015), we next assessed microglia morphology. We observed no significant differences in healthy and malnourished C57BL/6 microglial cell volume, territory volume, or endpoints (Figure S8f), comparable with CX3CR1^+/EGFP^ microglial analyses (Figure 2c). As MAL‐BG microglia exhibit a phagocytic transcriptional profile and increased phagocytic features (Figure 3b–e), we also counted phagocytic cups within the PAO study. While MAL‐BG mice exhibited greater phagocytic cup features, these changes did not reach statistical significance. PAO intervention also failed to significantly alter presence of phagocytic features in healthy and malnourished mice (Figure 5d and Figure S8g). Whether comparable phagocytic features in PAO and non‐PAO models reflected a difference in mouse genotype and/or lack of robust intervention outcomes remains unclear. To investigate putative PAO‐dependent features, we profiled PUFA levels and oxidative markers.

PAO murine brains displayed comparable total PUFA levels with their dietary counterparts (Figure S8h). PAO intervention resulted in altered ω6/ω3 PUFA ratios, modestly reduced in the MBG‐PAO brain (Figure 5e left). As expected, PAO diets reduced LA (18:2 ω6), an essential PUFA of soybean‐based diets. In contrast, CON‐PAO and MBG‐PAO mice exhibited elevated eicosapentaenoic fatty acid (EPA, 20:5 ω3), an ω3 PUFA fish oil. To further corroborate PAO fortification, we quantified ω6/ω3 PUFA ratios within another fatty organ highly sensitive to dietary shifts—the liver (Bauer et al., 2020; Naudí et al., 2013). As expected. PAO‐fed mice displayed significant reduction in liver ω6/ω3 PUFA ratios compared to healthy and malnourished counterparts (Figure S8i). Moreover, brain ω3/liver ω3 ratios were significantly elevated in a step‐wise manner within MAL‐BG and MBG‐PAO mice suggesting systemic shunting of ω3 fatty acids in order to maintain appropriate ω3 PUFA content within the undernourished brain (see Figure 5e right). Despite altered PUFA profiles, CNS oxidative stress markers remained surprisingly consistent across CON, CON‐PAO, MAL‐BG, and MBG‐PAO mice (Figure S8j). These findings indicate that PAO fortification shapes systemic PUFA profiles, but remains insufficient to significantly reduce CNS lipoxidative stress markers.

In summary, PAO dietary intervention largely mitigated cognitive deficits in malnourished mice and aberrant CNS PUFA profiles. While cognitive outcomes occurred independently from significant shifts in microglial phagocytic features or decreased lipoxidative markers, PAO results do not exclude a putative role for oxidative‐dependent pathology. Neither do our collective findings preclude alternative mechanisms contributing to neurocognitive features within undernourished pediatric communities—including BBB disruption, neuroinflammation, and/or external stressors. Rather this work highlights gut‐glia interactions shaped by diet and commensal bacteria.

## 4 DISCUSSION

Despite intervention efforts, global malnutrition is expected to increase in response to interdependent disruptions from COVID‐19 and climate change (Littlejohn & Finlay, 2021; Osendarp et al., 2021; Phalkey et al., 2015). Many undernourished communities will experience lack of appropriate sanitation and chronic fecal‐oral contamination (Black et al., 2013; Humphrey, 2009). The pathways and pathologies informing malnourished gut‐brain interactions remain greatly unexplored. Here, we report a model of malnutrition that reflects prevalent dietary (i.e., fat/protein deficiency) and environmental (fecal‐oral contamination) conditions (Black et al., 2013; Guerrant et al., 2013; Littlejohn & Finlay, 2021). In addition to growth stunting and gut dysbiosis, MAL‐BG mice displayed cognitive deficits, notably impaired learning plasticity in the MWMT. These changes were accompanied by microglial and metabolomic shifts, distinct from both healthy (CON) and malnourished‐only (MAL) mice. Notably, the MAL‐BG microglial transcriptome contained increased expression of complement genes linked to phagocytic processes (e.g., C1qa) and hippocampal MAL‐BG microglia presented with increased phagocytic features.

These findings support a surge of ongoing research highlighting gut‐glia interactions.

Indeed, we specifically explored microglia due to (1) emerging work demonstrating the role of microglia on learning plasticity and (2) studies linking gut‐brain interactions with microglia function.

In our studies, MAL‐BG mice persistently honed to the prior MWM platform, displaying impaired memory extinction and poor learning plasticity (Figure 1d–f), accompanied by microglial alterations. Transcriptional profiling revealed increased complement expression and phagocytic transcriptional markers within MAL‐BG microglia (Figure 3b–d), supporting recent findings linking microglial phagocytic processes and brain plasticity. Wang et al. demonstrated that microglia‐dependent phagocytosis informs memory forgetting via synaptic pruning. Impairing microglial phagocytosis via inhibition of complement pathways reduced forgetting and memory extinction 35 days following a contextual fear conditioning test (Wang et al., 2020). Independent malnutrition models also linked poor spatial memory to aberrant synaptic pruning. Maternal ω3 PUFA deficiency increased complement‐dependent synaptic pruning within offspring. Inhibition of the hippocampal complement cascade via XVA‐143 (CR3 antagonist) mitigated poor memory responses in the Y‐maze task (Madore et al., 2020).

While we observed increased phagocytic cups on hippocampal MAL‐BG microglia, the precise role of MAL‐BG phagocytosis remains undetermined. Microglia also utilize phagocytic processes when responding to parenchymal insult (Wu et al., 2015). As MAL‐BG brains lacked significant BBB deficits or proinflammatory cytokines (TNF‐α, IL‐6), phagocytic features may reflect an intrinsic function (e.g., phagocytic synaptic pruning), rather than environmental response (e.g., phagocytosis of bacterial debris, systemic inflammation).

How does fecal‐oral contamination promote aberrant microglial features in the context of malnutrition? In 2015, Erny et al. first reported that commensal gut microbes influence microglia maturation and function utilizing germ‐free models. Germ‐free mice exhibited an immature microglial phenotype characterized by increased volume, process length, and process complexity (branching). Researchers identified bacterial‐derived short‐chain fatty acids (SCFAs) as a critical microbial regulator of microglial maturation and immune function (Erny et al., 2015). Diet‐induced neuroinflammation has also been linked to microglial modulation. Valdearcos et al. demonstrated that overnutrition triggers neuroinflammation promoting microglial activation. Mice fed a high‐fat diet displayed decreased microglial volume and gliosis (microglial proliferation) within the mediobasal hypothalamus, impairing systemic metabolic function (Valdearcos et al., 2017).

Like Erny et al., we report that specific shifts in gut microbiome composition impact microglia (Figures 3b–e and 4g). While GF mice lack SCFAs, we previously reported comparable SCFA levels within the gastrointestinal tract of our healthy and malnourished models (Brown et al., 2015). As diet‐dependent metabolic shifts also shape microglial function (Madore et al., 2020; Valdearcos et al., 2017), we profiled the hippocampal metabolome. Previous metabolomic profiling of the malnourished small intestine revealed marked alteration of amino acid and lipid metabolism (Brown et al., 2015), broadly matching reported shifts in the serum metabolome of children treated for malnutrition (Di Giovanni et al., 2016). Similar to the intestinal metabolomic profile (Brown et al., 2015), the MAL and MAL‐BG hippocampal metabolome exhibits perturbed lipid metabolism, particularly PUFA pathways.

As noted, PUFAs serve as essential phospholipid components, participating in lipid signaling, inflammatory regulation, neurodevelopment, and microglial‐dependent synaptic plasticity within the brain (Bazinet & Layé, 2014; Madore et al., 2020). While malnutrition shaped ω6 and ω3 PUFA metabolism, overall PUFA levels were comparable across CON, MAL, and MAL‐BG mice. These findings highlight persistent PUFA maintenance within the brain, a metabolic resilience not observed in the liver, which displayed consistent, dietary‐induced reduction of PUFA members (Bauer et al., 2020). Both MAL‐BG mice and MAL mice displayed similar PUFA profiles, suggesting that PUFA metabolism was largely driven by diet rather than microbial exposure. The MAL‐BG brain, however, exhibited significant elevation of oxidative stress markers, including MDAL (PUFA‐specific lipoxidation biomarker), highlighting a putative role for aberrant PUFA metabolism and lipoxidation in malnutrition neuropathology.

To assess reversibility of MAL‐BG features, we developed PAO (ω3‐PUFA + antioxidant vitamins) dietary intervention, see Supplemental File 1. In contrast to the ω6‐based standard and malnourished diets, CON‐PAO and MBG‐PAO intervention utilized ω3‐EPA as the primary PUFA source. ω3‐PUFAs are required for appropriate cognitive function via microglial phagocytic processes (Madore et al., 2020), while reduction of dietary ω6/ω3 intake has been linked to anti‐inflammatory/oxidative outcomes (Augusto et al., 2017; McNamara & Almeida, 2019; Tapiero et al., 2002), To further promote antioxidation, PAO diets were modestly fortified with antioxidant vitamin C/E.

PAO supplementation largely reversed MAL‐BG cognitive deficits and altered PUFA profiles. Microglial phagocytic features and oxidative stress markers, however, were not influenced by PAO fortification. Surprisingly, oxidative stress markers were frequently higher in healthy controls during the PAO study (Figure S8j) compared to earlier analyses (Figure 4e and Figure S6d), with CON/CON‐PAO oxidative levels resembling previously reported malnourished phenotypes. Unlike initial oxidative profiling, mice in the PAO trial underwent MWM testing. The MWMT is a multi‐day study involving repeated stress exposures (e.g., water avoidance, body temperature fluctuations). Behavioral test stressors have a profound impact on microglial morphology and activation (Hinwood et al., 2012; Walkera et al., 2013), as well as systemic oxidative responses (Seo et al., 2012). PAO microglial analyses were conducted on C57BL/6 mice, 4 days following MWMT. Whether MWMT‐induced stress influenced baseline controls remains unknown. Comparable oxidative profiling may also reflect modest PAO antioxidant capacity. Importantly, the PAO diets replaced ω6‐rich oils, but were not enriched with ω3 PUFAs, in order to maintain caloric equivalence across experimental diets. Further ω3 PUFAs‐enrichment and/or increasing antioxidant content and/or chemically blocking lipoxidative pathways (e.g., ω6 oxidative blockade with non‐steroidal anti‐inflammatory drugs) may reveal altered oxidative patterns and/or microglial phagocytic features not present with PAO fortification (Tapiero et al., 2002). Finally, mice fed PAO diets generally ate less and grew smaller than respective dietary counterparts (Figure S8a–c). While PAO diets were not designed to target growth deficits, reduced dietary intake and subsequent anthropometric shortcomings likely contributed to modest intervention outcomes.

Malnutrition and fecal‐oral contamination frequently coexist (Black et al., 2013; Guerrant et al., 2013), and research assessing gut‐brain interactions within this framework are critical to improving understanding of cognitive consequences of early‐life malnutrition. This work supports research linking enteropathogenic burden and microbial dysbiosis with impaired cognitive development (Black et al., 2013; Investigators, 2018). While neurocognitive consequences of childhood malnutrition are unquestionably shaped by societal, economical, and political factors (Smith & Haddad, 2015; Webb et al., 2018), our findings demonstrate that poor diet and specific gut microbes may trigger neuropathologies independent of these “external” influences.

The altered gut‐brain axis has emerged as a critical regulator of CNS function and, by extension, a potent therapeutic target (Bauer et al., 2019). In our malnourished model, dietary PAO intervention was sufficient to reverse impaired learning plasticity in malnourished mice exposed to repeated fecal‐oral contamination. These findings may support recent efforts to enrich ω6‐based therapeutic foods with ω3 PUFAs in the context of severe acute malnutrition (Jones et al., 2015). Largescale WASH (water, sanitation, and hygiene) and SHINE (Sanitation, Hygiene, Infant Nutrition Efficacy) interventions have yielded modest reductions of fecal‐oral contaminants in malnourished communities without linear growth (stunting) benefits (Pickering et al., 2019). Combining diet‐ and microbial‐targeted interventions, however, modestly improved early cognitive measures in a Bangladeshi WASH trial (Tofail et al., 2018), while diets designed to benefit microbiome development increased plasma biomarkers of neurodevelopment in children with persistent moderate acute malnutrition (Gehrig et al., 2019). Whether interventions targeting fecal‐oral microbial exposures will robustly improve learning deficits associated with childhood malnutrition remains to be determined (Aboud & Yousafzai, 2018).

Despite concerted intervention efforts, global malnutrition persists and remains a silent specter during these unprecedented times (Osendarp et al., 2021). The MAL‐BG model reveals that specific gut bacteria and malnutrition contribute to cognitive deficits, likely involving microglial and PUFA metabolic shifts. We anticipate that these findings will provide valued insight into dynamic gut microbiota‐brain interactions and inform intervention strategies to mitigate lasting consequences of childhood malnutrition.

## CONFLICT OF INTEREST

The authors declare no competing financial interests.

## AUTHOR CONTRIBUTIONS

Kylynda C. Bauer wrote the manuscript and planned, performed, or analyzed experiments. Elisa M. York developed the project and manuscript, conducting microglia imaging and staining. Louis‐Philippe Bernier conducted microglial analyses for PAO study. Eric M. Brown developed the MAL‐BG model and along with Kelsey E. Huus, Mihai S. Cirstea, Nina Radisavljevic, Sarah Woodward, Tahereh Bozorgmehr, and Zakhar Krekhno conducted and analyzed MAL‐BG experiments. Charisse N. Petersen conducted flow cytometry experiments. Amy H. Y. Lee supported RNA‐Seq analyses and Jun Han performed metabolomics. Victoria Ayala and Rebeca Berdún developed and analyzed fatty acid profiles and oxidative stress markers. Robert E. Hancock, Brian A. MacVicar, and Barton Brett Finlay supervised UBC trainees, project, and manuscript development.

## Supporting information


**Appendix** S1: Supplementary Information.

**Supplemental File 1** Dietary breakdown for CON, MAL, CON‐PAO, and MAL‐PAO diets, developed and prepared by Research Diets, Inc.

**Supplemental File 2** Lesion videos of CON, MAL, and MAL‐BG microglia, see also Figure S3c,d.

**Supplemental File 3** PICRUSt output from MAL and MAL‐BG fecal microbiota, see also Figure S7c

**Figure S1** Fecal‐oral contamination impacts growth during malnutrition (a) Microbial exposure promoted weight faltering in malnourished mice (*n* = 19). Tail length, a proxy for stunting, is reduced in malnourished mice. Panels from same experiment. (b) Microbial *E. coli*/bacteroidales exposures failed to trigger weight and stunting features in healthy mice (*n* = 9 CON, 5 CON‐BG, 9 MAL‐BG). Panels from same experiment. (c) The relative abundance of Enterobacteriaceae from fecal samples measured by qPCR, similar findings reported in (Brown et al., 2015). Bar graphs indicate mean and SEM with statistical significance determined by one‐way ANOVA with post hoc Dunnett's test

**Figure S2** Malnutrition and microbes influence behavior and cognition in MAL‐BG model (a) Total immobility “resting” (left) and OFZ immobility (right) during the OFT, *n* = 21 CON, 22 MAL, 17 MAL‐BG. (b) Total distance traveled, representative OFT experiment. (c) Set‐up (left) and results (right) from the light–dark test. A measure of anxiety‐like behavior, the light–dark box is comprised of an open light region and enclosed dark region. CON, MAL, and MAL‐BG exhibit comparable behavior within the light–dark box. (d) NORT schematic showing the familiarization and recall set‐up. During familiarization, mice exhibit impartial object exploration (interaction ratio ~ 1). All groups distinguished the novel object during recall (novel: old interaction ratio > 1). NORT interactions were scored by a blinded observer. (e) Total mouse‐object interaction time (novel and old object interaction) for the NORT as recorded by a blinded observer. (f) MWMT set‐up: during habituation, mice were released from the same position and learned to locate a visible platform, platform location moved after each trial. During learning phases (acquisition, reversal) individual mice attempted to locate a hidden platform based on spatial memory and external cues. Mice entered the pool at variable locations (north, south, east, and west quadrants); trial order and location entries were randomized prior to testing. Habituation and learning trials lasted 60 s each with a rest period. Individual mice were placed in an empty MWM (30 s swim) 24 h following the final acquisition and reversal trial. (g) Average swim speed for the initial (top) and final (bottom) free swims. (h) The escape latency for the initial reversal learning trial. Mice that failed to locate the new platform location within 60 s were gently guided to the platform, following trial (failed trials represented at the dotted line). (i) Total entries to the prior platform location of each mouse across four trials, for the 1st (top) and 2nd (bottom) day of reversal learning. Data from (c–i) are subsets of mice from two independent mouse experiments, *n* = 13–16/group. Graphs indicate mean and SEM with statistical significance determined by one‐way ANOVA with post hoc Tukey's test (OFT) or post hoc Dunnett's test (NORT, MWMT); MWM/T, Morris water maze/test; NORT, novel object recognition test, n.s., non‐significant; OFT, open field test; OFZ, open field zone

**Figure S3** Microglial morphology and motility characterization (a) MAL and MAL‐BG hippocampal microglial volume alterations were independent of process branching. 3DMorph analyses with data normalized to the controls from four independent experiments, *n* = 13 CON, 13 MAL, and 12 MAL‐BG. (b) Averaged microglial process additions and retractions across 10 min in ex vivo CX3CR1^+/EGFP^ CON, MAL, and MAL‐BG hippocampal tissues, *n* = 9/group. Motility indices determined by a custom MATLAB program that identified pixel additions/removal in eGFP+ cells. (c) Representative images from two‐photon microscopy of the hippocampal CA1 region prior, at, and following lesion induction via intensive two‐photon laser scanning, *n* = 6 CON, 8 MAL, and 8 MAL‐BG. Videos in Supplemental File 2 (CON image from same animal in video file). (d) Microglial process response to lesion region: mean fluorescent intensity/microscopy frames (left) and % microglial processes entering lesion region at experimental endpoint (right). Lesion experiments conducted on a subset of mice utilized for microglial morphology analyses. Mice in (b–d) a subset from experiments presented in a. Graphs indicate mean and SEM with statistical significance determined by one‐way ANOVA with post hoc Tukey's test; n.s., non‐significant

**Figure S4** Altered functional profile in MAL‐BG microglia (a) RNA‐Seq was conducted on CD11b+ population from whole brain tissue, *n* = 4 CON, 5 MAL, and 7 MAL‐BG. Representative flow cytometry gating verifying microglial enrichment (CD11b^high^/CD45^low^ population) following Miltenyi Biotec Adult Brain Dissociation kit and CD11b separation. (b) Representative flow cytometry gating (left) of CX3CR1 gMFI and frequency (% microglia) from an independent cohort (right), supporting RNA‐Seq findings, *n* = 5 CON, 6 MAL, 12 MAL‐BG. (c) Whole brain qPCR results from an independent mouse cohort: while assessed DEGs did not reach statistical significance by RT‐qPCR, overall patterns support microglial *Sirpa* (phagocytic marker) and *Ctsd* (lysosomal marker) RNA‐Seq results. Fold change and ddct values plotted, *Hprt* provided the endogenous control. (d) ReactomePA network enrichment visualizations for MAL‐BG versus CON (*left*) and CON versus MAL‐BG (*right*): *Padj* < 0.05, FC >1.5, data from RNA‐Seq experiment in a. Bar graphs indicate mean and SEM with statistical significance determined by Kruskal‐Wallis with post hoc Dunn's test

**Figure S5** The MAL‐BG brain lacks significant neuroinflammation and BBB disruption (a) Proinflammatory cytokines (TNF‐α, IL‐6) from cortical brain tissues, cytokine levels normalized to tissue weight (*n* = 19 CON, 21 MAL, 16–17 MAL‐BG), data from three independent experiments. (b) TNF‐α and IL‐6 levels in CON, MAL, and MAL‐BG sera, data from two independent experiments, *n* = 17 CON, 18 MAL, 14 MAL‐BG. (c) Representative flow cytometry gating for inflammatory microglia panel. Microglia reported as CD11b^high^/F480^high^ within a CD45^low^ cell population. (d) Percent microglia of CON, MAL, and MAL‐BG cells following microglia isolation. Microglia identified as CD11b^high^/F480^high^ within a CD45^low^ cell population. TLR4, CD86, and MHC Class II gMFI and frequency (% microglia) presented. (e) MAL‐BG mice exhibit a decrease in Tjp1 (tight junction protein 1, zonula occludens 1) expression; qPCR from murine small intestine (ileal tissue), *n* = 10. Detailed GI permeability measures presented in Brown et al., 2015. (f) Quantification of IgG immunostaining revealed low levels of IgG antibodies within the brain parenchyma, *n* = 4/experimental group. High‐IgG presence in control photothrombotic brain tissue (ischemic stroke model), data not reported. (g) Averaged biocytin fluorescent intensity following biocytin‐TMR tail‐vein injection. (h) Biocytin‐TMR intensity across murine CNS slices, each line represents a mouse, symbols denote slice, *n* = 4/group. (i) Representative CON, MAL, and MAL‐BG cortical slices (bottom) with matched rostral ➔ caudal CNS images, biocytin‐TMR appears white. IgG and biocytin data from the same experimental dataset. Bar graphs indicate mean and SEM with statistical significance determined by Mann–Whitney two‐tailed test (qPCR), Kruskal‐Wallis with post hoc Dunn's test (flow cytometry), or one‐way ANOVA with post hoc Dunnett's test (cytokines, IgG, biocytin); CBLM, cerebellum; gMFI, geometric mean fluorescence intensity; n.s., not significant; PFC, prefrontal cortex; SSC, side scatter

**Figure S6** Altered hippocampal metabolomics and PUFA metabolism in malnourished mice (a) PCA (+/− ion channels) and PLSDA (+ ion channel) of untargeted metabolomics from murine hippocampi, *n* = 5/group. (b) Putative *m/z* identification for differentially abundant metabolites determined by Metaboanalyst v. 3.0/4.0 (one‐way ANOVA, *Padj* < 0.05, post‐hoc Fischer's LSD), features annotated against KEGG and METLIN databases. (c) Chow weights/day normalized to number of mice per cage. Chow consumption data from three cages (*n* = 9/group), averaged across three 24 h timepoints, each symbol represents a cage. (d) HAVA and CEL levels from murine cortical tissue. Data normalized to tissue mol lysine. (*n* = 7–10/group). Bar graphs indicate mean and SEM with statistical significance determined by Kruskal‐Wallis post hoc Dunn's test (chow consumption) or one‐way ANOVA with post hoc Tukey's test (oxidative stress)

**Figure S7** Diet and fecal‐oral contamination alter gut microbiota and promote systemic oxidative stress (a) β (Unweighted UniFrac) and α (Shannon) diversity analyses from 16S rRNA fecal microbiota data, microbiome analyses conducted using QIIME2 (v. 2018.2), *n* = 4/group. (b) Relative abundance of MAL‐BG gavage components, including *Parabacteroides distasonis* and *Bacteroides ovatus*, as well as the Bacteroides genus and Enterobacteriaceae family (*E. coli* family). (c) PICRUSt analyses to explore functionality shifts between MAL and MAL‐BG fecal microbiome, pathways annotated with MetaCyc (lower left table), only *Padj* < 0.01 presented, full PICRUSt output in Supplemental File 3b. Microbiome analyses from the same experiment. Graphs presented in b indicate mean and SEM with statistical significance determined by Kruskal‐Wallis with post hoc Dunn's test; CI, confidence intervals; n.s., not significant

**Figure S8** PAO diet shapes cognition and systemic PUFA profiling (a) Model of dietary PAO (ω3 PUFA/antioxidant‐associated supplement) intervention. A subset of the CON and MAL‐BG models (MBG) were placed on isocaloric CON‐PAO and MBG‐PAO diets. The PAO diet; however, contained an ω3 PUFA fat source and was enriched with elevated antioxidant‐associated vitamins, see Supplemental File 1 for full dietary breakdown. Data was assessed 54 days following start of the diet. (b) PAO mice displayed increased weight faltering (left) and stunting (tail length: right), compared to respective controls. (c) Chow consumption data from two cages (*n* = 8/group) across a 24 h timepoint approximately halfway through study. (d) Swim speed averaged across four trials from Day 1_A_ (initial acquisition learning: left) and final experimental day (Day 2_R_: right). (e) Total entries within the prior platform area for each mouse/day of reversal learning (left = Day 1_R_ left, right = Day 2_R_). (f) Microglial morphology (cell volume, territory volume, and endpoints) quantified with 3DMorph software and normalized to the CON group. (g) Percent of mice that exhibited microglial phagocytic feature/s within hippocampal regions (h) Relative PUFA abundance from cortical tissue normalized to CON mice. (i) Ratio of mol% ω3/ω6 PUFA levels from liver tissue. (j) Oxidative profiling from cortical tissue: HAVA (protein oxidation), CEL (glycoxidation), CML (glycoxidation/lipoxidation), and MDAL (PUFA lipoxidation). Data normalized to mol lysine. Panels from same experimental round (*n* = 5–8/group). Bar graphs present mean and SEM with statistical significance determined by one‐way ANOVA with post hoc Tukey's test (MWMT, microglia, fatty acid analyses) or Kruskal‐Wallis post hoc Dunn's test (chow consumption): MWMT, Morris water maze test; n.s., not significant

## Data Availability

The data sets generated during and/or analysed during the current study are available from the corresponding author on reasonable request. Hippocampal metabolomic dataset presented in Supplemental File 3, raw data stored at metabolomicsworkbench.org: ST001366. Raw 16S and RNA‐Seq data accessed at the following links. Raw 16S Data: (https://www.ncbi.nlm.nih.gov/bioproject/PRJNA574479) Raw Fastq RNA‐Seq Data: (https://www.ncbi.nlm.nih.gov/geo/query/acc.cgi?acc=GSE138182) R code for DESeq2/ReactomePA and Microbiome analyses: Glia_RMarkdown
