# Supplementary material for: Gut microbes shape microglia and cognitive function during malnutrition
Source: Glia. 2022 Jan 12;70(5):820–41. doi: 10.1002/glia.24139 (PMC9305450; doi:10.1002/glia.24139)
Supplement: Supplementary file 1 — Appendix S1: Supplementary Information. [file GLIA-70-820-s006.docx]

RNA-Seq and Microbiome Analyses

## Kylynda C. Bauer 2020-04-16

**Contents**

[Load library packages and set working directory](#_bookmark0) 1

[DESeq2 Pipeline](#_bookmark1) 2

[Visualize DESeq2](#_bookmark2) 3

[ReactomePA Pipeline](#_bookmark3) 7

[Visualize ReactomePA](#_bookmark4) 7

[Microbiome Analyses](#_bookmark5) 13

[Microbiome Visualization](#_bookmark6) 13

[Pipelines and helpful URLs visited during analyses](#_bookmark7) 15

# Load library packages and set working directory

*# RNA-Seq_DESeqAnalyses* **library**(DESeq2) **library**(ggplot2) **library**(pheatmap) **library**(RColorBrewer)

*# + RNA-Seq_ReactomePAAnalyses* **library**(clusterProfiler) **library**(dplyr) **library**(org.Mm.eg.db) **library**(ReactomePA) **library**(readr) **library**(remotes) **library**(tidyr) **library**(tidyverse)

*# + Microbiome_Analyses* **library**(phyloseq) **library**(psych) **library**(qiime2R) **library**(tibble)

# DESeq2 Pipeline

**setwd**("~/Desktop/RNASEQ_Plots/forRMarkdown_14")

*#Read qualitative and quantitative txt files into R_follow DESeq format*

coldataf <- **read.delim**("coldataf.txt") countdataf <- **read.delim**("countdataf.txt")

*#Note: Gene IDs may be changed to dates or misformatted #if opened in Excel/non-text file applications*

*#I've changed the row names to match my counts.genes files*

*#Names should be identical and in the identical order as countdataf/coldataf*

countdR <-**read.delim**("CON_A3.counts.genes", header=FALSE)

**names**(countdR) **rownames**(countdataf) <- countdR**$**V1 countdataf**$**X <- NULL **head**(countdataf)

**head**(coldataf)

*#Run Deseq2*

dds <- **DESeqDataSetFromMatrix**(countData = countdataf,

colData = coldataf,

design = **~** batch **+** condition)

dds

*#Note: Re-level data based on control group, if needed, sample code below#*

dds**$**condition <- **relevel**( dds**$**condition, "CON" )

*#Prefilter remove low counts (sum less than 10 in samples in the smallest group, CON n=4)*

keep <- **rowSums**(**counts**(dds) **>** 10) **>=** 4 dds <- dds[keep,]

**summary**(dds)

*#Get Differentially Expressed Genes List*

dds <- **DESeq**(dds)

res1f <- **results**(dds, contrast=**c**("condition","MAL","CON")) res2f <- **results**(dds, contrast=**c**("condition","MAL_BG","CON")) res3f <- **results**(dds, contrast=**c**("condition","MAL_BG","MAL"))

*#Reorder results based on the padj value* resOrdered1 <- res1f[**order**(res1f**$**pvalue),] resOrdered2 <- res2f[**order**(res2f**$**pvalue),] resOrdered3 <- res3f[**order**(res3f**$**pvalue),]

*#Check how many genes pass a padj value of 0.05*

**sum**(res1f**$**padj **<** 0.05 **&** res1f**$**log2FoldChange **>** 0.58, na.rm=TRUE)

## [1] 0

**sum**(res1f**$**padj **<** 0.05 **&** res1f**$**log2FoldChange **< -**0.58, na.rm=TRUE)

## [1] 0

**sum**(res2f**$**padj **<** 0.05 **&** res2f**$**log2FoldChange **>** 0.58, na.rm=TRUE)

## [1] 1854

**sum**(res2f**$**padj **<** 0.05 **&** res2f**$**log2FoldChange **< -**0.58, na.rm=TRUE)

## [1] 2831

**sum**(res3f**$**padj **<** 0.05 **&** res3f**$**log2FoldChange **>** 0.58, na.rm=TRUE)

## [1] 1871

**sum**(res3f**$**padj **<** 0.05 **&** res3f**$**log2FoldChange **< -**0.58, na.rm=TRUE)

## [1] 2583

*#Export the results files for use*

**write.csv**(**as.data.frame**(resOrdered1), file="condition_MAL_v_CON_results.csv")

**write.csv**(**as.data.frame**(resOrdered2), file="condition_MALBG_v_CON_results.csv")

**write.csv**(**as.data.frame**(resOrdered3), file="condition_MALBG_v_MAL_results.csv")

# Visualize DESeq2

*#PCA with ggplot*

*#Transform raw counts data_log2 scale / ntdf = log2(n+1)*

vsdf <- **vst**(dds, blind=FALSE) ntdf <- **normTransform**(dds)

*#Making a PCA plot*

**plotPCA**(vsdf, intgroup=**c**("condition", "batch"))

**plotPCA**(vsdf, intgroup=**c**("condition"))

*#PCA aesthetics1*

pcaDataf <- **plotPCA**(vsdf, intgroup=**c**("condition"), returnData=TRUE) percentVar <- **round**(100 *** attr**(pcaDataf, "percentVar")) **ggplot**(pcaDataf, **aes**(PC1, PC2, color=condition)) **+**

**geom_point**(size=4) **+**

**scale_color_manual**(values = **c**("black","blue3","deeppink3")) **+ xlab**(**paste0**("PC1: ",percentVar[1],"% variance")) **+ ylab**(**paste0**("PC2: ",percentVar[2],"% variance")) **+ coord_fixed**() **+**

**stat_ellipse**(linetype = 1)

*#PCA aesthetics2*

pcaplayf <- **ggplot**(pcaDataf, **aes**(PC1, PC2, color=condition)) **+ geom_point**(size=4) **+**

**scale_color_manual**(values = **c**("black","blue3","deeppink3")) **+ xlab**(**paste0**("PC1: ",percentVar[1],"% variance")) **+ ylab**(**paste0**("PC2: ",percentVar[2],"% variance")) **+ coord_fixed**() **+**

**stat_ellipse**(linetype = 1)

*#Finalized PCA version*

pcaplayf **+ theme_bw**() **+ theme**(legend.title = **element_blank**()) **+ theme**(legend.position = **c**(0.90, 0.09)) **+**

**theme**(legend.text = **element_text**(size=10))

20


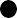

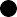

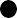

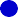

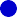

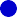

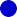

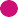

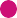

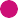

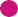

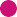

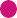

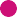

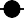

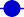

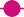


CON MAL

MAL_BG

10

0

PC2: 8% variance

−10

−20

−50 −25 0 25

PC1: 75% variance

*#Heatmap with DESeq and pheatmap #Heatmap_aesthetics1*

*#Top 50 genes selected*

select <- **order**(**rowMeans**(**counts**(dds,normalized=TRUE)), decreasing=TRUE)[1**:**50]

df <- **as.data.frame**(**colData**(dds)[,**c**("condition")], row.names = **colnames**(dds)) **colnames**(df) <- "Group"

ann_colors = **list**(Group = **c**(CON = "black", MAL = "blue3", MAL_BG = "deeppink2"), DEG = **c**(t = "navy", t_in_CON = "royalblue4",

t_in_MAL = "royalblue1", f= "yellow"))

**head**(df)

*#Heatmap_aesthetics2*

**pheatmap**(**assay**(ntdf)[select,], annotation_legend = TRUE,

color = **colorRampPalette**(**rev**(**brewer.pal**(n=10, name = "RdGy")))(10), cluster_rows=FALSE, show_rownames=TRUE,

cluster_cols=TRUE, annotation_col=df, annotation_colors=ann_colors)

*#Heatmap_row_annotations*

*#Function annotations determined by GeneCards and NIH #Created a txt file to add DEG and functional data* annorow1b <-**read.delim**("top50useuse.txt")

annorow1b <-**as.data.frame**(annorow1b, header = TRUE, row.names = **rownames**((ntdf)[select,])) annorow1b**$**Gene = NULL

**head**(annorow1b)

select <- **order**(**rowMeans**(**counts**(dds,normalized=TRUE)), decreasing=TRUE)[1**:**50]

df <- **as.data.frame**(**colData**(dds)[,**c**("condition")], row.names = **colnames**(dds)) **colnames**(df) <- "Group"

**head**(df)

ann_colors = **list**(Group = **c**(CON = "black", MAL = "blue3", MAL_BG = "deeppink2"),

DEG = **c**(t = "slateblue4", t_in_CON = "slateblue", t_in_MAL = "slateblue1", f = "yellow"), Function = **c**(Immune = "peru", Lipid_Processing= "magenta", Lysosome = "darkmagenta",

Phagocytosis_SynapticPruning = "blueviolet", Other = "wheat"))

**pheatmap**(**assay**(ntdf)[select,], annotation_legend = TRUE,

color = **colorRampPalette**(**rev**(**brewer.pal**(n=10, name = "RdGy")))(10), cluster_rows=FALSE, show_rownames=TRUE,

cluster_cols=TRUE, annotation_col=df, annotation_row = annorow1b, annotation_colors=ann_colors)


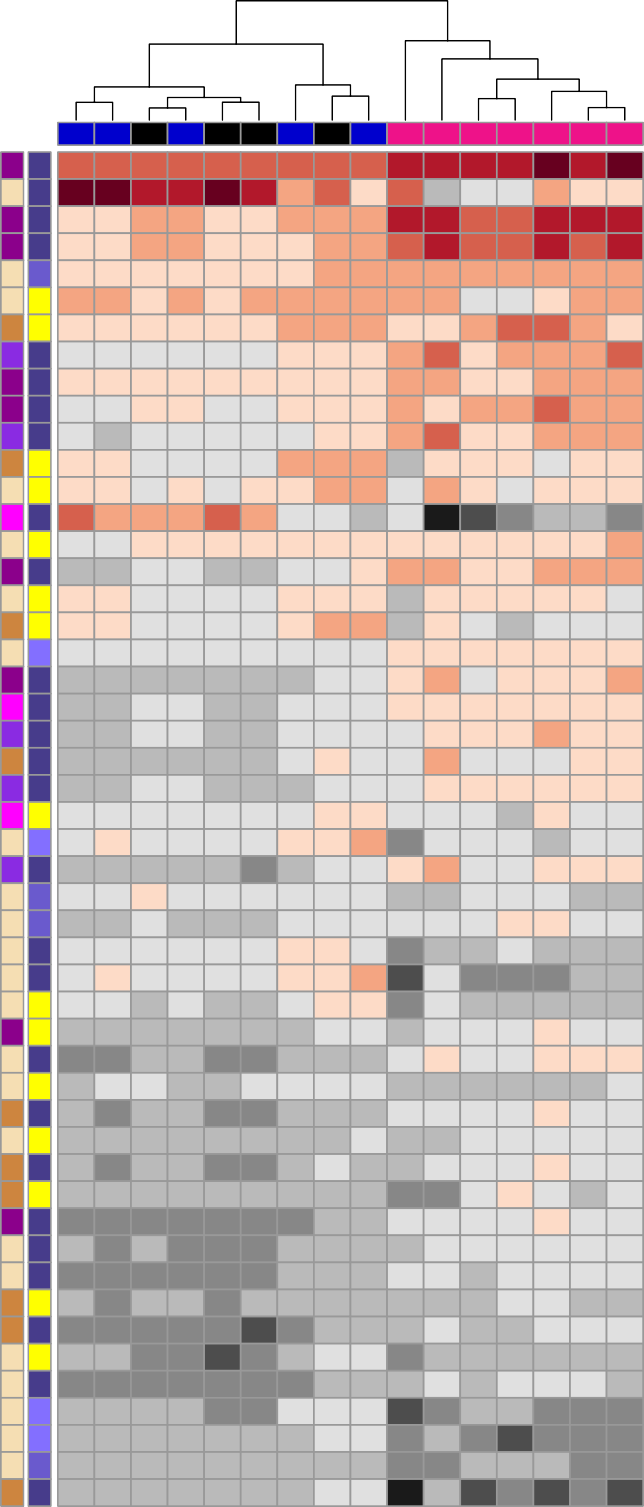
**Group** Cst3 Ttr

Hexb

Ctsd

**Group**


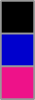

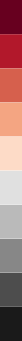
18 CON MAL

17 MAL_BG

Actb Malat1 Csf1r Cx3cr1 Psap Ctss C1qa Jun Zfp36 Enpp2 Tmsb4x Lgmn Rhob Junb Sparc Laptm5 Serinc3 C1qc Jund C1qb Apoe Fos Sirpa Eef1a1 Itm2b Kctd12 Egr1 Btg2 Ctsb

4632428N05Rik

Glul Selplg Sepp1 Fcrls P2ry12 Grn Tmem119 Marcks Mpeg1 Unc93b1 Txnip Itgb5 Ivns1abp H3f3b Fth1

Atf3

MALBG_7 MALBG_4 MALBG_1 MALBG_6 MALBG_5 MALBG_2 MALBG_3 MAL_4 CON_1 MAL_1 CON_4 CON_2 MAL_5 CON_3 MAL_3 MAL_2

**DEG**

**Function**

16 **Function**

15 Immune Lipid_Processing

|  |
| --- |
|  |
|  |
|  |
|  |

14 Lysosome

13 Phagocytosis_SynapticPruning Other

**DEG**

|  |
| --- |
|  |
|  |
|  |

t t_in_CON t_in_MAL f

# ReactomePA Pipeline

*#Create a .csv file containing DEGs*

*#(Fold Change > 1.5 and padj <0.05_format for ReactomePA) #Change gene symbols to the entrezID and run ReactomePA pipeline #CON file = CONvMALBG*

IDs1 <- **read_csv**("FC15padj05_MALBGvCON.csv")

ENTREZ1 <- **bitr**(geneID = IDs1**$**Gene, fromType = "SYMBOL", toType = "ENTREZID", OrgDb = "org.Mm.eg.db")

**write.csv**(ENTREZ1, file = "ENTREZ1.csv")

Pathway1 <- **enrichPathway**(gene = ENTREZ1**$**ENTREZID, pvalueCutoff = 0.05, readable = T, organism = "mouse")

IDs2 <- **read_csv**("FC15padj05_MALBGvMAL.csv")

ENTREZ2 <- **bitr**(geneID = IDs2**$**Gene, fromType = "SYMBOL", toType = "ENTREZID", OrgDb = "org.Mm.eg.db")

**write.csv**(ENTREZ2, file = "ENTREZ2.csv")

Pathway2 <- **enrichPathway**(gene = ENTREZ2**$**ENTREZID, pvalueCutoff = 0.05, readable = T, organism = "mouse")

IDs3 <- **read_csv**("FC15padj05_CON.csv")

ENTREZ3 <- **bitr**(geneID = IDs3**$**Gene, fromType = "SYMBOL", toType = "ENTREZID", OrgDb = "org.Mm.eg.db")

**write.csv**(ENTREZ3, file = "ENTREZ3.csv")

Pathway3 <- **enrichPathway**(gene = ENTREZ3**$**ENTREZID, pvalueCutoff = 0.05, readable = T, organism = "mouse")

# Visualize ReactomePA

*#Dotplot*

**dotplot**(Pathway1, showCategory=20)

**dotplot**(Pathway2, showCategory=8)

**dotplot**(Pathway3, showCategory=30)

*#Dotplot_aesthetics1 (combine MAL and MALBG)* MALBGvCON <-**as.data.frame**(Pathway1, header = TRUE) MALBGvMAL <- **as.data.frame**(Pathway2, header = TRUE) MALBGvMAL <- **as.data.frame**(Pathway2, header = TRUE)

MALBGvCON**$**Dataset <- "MALBGvCON" MALBGvMAL**$**Dataset <- "MALBGvMAL"

D <- **merge**(MALBGvCON, MALBGvMAL, all=TRUE)

**head**(D)

p <- **ggplot**(D, **aes**(x = GeneRatio, y = **fct_reorder**(Description, Count))) **+ geom_point**(**aes**(size = Count, color = p.adjust)) **+**

**theme_bw**(base_size = 14) **+ scale_colour_gradient**(low="blue", high = "red") **+ ylab**(NULL)

p **+ facet_grid**(.**~**Dataset)

*#Dotplot_aesthetics2_now we need to make the gene ratios from ratios into integers #I did this manually in the .csv file*

**write.csv**(MALBGvCON, file = "MALBGvCON.csv")

**write.csv**(MALBGvMAL, file = "MALBGvMAL.csv")

*#read back in the file with integers / integer files = MALBGvCONi and MALBGvMALi*

*#Finalized Images*

a <- **read.csv**("MALBGvCONi.csv", header = TRUE) b <- **read.csv**("MALBGvMALi.csv", header = TRUE) E <- **merge**(a,b, all=TRUE)

p1 <- **ggplot**(E, **aes**(x = GeneRatio, y = **fct_reorder**(Description, Count))) **+ geom_point**(**aes**(color = p.adjust, size = Count)) **+**

**theme_bw**(base_size = 14) **+ scale_colour_gradient**(low="blue", high = "red") **+ ylab**(NULL)

p1 **+ facet_grid**(.**~**Dataset)

Metabolism of RNA

MALBGvCON

MALBGvMAL

Class I MHC mediated antigen processing & presentation

Asparagine N−linked glycosylation

Antigen processing: Ubiquitination & Proteasome degradation

Metabolism of carbohydrates

Processing of Capped Intron−Containing Pre−mRNA

Neddylation

mRNA Splicing mRNA Splicing − Major Pathway Glycosaminoglycan metabolism

Cell surface interactions at the vascular wall

p.adjust

0.04


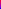


0.03

0.02

0.01

TNFR2 non−canonical NF−kB pathway Antigen processing−Cross presentation

Biosynthesis of the N−glycan precursor (dolichol lipid−linked oligosaccharide, LLO) and transfer to a nascent protein

Count

20

40

60

Sphingolipid metabolism

Synthesis of substrates in N−glycan biosythesis

SCF−beta−TrCP mediated degradation of Emi1

Keratan sulfate/keratin metabolism

N−glycan trimming in the ER and Calnexin/Calreticulin cycle

Glycosphingolipid metabolism

Basigin interactions

Calnexin/calreticulin cycle

0.025 0.050 0.075 0.025 0.050 0.075

GeneRatio

**dotplot**(Pathway3, showCategory=30)

Neuronal System Developmental Biology


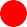

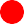

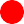

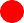

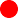

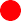

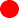

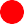

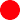

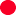

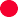

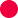

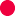

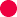

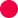

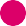

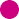

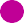

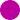

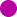

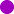

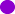

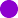

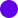

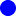

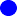

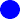


Axon guidance Transmission across Chemical Synapses Organelle biogenesis and maintenance

Cilium Assembly MAPK family signaling cascades

Neurotransmitter receptors and postsynaptic signal transmission

Cardiac conduction Rho GTPase cycle

Death Receptor Signalling Potassium Channels

Protein−protein interactions at synapses p75 NTR receptor−mediated signalling G alpha (12/13) signalling events

Intraflagellar transport Cell death signalling via NRAGE, NRIF and NADE

GABA receptor activation L1CAM interactions

NRAGE signals death through JNK

Kinesins Neurotransmitter release cycle Phase 0 − rapid depolarisation Neurexins and neuroligins

Carboxyterminal post−translational modifications of tubulin

Phase 2 − plateau phase Serotonin Neurotransmitter Release Cycle

LGI−ADAM interactions GABA A receptor activation

Presynaptic depolarization and calcium channel opening

0.02

0.04

GeneRatio

0.06

0.08

p.adjust

0.005


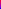


0.010

0.015

0.020

Count

25


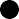

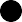

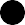


50

75

*#EnrichmentMAP #MALBGvCON = Pathway1 #CONvMALBG = Pathway3* **emapplot**(Pathway1)


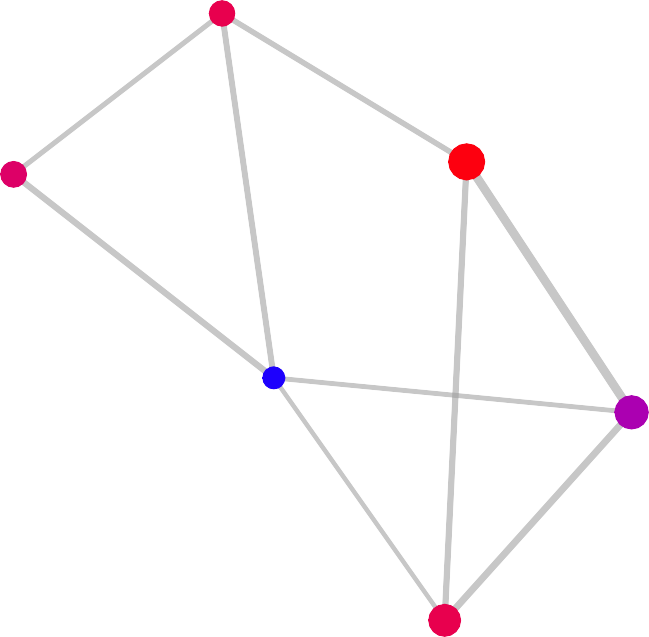
Antigen processing−Cross presentation

Class I MHC mediated antigen processing & presentation TNFR2 non−canonical NF−kB pathway

Metabolism of carbohydrates


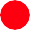

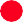

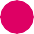


SCF−beta−TrCP mediated degradation of Emi1

Antigen processing: Ubiquitination & Proteasome degradation

Keratan sulfate/keratin metabolism Glycosaminoglycan metabolism

Neddylation

size

20


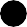

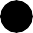

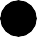


40

60


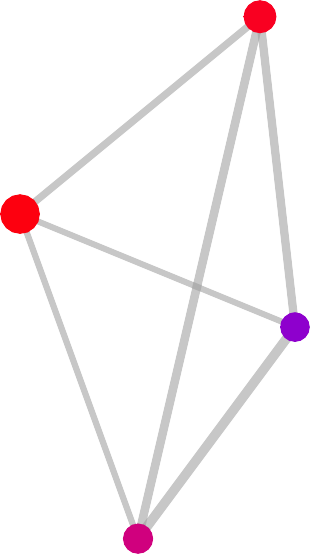
Processing of Capped Intron−Containing Pre−mRNA

p.adjust

Sphingolipid metabolism


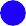

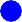


Metabolism of RNA

0.01

0.02


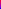


0.03

0.04

Synthesis of substrates in N−glycan biosythesis


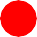

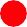

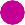


mRNA Splicing − Major Pathway

Glycosphingolipid metabolism

Asparagine N−linked glycosylation

mRNA Splicing

hesis of the N−glycan precursor (dolichol lipid−linked oligosaccharide, LLO) and transfer to a nascent protein

N−glycan trimming in the ER and Calnexin/Calreticulin cycle


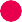

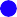


Calnexin/calreticulin cycle

**emapplot**(Pathway3)


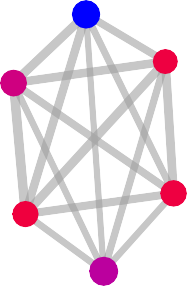
Death Receptor Signalling

NRAGE signals death through JNK

p75 NTR receptor−mediated signalling

G alpha (12/13) signalling events

Cell death signalling via NRAGE, NRIF and NADE

Rho GTPase cycle


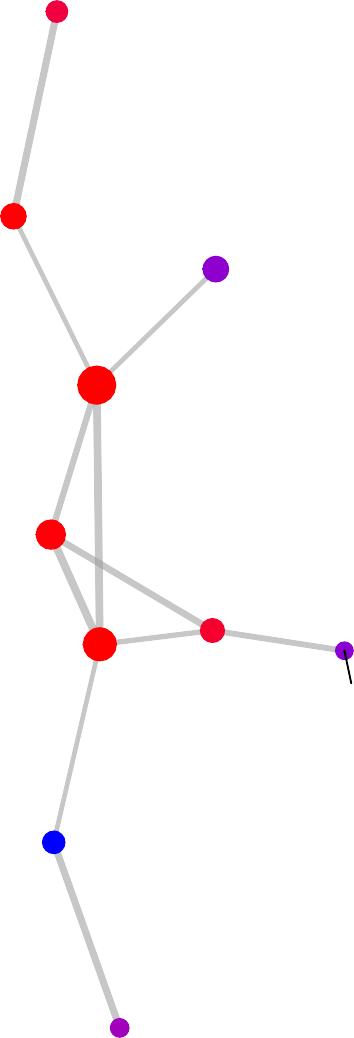
Neurexins and neuroligins

Cardiac conduction


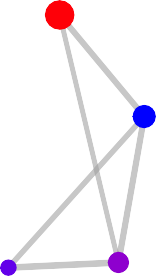
Phase 0 − rapid depolarisation

Protein−protein interactions at synapses

Phase 2 − plateau phase

Presynaptic depolarization and calcium channel opening

Potassium Channels

Neuronal System

size

25


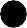

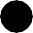

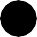


50

75


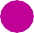
MAPK family signaling cascades

p.adjust

Neurotransmitter receptors and postsynaptic signal transmission

GABA receptor activation Transmission across Chemical Synapses

GABA A receptor activation


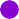
LGI−ADAM interactions

0.005

0.010


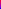


0.015

0.020


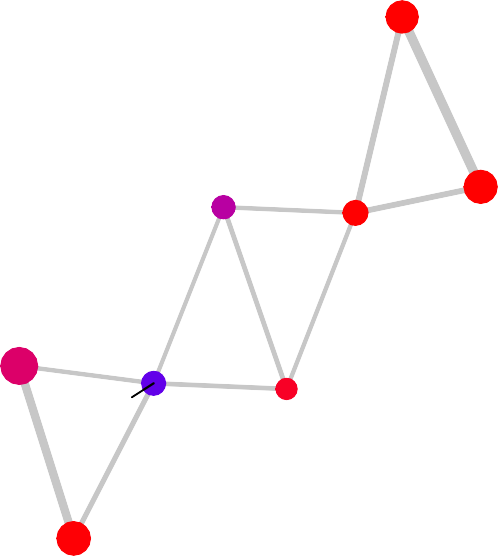
Cilium Assembly

Neurotransmitter release cycle

Kinesins

Organelle biogenesis and maintenance

Serotonin Neurotransmitter Release Cycle

Intraflagellar transport

Developmental Biology

L1CAM interactions

Carboxyterminal post−translational modifications of tubulin

Axon guidance

# Microbiome Analyses

*#Bring in Metadata*

metadata<-**read_tsv**("Mapqiime_Kcbauer_14.tsv") metadata

*#Bring in table data*

SVs<-**read_qza**("FilteredTable14.qza")

*#Bring in taxonomy*

*#convert the table into a tabular split version* taxonomy<-**read_qza**("97-taxonomy.qza") taxonomy**$**uuid

taxtable<-taxonomy**$**data **%>% as.tibble**() **%>% separate**(Taxon, sep="; ",

**c**("Kingdom","Phylum","Class","Order","Family","Genus","Species")) **head**(taxtable)

*#note: when a family or order went missing for samples it was filled with an "NA"*

*#Form a rooted tree*

tree<-**read_qza**("rooted-tree.qza") tree**$**uuid

tree**$**data

*#Alpha Diversity (additional analyses are possible e.g. Shannon)*

faithpd <-**read_qza**("faith_pd_vector.qza") faithpd**$**uuid

*#Beta Diversity (additional analyses are possible e.g. Weighted UniFrac) #determine proportion explained*

pco1<-**read_qza**("unweighted_unifrac_pcoa_results.qza") pco1**$**uuid

**head**(pco1**$**data**$**ProportionExplained) pco1**$**data**$**Vectors[1**:**5, 1**:**3]

# Microbiome Visualization

*#Make a Graph*

pco1**$**data**$**Vectors **%>%**

**rename**("#SampleID"=SampleID) **%>%** *#rename to match the metadata table*

**left_join**(metadata) **%>%**

**left_join**(faithpd**$**data **%>% rownames_to_column**("#SampleID")) **%>% ggplot**(**aes**(x=PC1, y=PC2, color=Group, size=faith_pd)) **+ geom_point**() **+**

**xlab**(**paste**("PC1: ", **round**(100*****pco1**$**data**$**ProportionExplained[1], digits = 1), "%")) **+ ylab**(**paste**("PC2: ", **round**(100*****pco1**$**data**$**ProportionExplained[2], digits = 1), "%")) **+ theme_bw**() **+**

**ggtitle**("Unweighted Unifrac")

Unweighted Unifrac

0.1

PC2: 22.1 %

0.0

−0.1

### Group

CON MAL MAL−BG


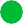

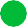

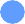

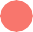

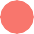

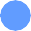

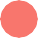

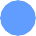

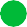

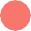

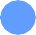


### faith_pd

5.5


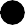
 6.0


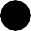
 6.5


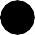
7.0

−0.1 0.0 0.1

### PC1: 32.1 %

*#aesthetics*

UU <- pco1**$**data**$**Vectors **%>%**

**rename**("#SampleID"=SampleID) **%>%** *#rename to match the metadata table*

**left_join**(metadata) **%>%**

**left_join**(faithpd**$**data **%>% rownames_to_column**("#SampleID")) **%>% ggplot**(**aes**(x=PC1, y=PC2, color=Group, size=faith_pd)) **+ geom_point**() **+**

**xlab**(**paste**("PC1: 32.1%")) **+**

**ylab**(**paste**("PC2: 22.1%")) **+ theme_bw**() **+ ggtitle**("Unweighted UniFrac")

*#Final Graph*

UU **+ scale_color_manual**(values=**c**("black","blue3","deeppink3")) **+ scale_size_continuous**(range=**c**(4,8)) **+**

**theme**(title = **element_text**(size=18, face = "bold"), axis.title.x = **element_text**(size = 16), axis.title.y = **element_text**(size = 16), legend.text = **element_text**(size = 16)) **+**

**guides**(colour = **guide_legend**(override.aes = **list**(size=5)))

**Unweighted UniFrac**

0.1 **Group**


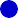

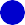

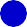

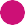

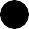

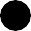

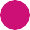

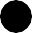


##### CON MAL MAL−BG

**PC2: 22.1%**

0.0 **faith_pd**

##### 5.5

##### 6.0

##### 6.5

##### 7.0

−0.1

−0.1 0.0 0.1

#### PC1: 32.1%

# Pipelines and helpful URLs visited during analyses

###### DESEQ Pipeline [https://bioconductor.org/packages/release/bioc/vignettes/DESeq2/inst/](https://bioconductor.org/packages/release/bioc/vignettes/DESeq2/inst/doc/DESeq2.html) [doc/DESeq2.html](https://bioconductor.org/packages/release/bioc/vignettes/DESeq2/inst/doc/DESeq2.html)

###### ReactomePA Pipeline [https://bioconductor.org/packages/release/bioc/vignettes/ReactomeP](https://bioconductor.org/packages/release/bioc/vignettes/ReactomePA/inst/doc/ReactomePA.html)A/ [inst/doc/ReactomePA.html](https://bioconductor.org/packages/release/bioc/vignettes/ReactomePA/inst/doc/ReactomePA.html)

###### Code assistance (1) <https://github.com/YuLab-SMU/DOSE/issues/20> (2) [https://www.](https://www.biostars.org/p/363491/)

###### [biostars.org/p/363491/](https://www.biostars.org/p/363491/) (3) [https://forum.qiime2.org/t/tutorial-integrating-qiime2-and-r-for-](https://forum.qiime2.org/t/tutorial-integrating-qiime2-and-r-for-data-visualization-and-analysis-using-qiime2r/4121/19) [data-visualization-and-analysis-using-qiime2r/4121/19](https://forum.qiime2.org/t/tutorial-integrating-qiime2-and-r-for-data-visualization-and-analysis-using-qiime2r/4121/19)
