# Supplementary figures and images for: Gut microbes shape microglia and cognitive function during malnutrition
Source: Glia. 2022 Jan 12;70(5):820–41. doi: 10.1002/glia.24139 (PMC9305450; doi:10.1002/glia.24139)

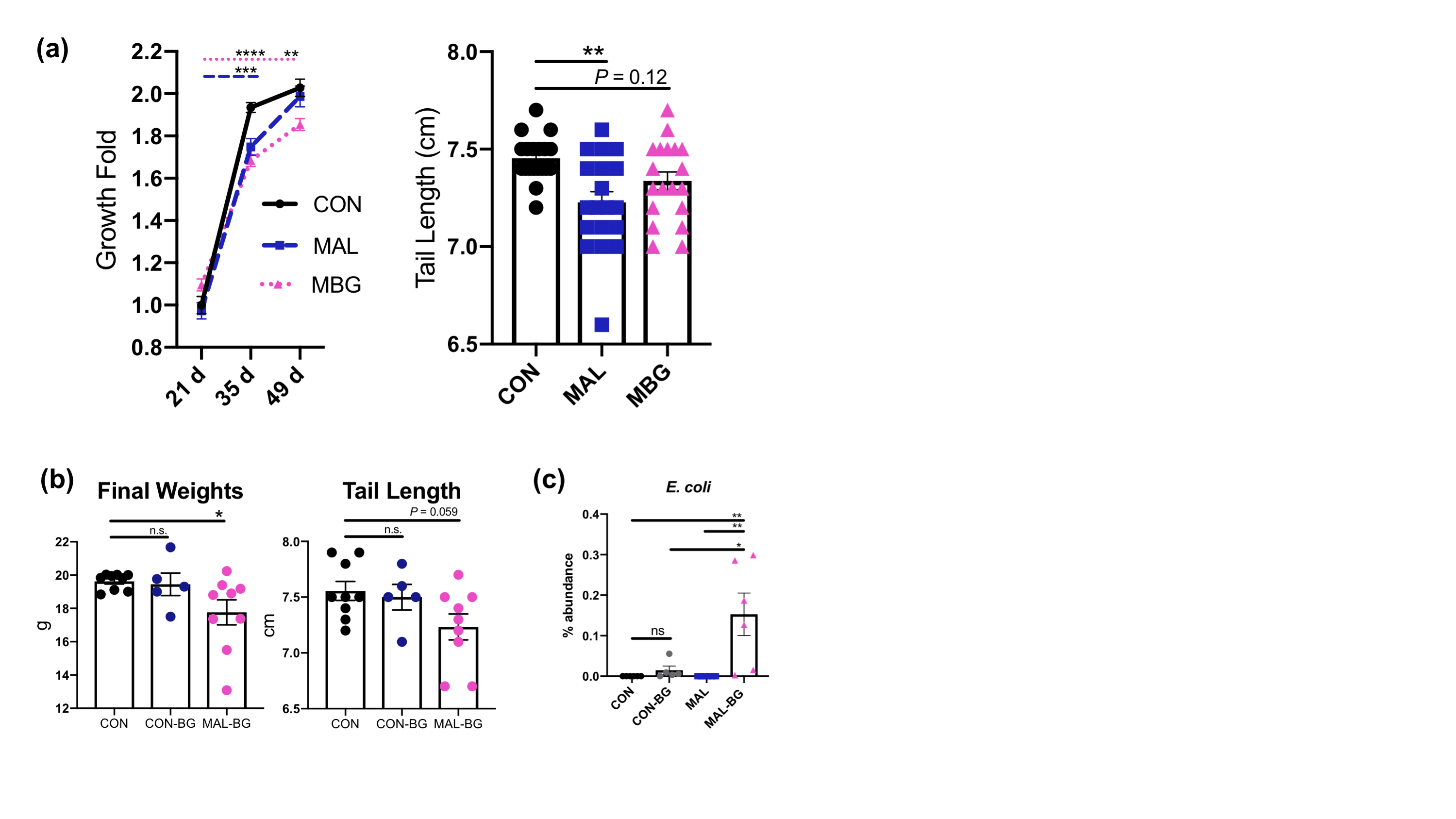

Supplement: Supplementary file 5 — Figure S1 Fecal‐oral contamination impacts growth during malnutrition (a) Microbial exposure promoted weight faltering in malnourished mice (n = 19). Tail length, a proxy for stunting, is reduced in malnourished mice. Panels from same experiment. (b) Microbial E. coli/bacteroidales exposures failed to trigger weight and stunting features in healthy mice (n = 9 CON, 5 CON‐BG, 9 MAL‐BG). Panels from same experiment. (c) The relative abundance of Enterobacteriaceae from fecal samples measured by qPCR, similar findings reported in (Brown et al., 2015). Bar graphs indicate mean and SEM with statistical significance determined by one‐way ANOVA with post hoc Dunnett's test [file GLIA-70-820-s012.tif]

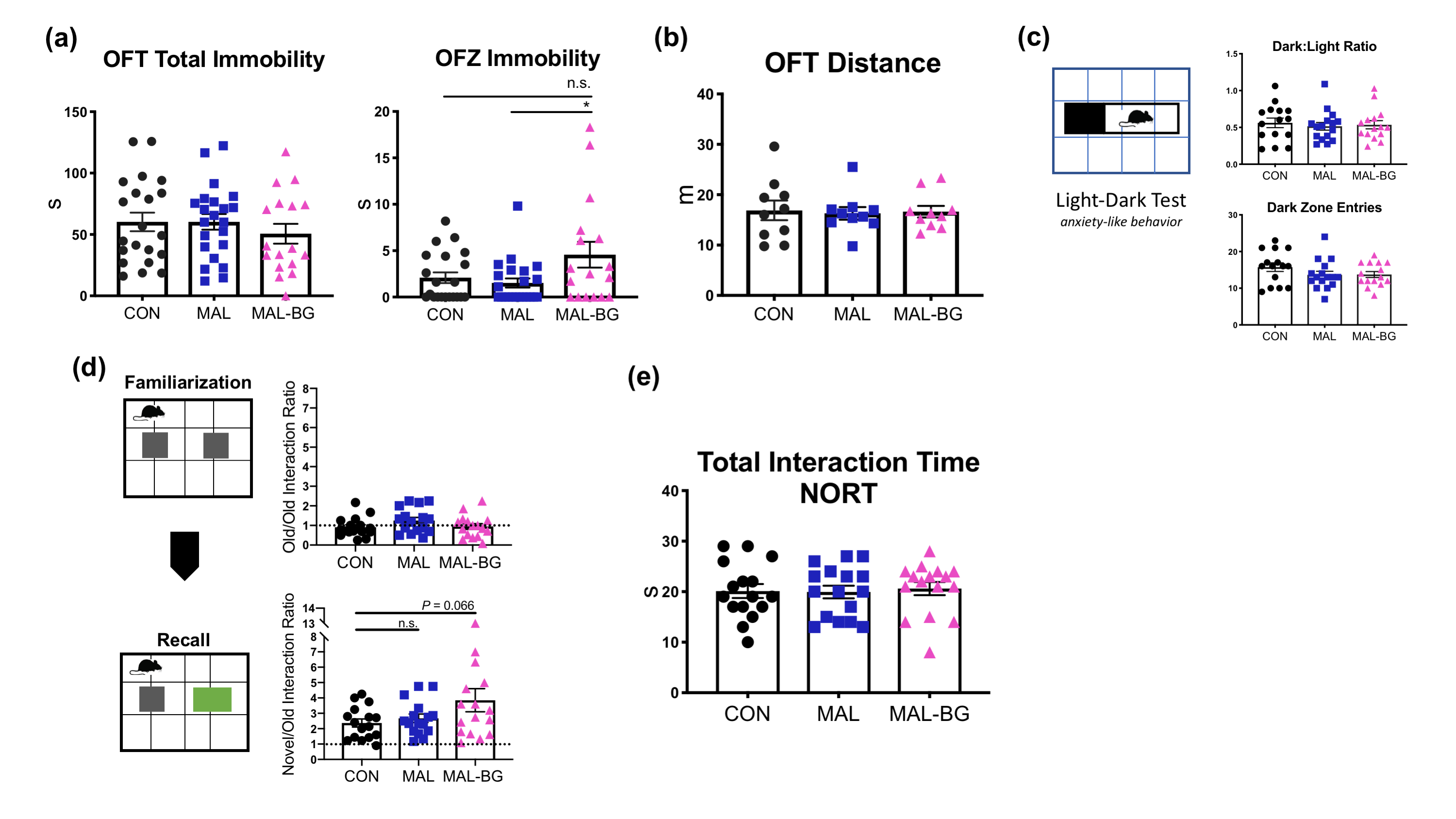

Supplement: Supplementary file 6 — Figure S2 Malnutrition and microbes influence behavior and cognition in MAL‐BG model (a) Total immobility “resting” (left) and OFZ immobility (right) during the OFT, n = 21 CON, 22 MAL, 17 MAL‐BG. (b) Total distance traveled, representative OFT experiment. (c) Set‐up (left) and results (right) from the light–dark test. A measure of anxiety‐like behavior, the light–dark box is comprised of an open light region and enclosed dark region. CON, MAL, and MAL‐BG exhibit comparable behavior within the light–dark box. (d) NORT schematic showing the familiarization and recall set‐up. During familiarization, mice exhibit impartial object exploration (interaction ratio ~ 1). All groups distinguished the novel object during recall (novel: old interaction ratio > 1). NORT interactions were scored by a blinded observer. (e) Total mouse‐object interaction time (novel and old object interaction) for the NORT as recorded by a blinded observer. (f) MWMT set‐up: during habituation, mice were released from the same position and learned to locate a visible platform, platform location moved after each trial. During learning phases (acquisition, reversal) individual mice attempted to locate a hidden platform based on spatial memory and external cues. Mice entered the pool at variable locations (north, south, east, and west quadrants); trial order and location entries were randomized prior to testing. Habituation and learning trials lasted 60 s each with a rest period. Individual mice were placed in an empty MWM (30 s swim) 24 h following the final acquisition and reversal trial. (g) Average swim speed for the initial (top) and final (bottom) free swims. (h) The escape latency for the initial reversal learning trial. Mice that failed to locate the new platform location within 60 s were gently guided to the platform, following trial (failed trials represented at the dotted line). (i) Total entries to the prior platform location of each mouse across four trials, for the 1st (top) and 2nd (bot [file GLIA-70-820-s005.zip › GLIA_24139_FigureS2a.tif]

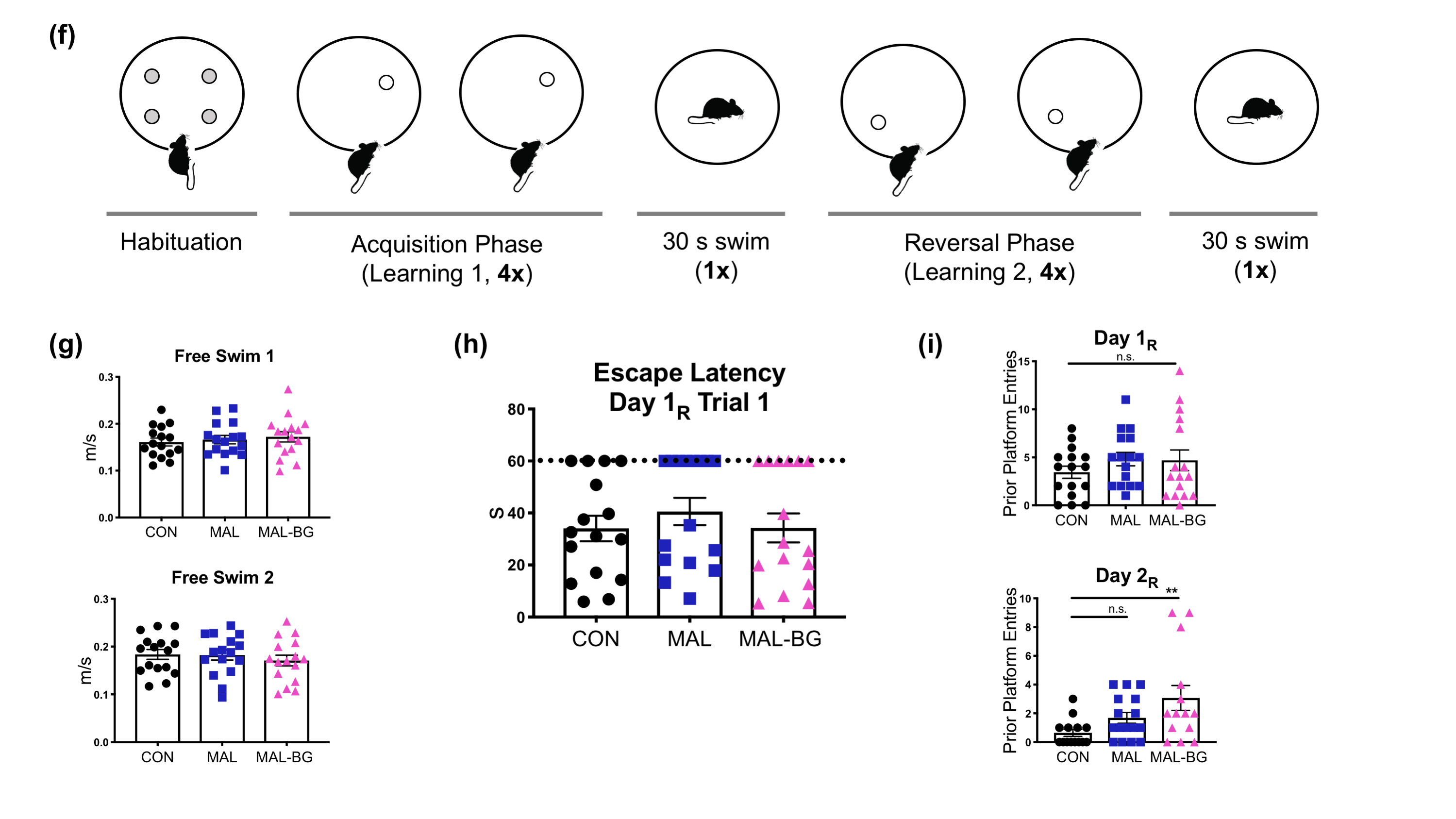

Supplement: Supplementary file 6 — Figure S2 Malnutrition and microbes influence behavior and cognition in MAL‐BG model (a) Total immobility “resting” (left) and OFZ immobility (right) during the OFT, n = 21 CON, 22 MAL, 17 MAL‐BG. (b) Total distance traveled, representative OFT experiment. (c) Set‐up (left) and results (right) from the light–dark test. A measure of anxiety‐like behavior, the light–dark box is comprised of an open light region and enclosed dark region. CON, MAL, and MAL‐BG exhibit comparable behavior within the light–dark box. (d) NORT schematic showing the familiarization and recall set‐up. During familiarization, mice exhibit impartial object exploration (interaction ratio ~ 1). All groups distinguished the novel object during recall (novel: old interaction ratio > 1). NORT interactions were scored by a blinded observer. (e) Total mouse‐object interaction time (novel and old object interaction) for the NORT as recorded by a blinded observer. (f) MWMT set‐up: during habituation, mice were released from the same position and learned to locate a visible platform, platform location moved after each trial. During learning phases (acquisition, reversal) individual mice attempted to locate a hidden platform based on spatial memory and external cues. Mice entered the pool at variable locations (north, south, east, and west quadrants); trial order and location entries were randomized prior to testing. Habituation and learning trials lasted 60 s each with a rest period. Individual mice were placed in an empty MWM (30 s swim) 24 h following the final acquisition and reversal trial. (g) Average swim speed for the initial (top) and final (bottom) free swims. (h) The escape latency for the initial reversal learning trial. Mice that failed to locate the new platform location within 60 s were gently guided to the platform, following trial (failed trials represented at the dotted line). (i) Total entries to the prior platform location of each mouse across four trials, for the 1st (top) and 2nd (bot [file GLIA-70-820-s005.zip › GLIA_24139_FigureS2b.tif]

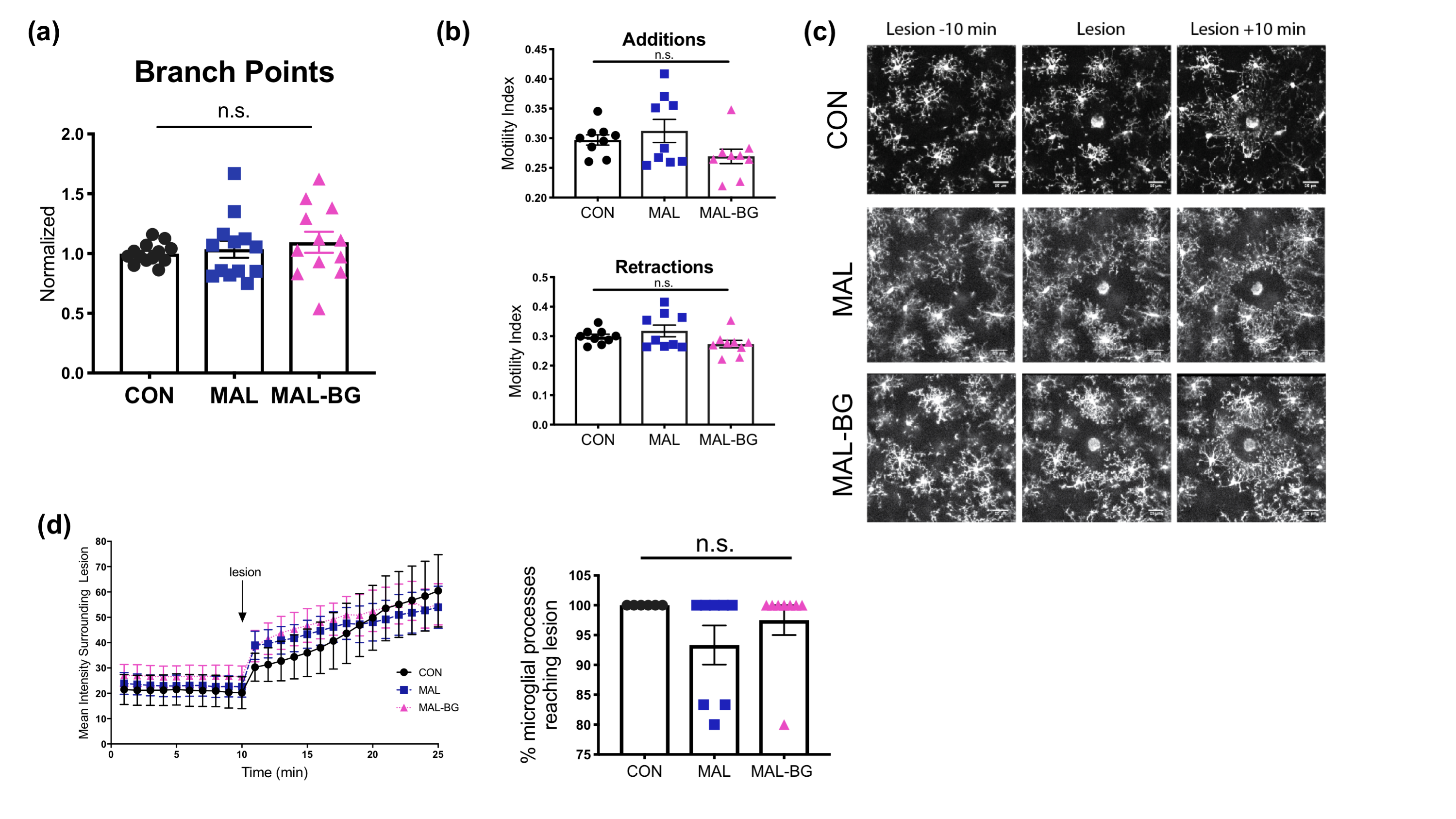

Supplement: Supplementary file 7 — Figure S3 Microglial morphology and motility characterization (a) MAL and MAL‐BG hippocampal microglial volume alterations were independent of process branching. 3DMorph analyses with data normalized to the controls from four independent experiments, n = 13 CON, 13 MAL, and 12 MAL‐BG. (b) Averaged microglial process additions and retractions across 10 min in ex vivo CX3CR1+/EGFP CON, MAL, and MAL‐BG hippocampal tissues, n = 9/group. Motility indices determined by a custom MATLAB program that identified pixel additions/removal in eGFP+ cells. (c) Representative images from two‐photon microscopy of the hippocampal CA1 region prior, at, and following lesion induction via intensive two‐photon laser scanning, n = 6 CON, 8 MAL, and 8 MAL‐BG. Videos in Supplemental File 2 (CON image from same animal in video file). (d) Microglial process response to lesion region: mean fluorescent intensity/microscopy frames (left) and % microglial processes entering lesion region at experimental endpoint (right). Lesion experiments conducted on a subset of mice utilized for microglial morphology analyses. Mice in (b–d) a subset from experiments presented in a. Graphs indicate mean and SEM with statistical significance determined by one‐way ANOVA with post hoc Tukey's test; n.s., non‐significant [file GLIA-70-820-s007.tif]

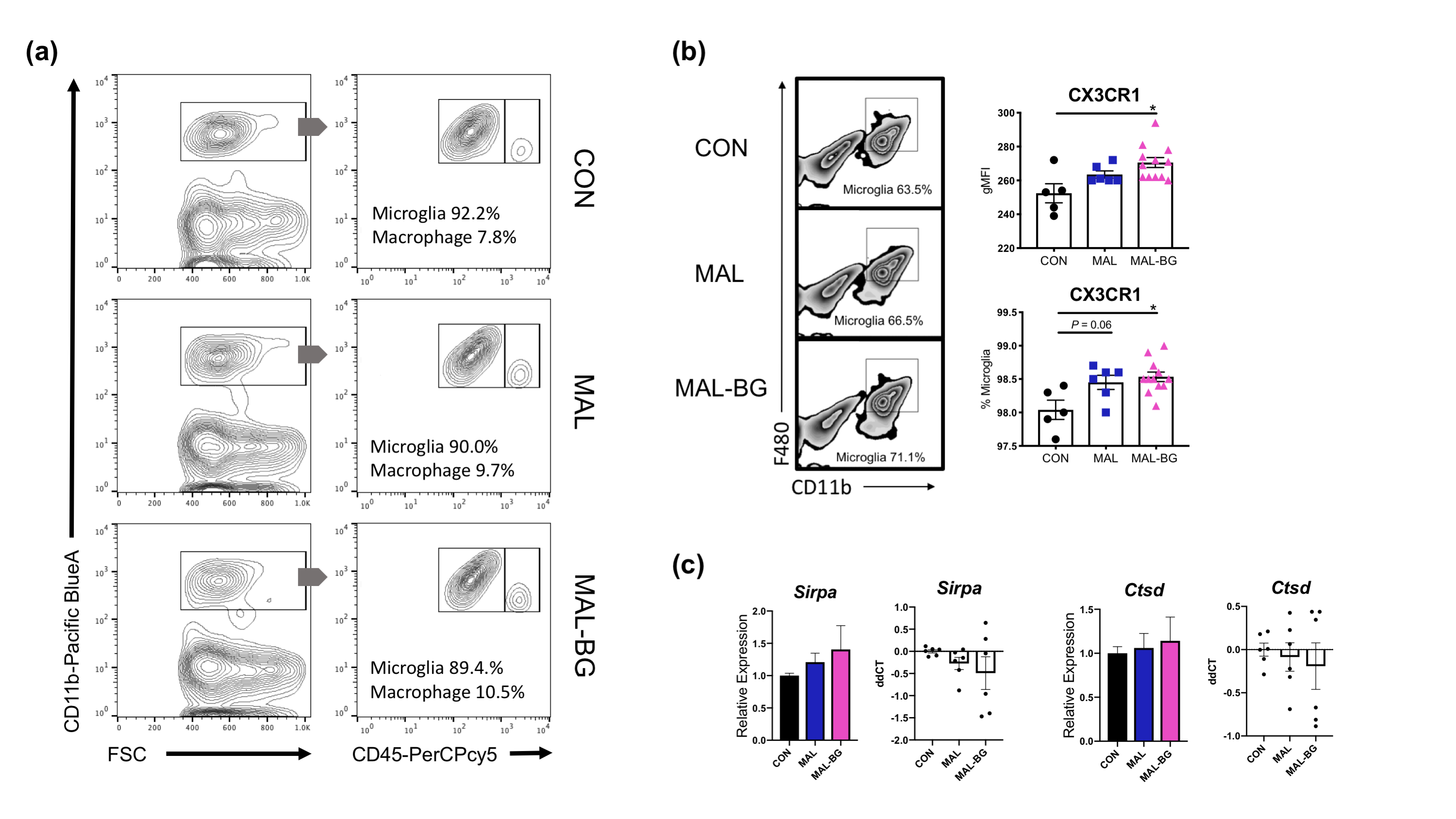

Supplement: Supplementary file 8 — Figure S4 Altered functional profile in MAL‐BG microglia (a) RNA‐Seq was conducted on CD11b+ population from whole brain tissue, n = 4 CON, 5 MAL, and 7 MAL‐BG. Representative flow cytometry gating verifying microglial enrichment (CD11bhigh/CD45low population) following Miltenyi Biotec Adult Brain Dissociation kit and CD11b separation. (b) Representative flow cytometry gating (left) of CX3CR1 gMFI and frequency (% microglia) from an independent cohort (right), supporting RNA‐Seq findings, n = 5 CON, 6 MAL, 12 MAL‐BG. (c) Whole brain qPCR results from an independent mouse cohort: while assessed DEGs did not reach statistical significance by RT‐qPCR, overall patterns support microglial Sirpa (phagocytic marker) and Ctsd (lysosomal marker) RNA‐Seq results. Fold change and ddct values plotted, Hprt provided the endogenous control. (d) ReactomePA network enrichment visualizations for MAL‐BG versus CON (left) and CON versus MAL‐BG (right): Padj < 0.05, FC >1.5, data from RNA‐Seq experiment in a. Bar graphs indicate mean and SEM with statistical significance determined by Kruskal‐Wallis with post hoc Dunn's test [file GLIA-70-820-s011.zip › GLIA_24139_FigureS4a.tif]

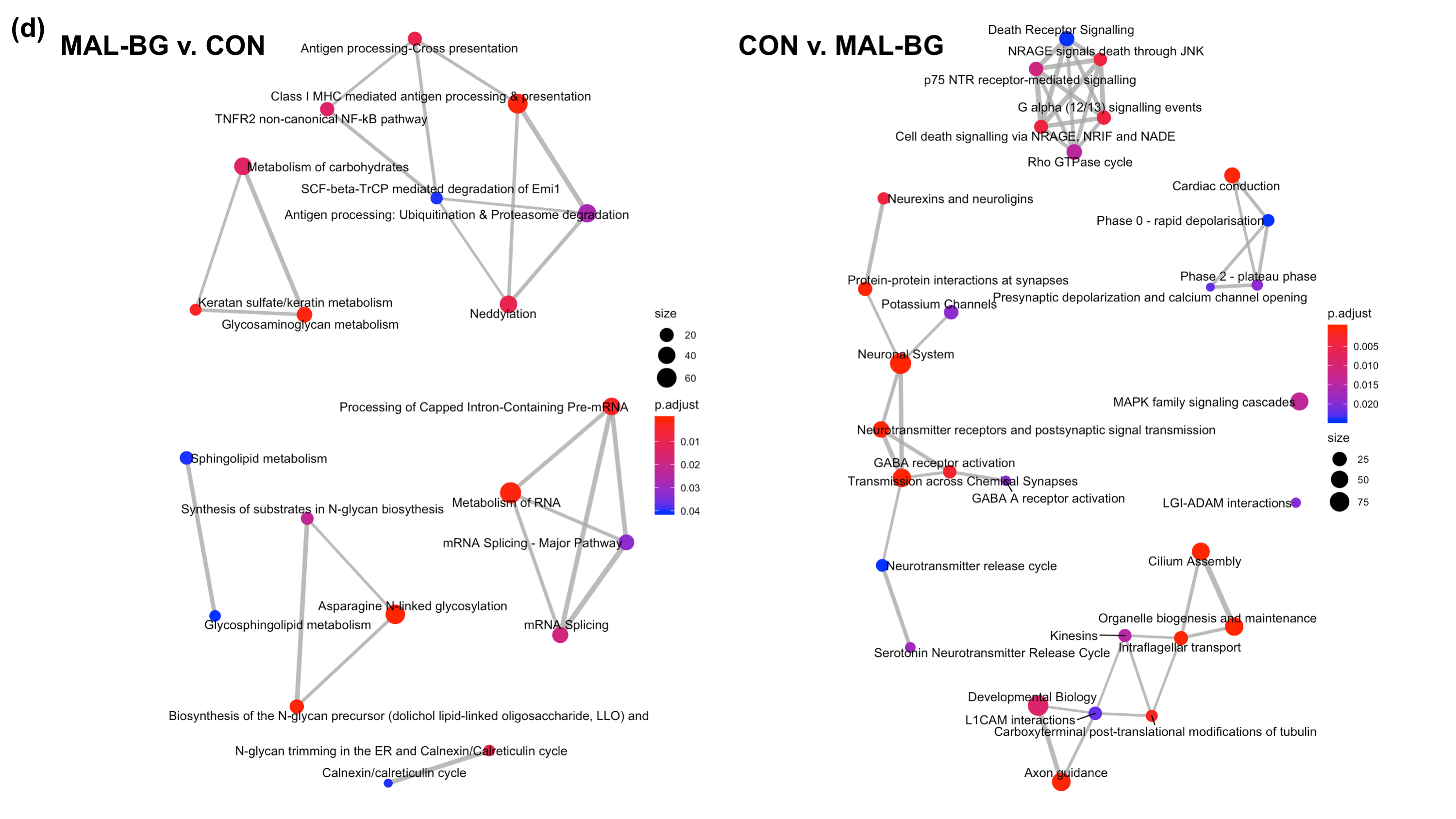

Supplement: Supplementary file 8 — Figure S4 Altered functional profile in MAL‐BG microglia (a) RNA‐Seq was conducted on CD11b+ population from whole brain tissue, n = 4 CON, 5 MAL, and 7 MAL‐BG. Representative flow cytometry gating verifying microglial enrichment (CD11bhigh/CD45low population) following Miltenyi Biotec Adult Brain Dissociation kit and CD11b separation. (b) Representative flow cytometry gating (left) of CX3CR1 gMFI and frequency (% microglia) from an independent cohort (right), supporting RNA‐Seq findings, n = 5 CON, 6 MAL, 12 MAL‐BG. (c) Whole brain qPCR results from an independent mouse cohort: while assessed DEGs did not reach statistical significance by RT‐qPCR, overall patterns support microglial Sirpa (phagocytic marker) and Ctsd (lysosomal marker) RNA‐Seq results. Fold change and ddct values plotted, Hprt provided the endogenous control. (d) ReactomePA network enrichment visualizations for MAL‐BG versus CON (left) and CON versus MAL‐BG (right): Padj < 0.05, FC >1.5, data from RNA‐Seq experiment in a. Bar graphs indicate mean and SEM with statistical significance determined by Kruskal‐Wallis with post hoc Dunn's test [file GLIA-70-820-s011.zip › GLIA_24139_FigureS4b.tif]

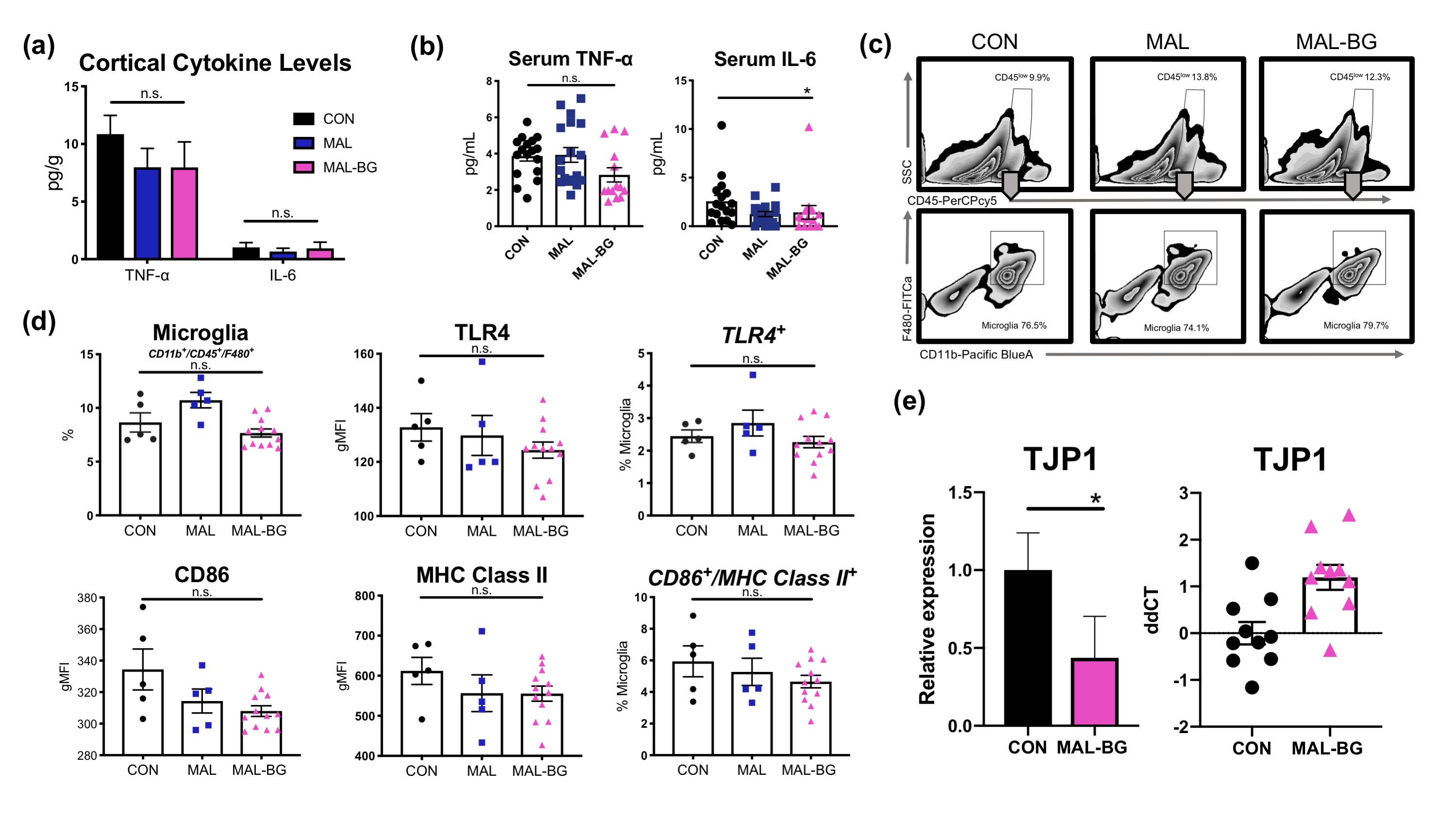

Supplement: Supplementary file 9 — Figure S5 The MAL‐BG brain lacks significant neuroinflammation and BBB disruption (a) Proinflammatory cytokines (TNF‐α, IL‐6) from cortical brain tissues, cytokine levels normalized to tissue weight (n = 19 CON, 21 MAL, 16–17 MAL‐BG), data from three independent experiments. (b) TNF‐α and IL‐6 levels in CON, MAL, and MAL‐BG sera, data from two independent experiments, n = 17 CON, 18 MAL, 14 MAL‐BG. (c) Representative flow cytometry gating for inflammatory microglia panel. Microglia reported as CD11bhigh/F480high within a CD45low cell population. (d) Percent microglia of CON, MAL, and MAL‐BG cells following microglia isolation. Microglia identified as CD11bhigh/F480high within a CD45low cell population. TLR4, CD86, and MHC Class II gMFI and frequency (% microglia) presented. (e) MAL‐BG mice exhibit a decrease in Tjp1 (tight junction protein 1, zonula occludens 1) expression; qPCR from murine small intestine (ileal tissue), n = 10. Detailed GI permeability measures presented in Brown et al., 2015. (f) Quantification of IgG immunostaining revealed low levels of IgG antibodies within the brain parenchyma, n = 4/experimental group. High‐IgG presence in control photothrombotic brain tissue (ischemic stroke model), data not reported. (g) Averaged biocytin fluorescent intensity following biocytin‐TMR tail‐vein injection. (h) Biocytin‐TMR intensity across murine CNS slices, each line represents a mouse, symbols denote slice, n = 4/group. (i) Representative CON, MAL, and MAL‐BG cortical slices (bottom) with matched rostral ➔ caudal CNS images, biocytin‐TMR appears white. IgG and biocytin data from the same experimental dataset. Bar graphs indicate mean and SEM with statistical significance determined by Mann–Whitney two‐tailed test (qPCR), Kruskal‐Wallis with post hoc Dunn's test (flow cytometry), or one‐way ANOVA with post hoc Dunnett's test (cytokines, IgG, biocytin); CBLM, cerebellum; gMFI, geometric mean fluorescence intensity; n.s., not significant; PFC, prefrontal corte [file GLIA-70-820-s004.zip › GLIA_24139_FigureS5a.tif]

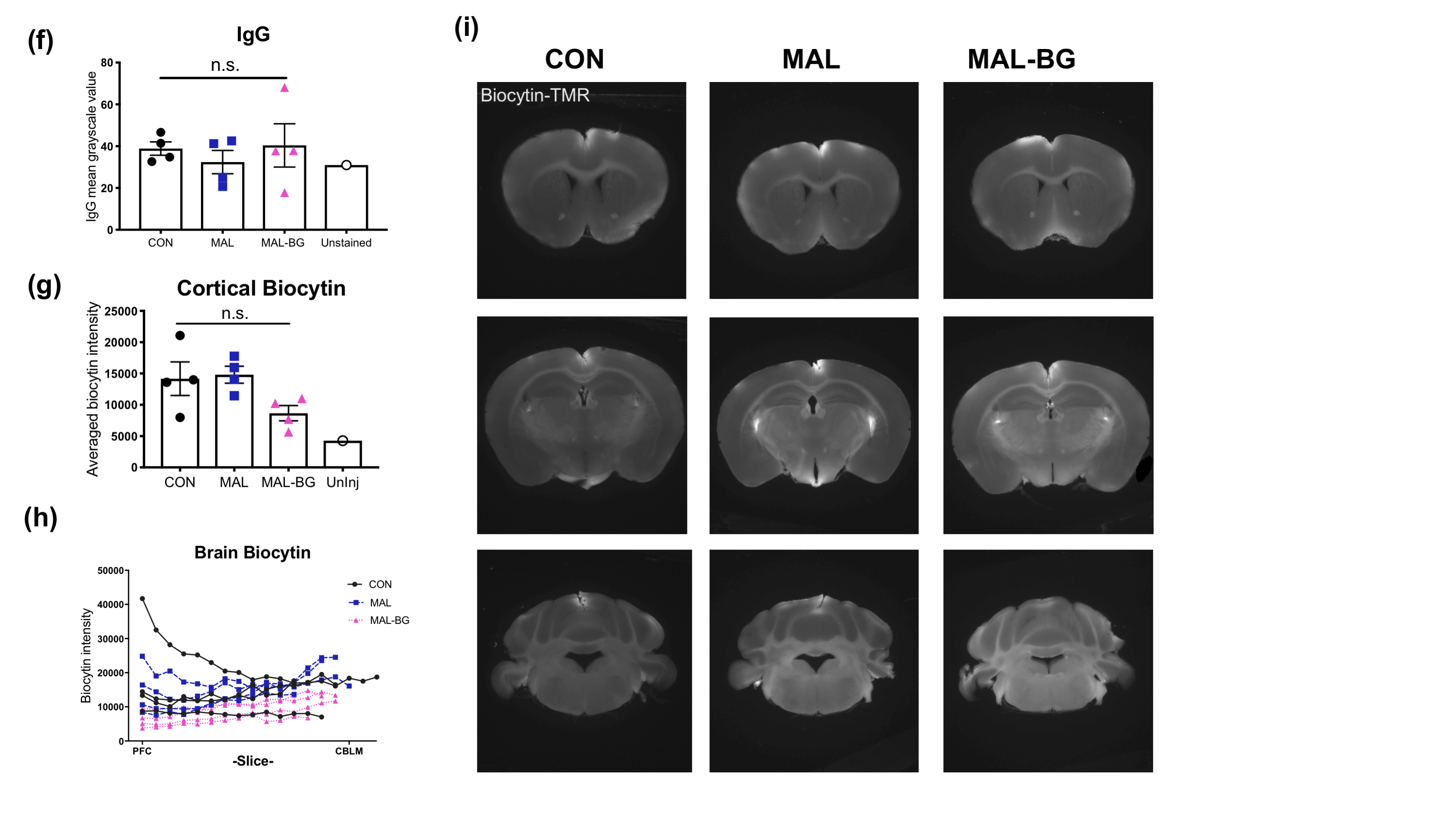

Supplement: Supplementary file 9 — Figure S5 The MAL‐BG brain lacks significant neuroinflammation and BBB disruption (a) Proinflammatory cytokines (TNF‐α, IL‐6) from cortical brain tissues, cytokine levels normalized to tissue weight (n = 19 CON, 21 MAL, 16–17 MAL‐BG), data from three independent experiments. (b) TNF‐α and IL‐6 levels in CON, MAL, and MAL‐BG sera, data from two independent experiments, n = 17 CON, 18 MAL, 14 MAL‐BG. (c) Representative flow cytometry gating for inflammatory microglia panel. Microglia reported as CD11bhigh/F480high within a CD45low cell population. (d) Percent microglia of CON, MAL, and MAL‐BG cells following microglia isolation. Microglia identified as CD11bhigh/F480high within a CD45low cell population. TLR4, CD86, and MHC Class II gMFI and frequency (% microglia) presented. (e) MAL‐BG mice exhibit a decrease in Tjp1 (tight junction protein 1, zonula occludens 1) expression; qPCR from murine small intestine (ileal tissue), n = 10. Detailed GI permeability measures presented in Brown et al., 2015. (f) Quantification of IgG immunostaining revealed low levels of IgG antibodies within the brain parenchyma, n = 4/experimental group. High‐IgG presence in control photothrombotic brain tissue (ischemic stroke model), data not reported. (g) Averaged biocytin fluorescent intensity following biocytin‐TMR tail‐vein injection. (h) Biocytin‐TMR intensity across murine CNS slices, each line represents a mouse, symbols denote slice, n = 4/group. (i) Representative CON, MAL, and MAL‐BG cortical slices (bottom) with matched rostral ➔ caudal CNS images, biocytin‐TMR appears white. IgG and biocytin data from the same experimental dataset. Bar graphs indicate mean and SEM with statistical significance determined by Mann–Whitney two‐tailed test (qPCR), Kruskal‐Wallis with post hoc Dunn's test (flow cytometry), or one‐way ANOVA with post hoc Dunnett's test (cytokines, IgG, biocytin); CBLM, cerebellum; gMFI, geometric mean fluorescence intensity; n.s., not significant; PFC, prefrontal corte [file GLIA-70-820-s004.zip › GLIA_24139_FigureS5b.tif]

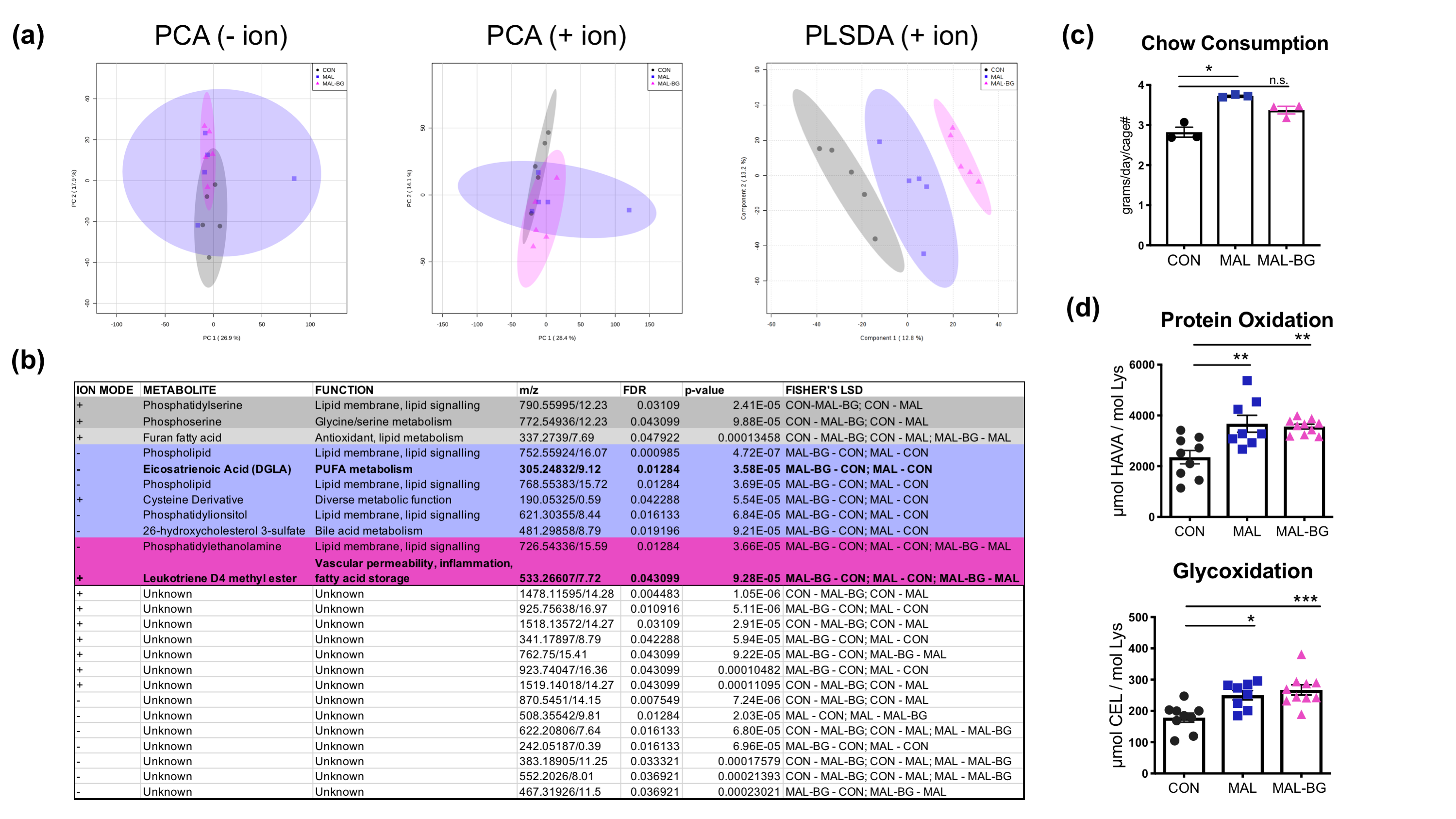

Supplement: Supplementary file 10 — Figure S6 Altered hippocampal metabolomics and PUFA metabolism in malnourished mice (a) PCA (+/− ion channels) and PLSDA (+ ion channel) of untargeted metabolomics from murine hippocampi, n = 5/group. (b) Putative m/z identification for differentially abundant metabolites determined by Metaboanalyst v. 3.0/4.0 (one‐way ANOVA, Padj < 0.05, post‐hoc Fischer's LSD), features annotated against KEGG and METLIN databases. (c) Chow weights/day normalized to number of mice per cage. Chow consumption data from three cages (n = 9/group), averaged across three 24 h timepoints, each symbol represents a cage. (d) HAVA and CEL levels from murine cortical tissue. Data normalized to tissue mol lysine. (n = 7–10/group). Bar graphs indicate mean and SEM with statistical significance determined by Kruskal‐Wallis post hoc Dunn's test (chow consumption) or one‐way ANOVA with post hoc Tukey's test (oxidative stress) [file GLIA-70-820-s001.tif]

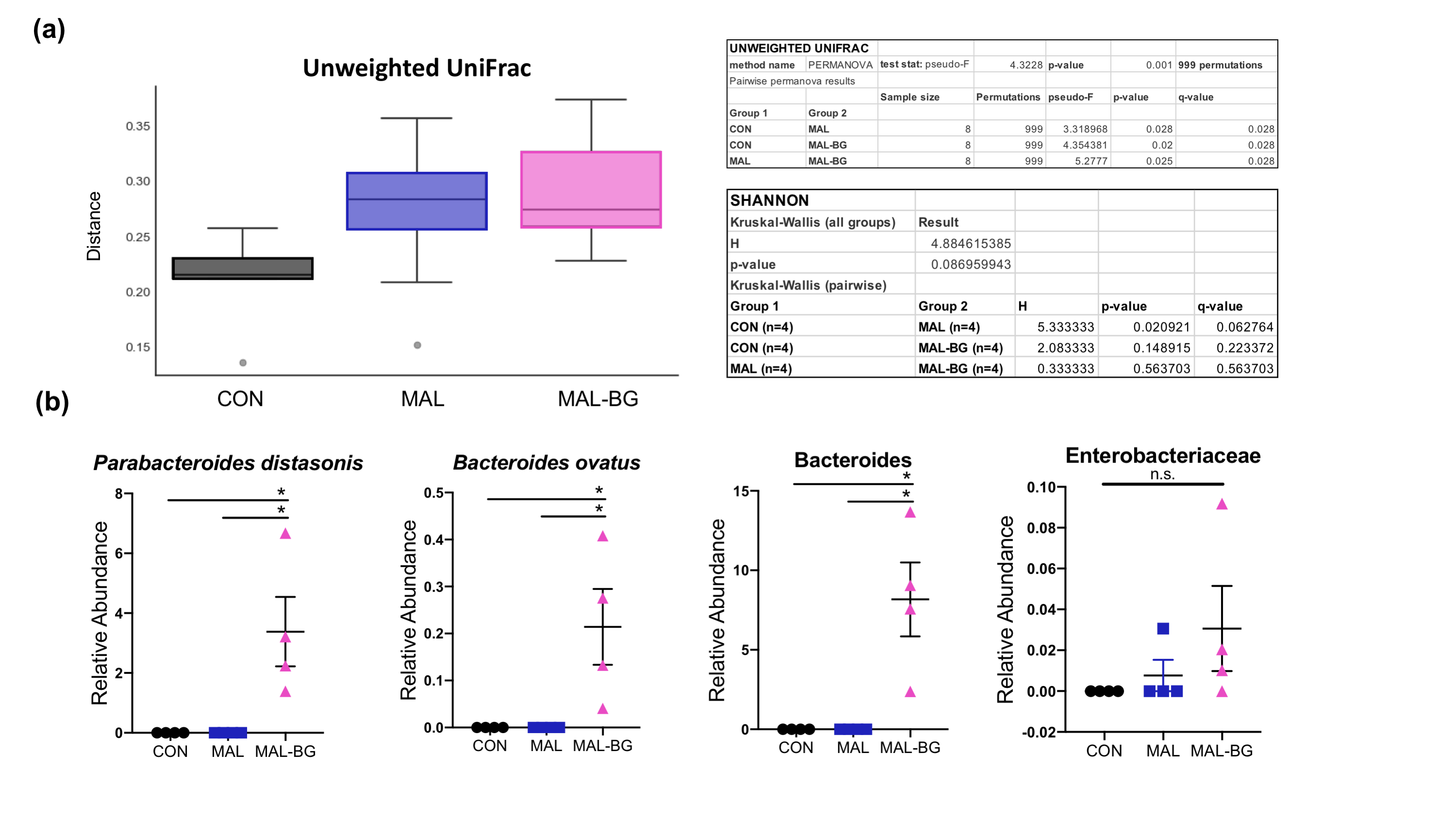

Supplement: Supplementary file 11 — Figure S7 Diet and fecal‐oral contamination alter gut microbiota and promote systemic oxidative stress (a) β (Unweighted UniFrac) and α (Shannon) diversity analyses from 16S rRNA fecal microbiota data, microbiome analyses conducted using QIIME2 (v. 2018.2), n = 4/group. (b) Relative abundance of MAL‐BG gavage components, including Parabacteroides distasonis and Bacteroides ovatus, as well as the Bacteroides genus and Enterobacteriaceae family (E. coli family). (c) PICRUSt analyses to explore functionality shifts between MAL and MAL‐BG fecal microbiome, pathways annotated with MetaCyc (lower left table), only Padj < 0.01 presented, full PICRUSt output in Supplemental File 3b. Microbiome analyses from the same experiment. Graphs presented in b indicate mean and SEM with statistical significance determined by Kruskal‐Wallis with post hoc Dunn's test; CI, confidence intervals; n.s., not significant [file GLIA-70-820-s002.zip › GLIA_24139_FigureS7a.tif]

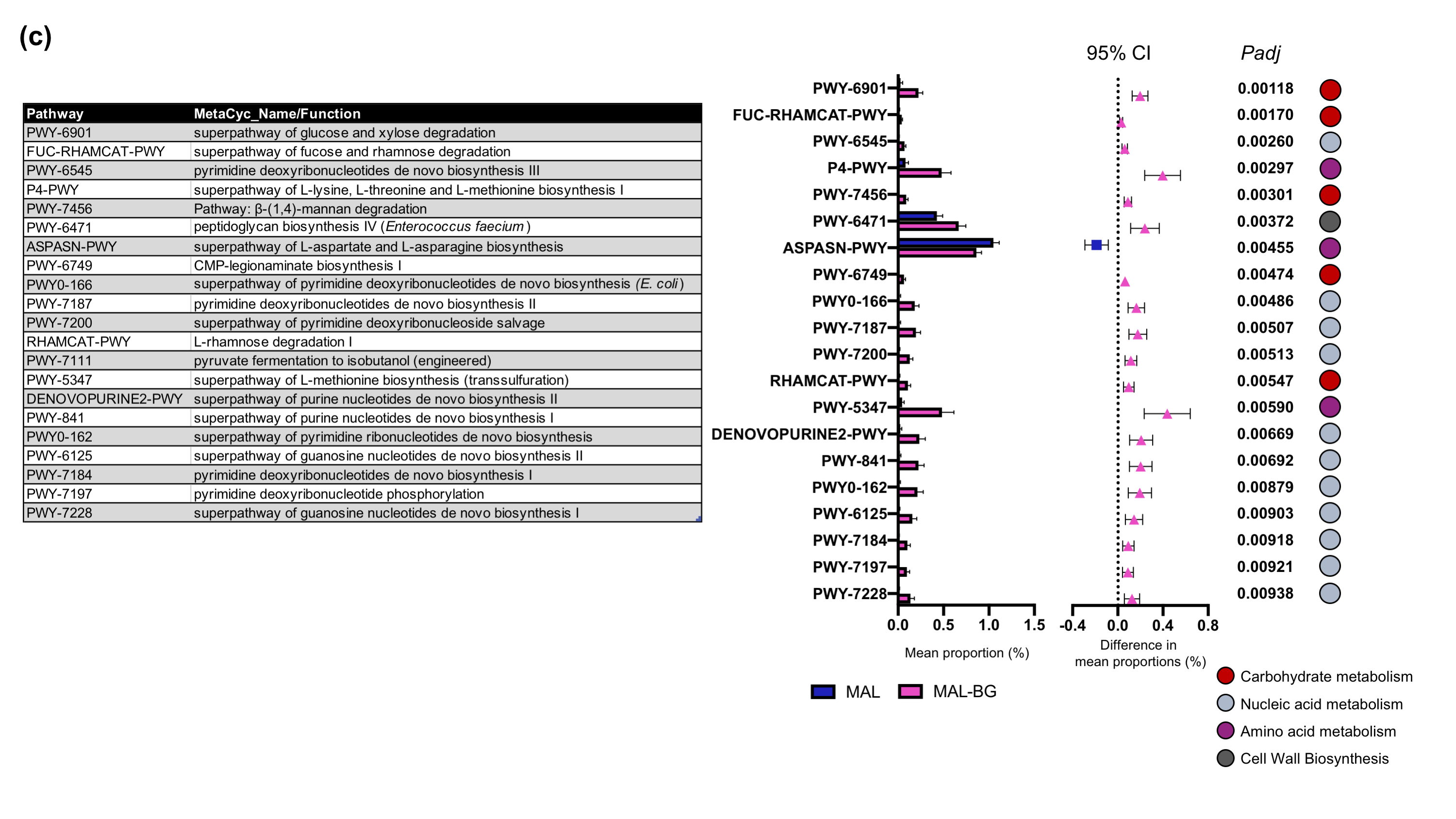

Supplement: Supplementary file 11 — Figure S7 Diet and fecal‐oral contamination alter gut microbiota and promote systemic oxidative stress (a) β (Unweighted UniFrac) and α (Shannon) diversity analyses from 16S rRNA fecal microbiota data, microbiome analyses conducted using QIIME2 (v. 2018.2), n = 4/group. (b) Relative abundance of MAL‐BG gavage components, including Parabacteroides distasonis and Bacteroides ovatus, as well as the Bacteroides genus and Enterobacteriaceae family (E. coli family). (c) PICRUSt analyses to explore functionality shifts between MAL and MAL‐BG fecal microbiome, pathways annotated with MetaCyc (lower left table), only Padj < 0.01 presented, full PICRUSt output in Supplemental File 3b. Microbiome analyses from the same experiment. Graphs presented in b indicate mean and SEM with statistical significance determined by Kruskal‐Wallis with post hoc Dunn's test; CI, confidence intervals; n.s., not significant [file GLIA-70-820-s002.zip › GLIA_24139_FigureS7b.tif]

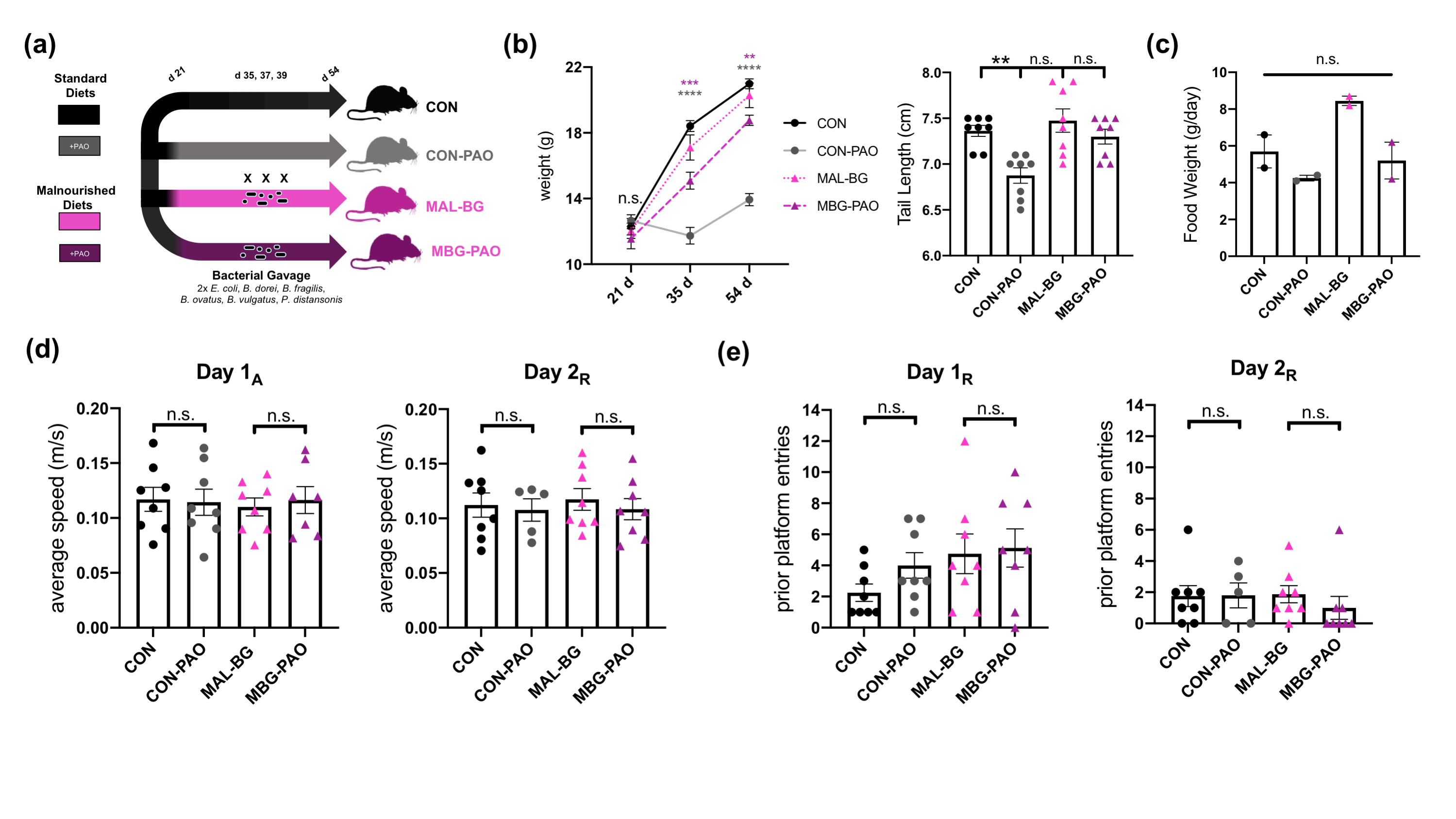

Supplement: Supplementary file 12 — Figure S8 PAO diet shapes cognition and systemic PUFA profiling (a) Model of dietary PAO (ω3 PUFA/antioxidant‐associated supplement) intervention. A subset of the CON and MAL‐BG models (MBG) were placed on isocaloric CON‐PAO and MBG‐PAO diets. The PAO diet; however, contained an ω3 PUFA fat source and was enriched with elevated antioxidant‐associated vitamins, see Supplemental File 1 for full dietary breakdown. Data was assessed 54 days following start of the diet. (b) PAO mice displayed increased weight faltering (left) and stunting (tail length: right), compared to respective controls. (c) Chow consumption data from two cages (n = 8/group) across a 24 h timepoint approximately halfway through study. (d) Swim speed averaged across four trials from Day 1A (initial acquisition learning: left) and final experimental day (Day 2R: right). (e) Total entries within the prior platform area for each mouse/day of reversal learning (left = Day 1R left, right = Day 2R). (f) Microglial morphology (cell volume, territory volume, and endpoints) quantified with 3DMorph software and normalized to the CON group. (g) Percent of mice that exhibited microglial phagocytic feature/s within hippocampal regions (h) Relative PUFA abundance from cortical tissue normalized to CON mice. (i) Ratio of mol% ω3/ω6 PUFA levels from liver tissue. (j) Oxidative profiling from cortical tissue: HAVA (protein oxidation), CEL (glycoxidation), CML (glycoxidation/lipoxidation), and MDAL (PUFA lipoxidation). Data normalized to mol lysine. Panels from same experimental round (n = 5–8/group). Bar graphs present mean and SEM with statistical significance determined by one‐way ANOVA with post hoc Tukey's test (MWMT, microglia, fatty acid analyses) or Kruskal‐Wallis post hoc Dunn's test (chow consumption): MWMT, Morris water maze test; n.s., not significant [file GLIA-70-820-s010.zip › GLIA_24139_FigureS8a.tif]

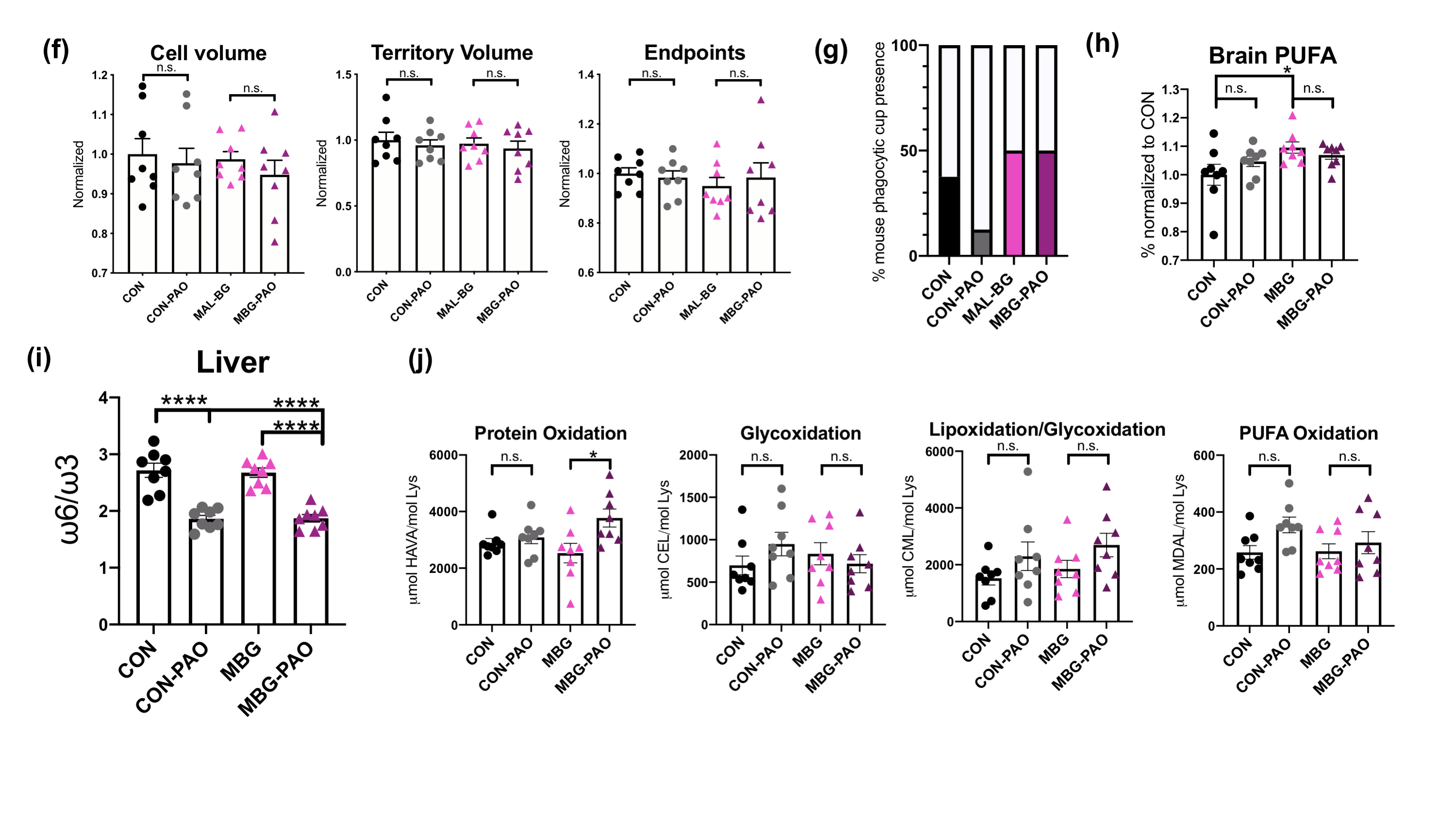

Supplement: Supplementary file 12 — Figure S8 PAO diet shapes cognition and systemic PUFA profiling (a) Model of dietary PAO (ω3 PUFA/antioxidant‐associated supplement) intervention. A subset of the CON and MAL‐BG models (MBG) were placed on isocaloric CON‐PAO and MBG‐PAO diets. The PAO diet; however, contained an ω3 PUFA fat source and was enriched with elevated antioxidant‐associated vitamins, see Supplemental File 1 for full dietary breakdown. Data was assessed 54 days following start of the diet. (b) PAO mice displayed increased weight faltering (left) and stunting (tail length: right), compared to respective controls. (c) Chow consumption data from two cages (n = 8/group) across a 24 h timepoint approximately halfway through study. (d) Swim speed averaged across four trials from Day 1A (initial acquisition learning: left) and final experimental day (Day 2R: right). (e) Total entries within the prior platform area for each mouse/day of reversal learning (left = Day 1R left, right = Day 2R). (f) Microglial morphology (cell volume, territory volume, and endpoints) quantified with 3DMorph software and normalized to the CON group. (g) Percent of mice that exhibited microglial phagocytic feature/s within hippocampal regions (h) Relative PUFA abundance from cortical tissue normalized to CON mice. (i) Ratio of mol% ω3/ω6 PUFA levels from liver tissue. (j) Oxidative profiling from cortical tissue: HAVA (protein oxidation), CEL (glycoxidation), CML (glycoxidation/lipoxidation), and MDAL (PUFA lipoxidation). Data normalized to mol lysine. Panels from same experimental round (n = 5–8/group). Bar graphs present mean and SEM with statistical significance determined by one‐way ANOVA with post hoc Tukey's test (MWMT, microglia, fatty acid analyses) or Kruskal‐Wallis post hoc Dunn's test (chow consumption): MWMT, Morris water maze test; n.s., not significant [file GLIA-70-820-s010.zip › GLIA_24139_FigureS8b.tif]
